# Prediabetes increases the risk of pancreatic cancer: A meta-analysis of longitudinal observational studies

**DOI:** 10.1371/journal.pone.0311911

**Published:** 2024-10-15

**Authors:** Xuefang Huang, Huan Li, Lisha Zhao, Lingli Xu, Hui Long

**Affiliations:** Department of Gastroenterology, Tianyou Hospital, Wuhan University of Science and Technology, Wuhan, Hubei, China; Tehran University of Medical Sciences, ISLAMIC REPUBLIC OF IRAN

## Abstract

**Background:**

Glycemic disorder is closely related to the risk of pancreatic cancer, but previous studies focused on the influence of diabetes. The aim of this meta-analysis was to investigate the influence of prediabetes, an intermediate state between normoglycemia and diabetes, on the risk of pancreatic cancer.

**Methods:**

Relevant longitudinal observational studies were identified through a search of Medline, Embase, and Web of Science databases. To minimize the influence of between-study heterogeneity, a randomized-effects model was used to pool the results.

**Results:**

Nine cohort studies including 26,444,624 subjects were available for the meta-analysis. Among them, 2,052,986 (7.8%) had prediabetes at baseline, and the participants were followed for a mean duration of 5.9 years. It was found that, compared to people with normoglycemia, those with prediabetes had a higher incidence of pancreatic cancer (risk ratio [RR]: 1.42, 95% confidence interval: 1.36 to 1.49, *p*<0.001) with no statistical heterogeneity (I^2^ = 0%). Sensitivity analysis performed by excluding one dataset at a time did not significantly change the results (RR: 1.38 to 1.45, *p* all <0.05). Subgroup analyses indicated that the association between prediabetes and increased risk of pancreatic cancer was not significantly impacted by study characteristics such as study design, location, age, and sex of participants, definition of prediabetes, duration of follow-up, or adjustment for alcohol intake (*p* for subgroup difference all >0.05).

**Conclusions:**

Prediabetes may be associated with an increased risk of pancreatic cancer compared to normoglycemia.

## Introduction

Pancreatic cancer is one of the most lethal malignancies worldwide, with a 5-year survival rate of approximately 10% [1–3]. Despite advances in treatment modalities, the prognosis for pancreatic cancer remains poor, emphasizing the critical need for effective prevention and early detection strategies [4]. Epidemiological studies have identified several risk factors associated with pancreatic cancer, including smoking, alcohol drinking, obesity, chronic pancreatitis, and family history of pancreatic cancer [4,5].

In recent years, attention has turned to the role of glycemic disorders in the development of cancer. Diabetes mellitus [6–9] and gestational diabetes mellitus [10] have been consistently linked to an increased risk of pancreatic cancer. However, the association between prediabetes, an intermediate state between normoglycemia and diabetes, and pancreatic cancer risk remains uncertain [11]. Prediabetes is characterized by elevated blood glucose levels that do not meet the diagnostic criteria for diabetes, which could be defined by impaired fasting glucose (IFG), impaired glucose tolerance (IGT), and mildly elevated hemoglobin A1c (HbA1c) [12]. Although IGT is consistently defined as a 2 hour plasma glucose concentration of 7.8–11.0 mmol/L during an oral glucose tolerance test, the definitions of IFG differ between those of the World Health Organization (WHO) criteria (fasting plasma glucose [FPG]: 6.1 to 6.9 mmol/L) and the 2003 American Diabetes Association (ADA) guideline criteria (FPG: 5.6–6.9 mmol/L) [13]. Moreover, the glycosylated hemoglobin (HbA1c) of 5.7–6.4% and 6.0–6.4% also have been considered as definitions for prediabetes by ADA and National Institute for Health and Care Excellence (NICE) respectively [14,15]. Despite of the varying definitions, prediabetes represents a significant public health concern due to its high prevalence and increased risk of progression to type 2 diabetes, and the risk of multiple complications, such as cardiovascular diseases and cancers [16].

Similar to diabetes, prediabetes may be linked to carcinogenesis via mechanisms such as hyperinsulinemia, insulin resistance, chronic inflammation, and alterations in insulin-like growth factor signaling pathways [11,17]. However, the association between prediabetes and cancer may be site-specific [18]. In view of the inconsistent results reported in previous studies evaluating the role of prediabetes in the development of pancreatic cancer [19–27], we conducted a meta-analysis of longitudinal observational studies to evaluate the association between prediabetes and the risk of pancreatic cancer. By synthesizing the available evidence, we aimed to provide a comprehensive assessment of this relationship and elucidate the potential role of prediabetes as a risk factor for pancreatic cancer.

## Methods

The authors adhered to the guidelines outlined in PRISMA 2020 [28,29] and the Cochrane Handbook for Systematic Reviews and Meta-analyses [30] throughout this meta-analysis, encompassing study design, data collection, statistical analysis, and interpretation of results.

### Literature search

To identify studies relevant to the aim of the meta-analysis, we searched Medline, Embase, and Web of Science utilizing the following comprehensive search terms: ("prediabetes" OR "pre-diabetes" OR "prediabetic" OR "pre-diabetic" OR "prediabetic state" OR "borderline diabetes" OR "impaired fasting glucose" OR "impaired glucose tolerance" OR "IFG" OR "IGT") AND ("pancreatic" OR "pancreas") AND ("neoplasms" OR "carcinoma" OR "cancer" OR "tumor" OR "malignancy" OR "adenoma" OR "adenocarcinoma"). The search was restricted to research involving human subjects. The full search strategy for each database is provided in **S1 File**. We only included studies that had been published as complete articles in English in peer-reviewed journals. Additionally, we manually examined the references of relevant original and review articles for potentially pertinent studies. The literature published from database inception until March 26, 2024 was reviewed.

### Inclusion and exclusion criteria

The inclusion criteria for the potential studies were: (1) longitudinal observational studies published as full-length articles, including the prospective and retrospective cohort studies, nested case-control (NCC) studies, and post-hoc analysis of clinical trials; (2) studies evaluating a general population without pancreatic cancer at baseline; (3) prediabetes evaluated at baseline, diagnosed according to the criteria in the original studies; and (4) reporting the incidence of pancreatic cancer compared between participants with prediabetes versus those with normoglycemia during follow-up. Exclusion criteria were: (1) cross-sectional studies or case-control studies; (2) studies including patients who were diagnosed with specific diseases rather than the general population; (3) studies not evaluating prediabetes at baseline; (4) studies not reporting the incidence of pancreatic cancer during follow-up; or (5) preclinical studies, reviews, editorials, or previous meta-analyses. If studies with overlapping population were retrieved, the one with the largest sample size was included in the meta-analysis.

### Study quality evaluation and data extraction

The literature search, study identification, study quality assessment, and data collection were carried out independently by two authors. In case of disagreement, the corresponding author was consulted to reach a resolution. To evaluate the quality of the included studies, we utilized the Newcastle-Ottawa Scale (NOS) [31], which assesses three aspects: selection of the population, control of confounders, and outcome measurement and analysis. The NOS scores ranged from 1 to 9 with 9 indicating superior quality. We extracted various data from each study for subsequent analysis including study information (author, year, country, and design), participant characteristics (sample size, age, and sex), baseline prediabetes diagnosis (along with definition and number of participants diagnosed), mean follow-up duration, and methods for the validation of patients who developed pancreatic cancer during follow-up. Furthermore, variables adjusted when reporting the association between prediabetes and pancreatic cancer was also included in the extraction process. Missing study or patient characteristics were labeled as "not reported" (NR) in the data extraction table, and studies without available outcome data were omitted from the meta-analysis.

### Statistics

The relationship between prediabetes and pancreatic cancer incidence was assessed using risk ratio (RR) and corresponding 95% confidence interval (CI), comparing individuals with prediabetes to those with normoglycemia. For studies that reported HR, we directly extracted RR for comparison. For studies that reported OR, data were converted to RR for the meta-analysis according to the following equation: RR = OR/([1−pRef]+[pRef×OR], where pRef is the prevalence of the outcome in the reference group (participants with normoglycemia at baseline) [32]. The RR data and its standard error were computed based on 95% CIs or *p*-values, followed by logarithmic transformation for variance stabilization. Heterogeneity among studies was evaluated using the Cochrane Q test and I^2^ statistics [33], where an I^2^ >50% indicated significant statistical heterogeneity. The findings were combined utilizing a random-effects model that accounted for heterogeneity’s influence [30]. Sensitivity analyses involving the exclusion of one dataset at a time were conducted to assess the robustness of the results. Predefined subgroup analyses were also carried out to examine how study characteristics influenced the outcome, with median values of continuous variables used as cutoffs for defining subgroups. Publication bias in the meta-analysis was assessed through construction of funnel plots along with visual inspection for plot symmetry [34]; additionally, an Egger’s regression test was performed [34]. Statistical analysis utilized RevMan (Version 5.1; Cochrane Collaboration, Oxford, UK) and Stata software (version 12.0; Stata Corporation, College Station, TX).

## Results

### Study inclusion

**Fig 1:**
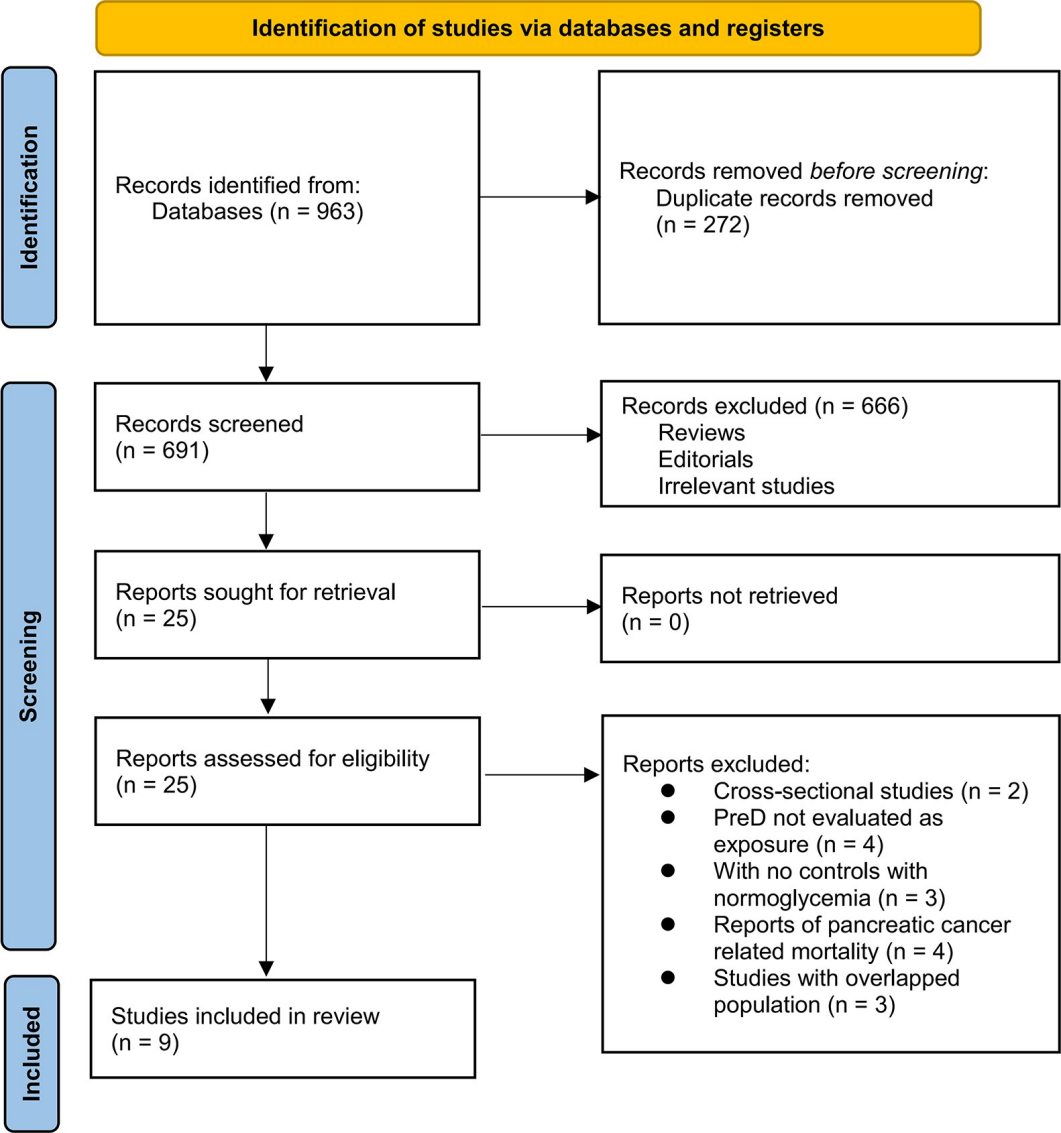
Flow diagram of database search and study inclusion.

The process of study inclusion is presented in **Fig 1**. In brief, 963 potentially relevant records were obtained after a comprehensive search of the three databases, and 272 of them were excluded due to duplication. Subsequently, a screening via titles and abstracts of the remained records further excluded 666 studies, mostly because they were unrelated to the aim of the meta-analysis. Accordingly, the full texts of the 25 remaining records were read by two independent authors, and 16 of them were further removed for the reasons listed in **Fig 1**. All studies identified after excluding duplications and reasons for exclusion of each study is shown in **S1 Table**. Finally, nine observational studies were considered to be suitable for the subsequent quantitative analyses [19–27].

### Overview of study characteristics

All data extracted in primary studies is shown in **S2 Table**. **Table 1** presents the summarized characteristics of the included studies. Overall, four prospective cohorts [19,20,22,24], two retrospective cohorts [25,27], and three NCC studies [21,23,26] were eligible for the quantitative evaluation in the meta-analysis. These studies were reported from 2005 to 2024, and were conducted in Korea, Austria, a collaboration of ten European countries, China, Sweden, and the United Kingdom. Overall, 26,444,624 subjects were included in the meta-analysis. The mean ages of the participants were 43.0 to 72.1 years, and the proportions of men were 34.3 to 71.4%. Prediabetes was defined by IFG in five studies [19,20,22,23,27], by IGT in one study [24], and by mildly elevated HbA1c in another three studies [21,25,26]. Among them, 2,052,986 (7.8%) participants had prediabetes at baseline, and they were followed for a mean duration of 3.0 to 11.6 years. The validation of pancreatic cancer incidence was mostly via local or national cancer registry or ICD codes of the healthcare databases, except for one study, in which it was validated by pathological reports [23]. Multivariate analysis was performed in all of the included studies when the association between prediabetes and risk of pancreatic cancer was evaluated with adjustment at least for age and sex. Completed risk of bias and quality certainty assessments are shown in **S3 Table**. The NOS of the included studies were six to eight stars, suggesting overall moderate to good study quality (**Table 2**).

**Table 1:** Summary of study characteristics.

Study	Location	Study design	Participants characteristics	No. of overall subjects	Mean age (years)	Men (%)	Definition of PreD	No. of participants with PreD	Mean follow-up (years)	Methods for PanC validation	Variables adjusted
Jee 2005 [19]	Korea	PC	General population	1,298,385	46.9	63.9	IFG	80,598	10	National cancer registry	Age and sex
Rapp 2006 [20]	Austria	PC	General population	140,813	43	45.2	IFG	6828	8.4	Local cancer registry	Age, sex, smoking, occupation, and BMI
Grote 2011 [21]	10 European countries	NCC	General population	932	58	48.3	HbA1c (6.0–6.4%)	154	5.3	Local cancer registry	Age, sex, smoking, alcohol drinking, and BMI
Koo 2019 [22]	Korea	PC	General population	22,801,697	49.9	50.8	IFG	1,814,242	5.5	National cancer registry	Age, sex, smoking, alcohol drinking, exercise, and BMI
Ke 2021 [24]	China	PC	General population	9224	52.3	34.3	IGT	1454	7.5	Local cancer registry	Age and sex
Jacobson 2021 [23]	Sweden	NCC	General population	899	64.2	61	IFG	113	7	Pathologically validated	Age, sex, BMI and smoking
McDonnell 2023 [25]	UK	RC	General population	473,264	56.4	44.7	HbA1c (6.0–6.4%)	15,852	11.6	ICD codes	Age, sex, BMI, weight change, smoking status, alcohol consumption, processed meat intake, and ethnic background
Tan 2023 [26]	UK	NCC	General population	289,356	72.1	49.7	HbA1c (6.0–6.4%)	12,839	3	ICD codes	Age, sex, ethnicity, deprivation quintile, BMI, smoking, and alcohol consumption
Ahn 2024 [27]	Korea	RC	General population	1,430,054	49.8	71.4	IFG	120,906	6.4	National cancer registry	Age, sex, BMI, smoking status, alcohol consumption, physical activity, hypertension, and dyslipidemia

PreD, prediabetes; PanC, pancreatic cancer; PC, prospective cohort; RC, retrospective cohort; NCC, nested case-control; IFG, impaired fasting glucose; IGT, impaired glucose tolerance; HbA1c, hemoglobin A1c; ICD, International Classification of Diseases; BMI, body mass index.

**Table 2:** Study quality assessment details according to the Newcastle-Ottawa scale.

Study	Representativeness of the exposed cohort	Selection of the non-exposed cohort	Ascertainment of exposure	Outcome not present at baseline	Control for age and sex	Control for other confounding factors	Assessment of outcome	Enough long follow-up duration	Adequacy of follow-up of cohorts	Total
Jee 2005 [19]	1	1	1	1	1	0	0	1	1	7
Rapp 2006 [20]	1	1	1	1	1	1	0	1	1	8
Grote 2011 [21]	0	1	1	1	1	1	0	1	1	7
Koo 2019 [22]	1	1	1	1	1	1	0	1	1	8
Ke 2021 [24]	1	1	1	1	1	0	0	1	1	7
Jacobson 2021 [23]	0	1	1	1	1	1	1	1	1	8
McDonnell 2023 [25]	0	1	1	1	1	1	0	1	1	7
Tan 2023 [26]	0	1	1	1	1	1	0	0	1	6
Ahn 2024 [27]	0	1	1	1	1	1	0	1	1	7

### Results of meta-analysis and sensitivity analysis

**Fig 2:**
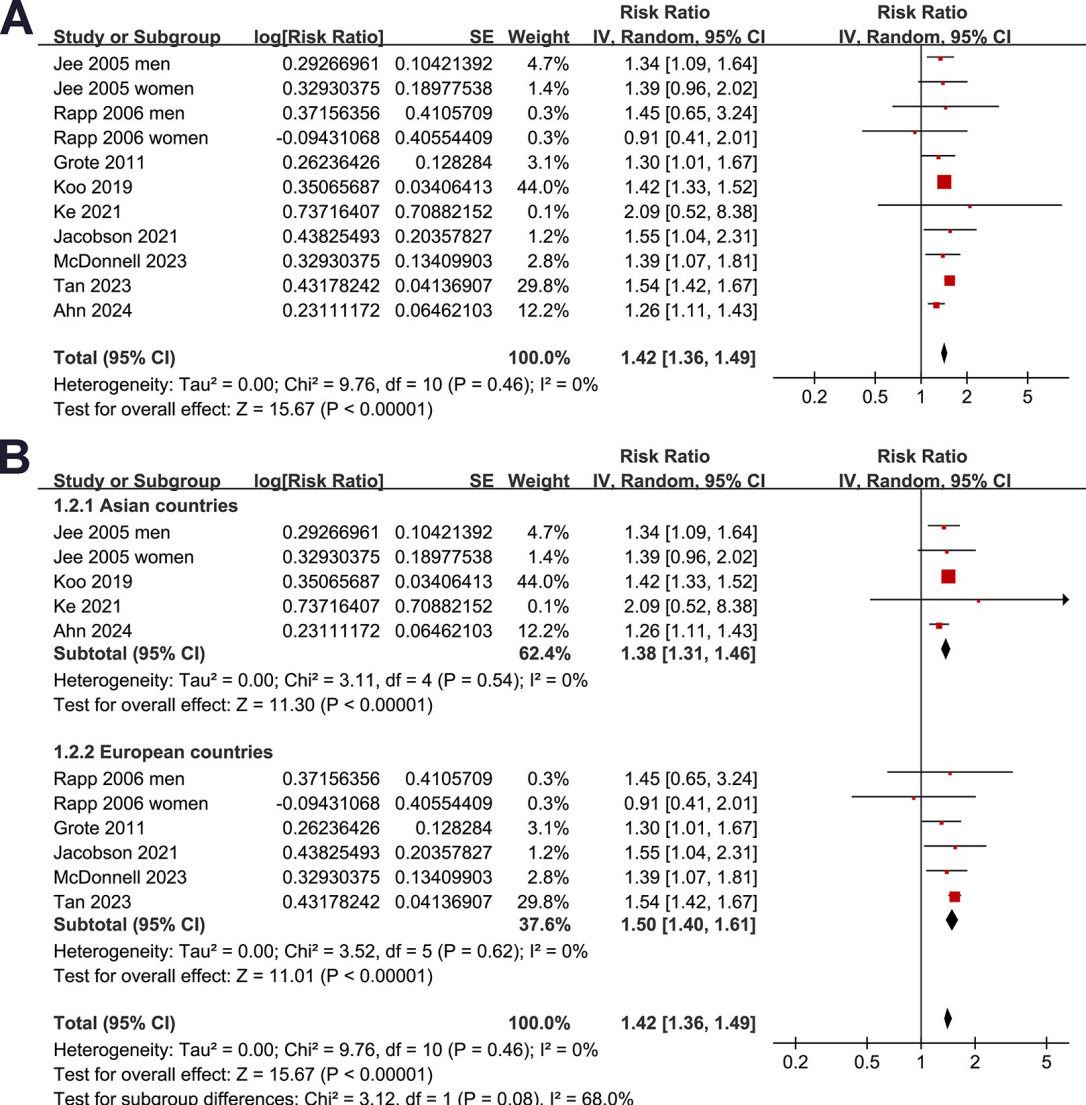
Forest plots representing the results of the meta-analysis: A, plots for the overall meta-analysis regarding the association between prediabetes and pancreatic cancer incidence; and B, plots for the subgroup analysis according to study location.

Since two studies reported the outcome in men and women separately [19,20], these datasets were independently included, which made 11 datasets from nine studies [19–27] available for meta-analysis. The pooled results showed that, compared to people with normoglycemia, those with prediabetes at baseline had a higher incidence of developing pancreatic cancer during follow-up (RR: 1.42, 95% CI: 1.36 to 1.49, *p*<0.001; **Fig 2A**) with no statistical heterogeneity (I^2^ = 0%). Sensitivity analysis by excluding one dataset at a time did not significantly change the results (RR: 1.38 to 1.45, *p* all <0.05). Since two studies (one prospective and one retrospective) [22,27] were both from the Korean National Health Insurance database, to assess for the risk of selection bias, a sensitivity analysis excluding either of these two studies was performed, which also showed consistent results (Excluding the study by Koo et al, RR: 1.41 [1.32, 1.51], excluding the study by Ahn et al, RR: 1.45 [1.38, 1.52]).

### Results of the subgroup analyses

**Fig 3:**
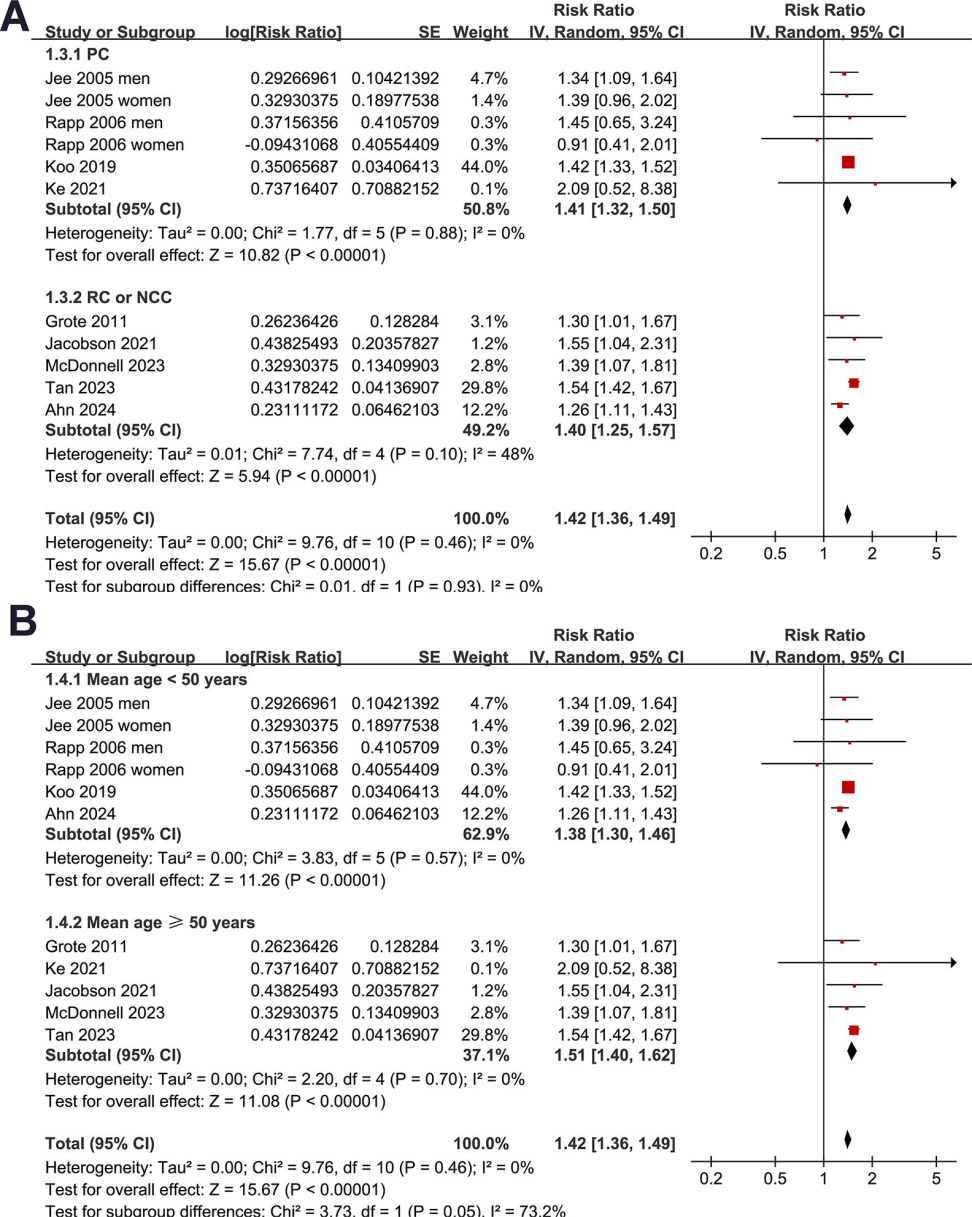
Forest plots for representing the results of the subgroup analysis: A, subgroup analysis according to study design; and B, subgroup analysis according to mean age of the participants.

**Fig 4:**
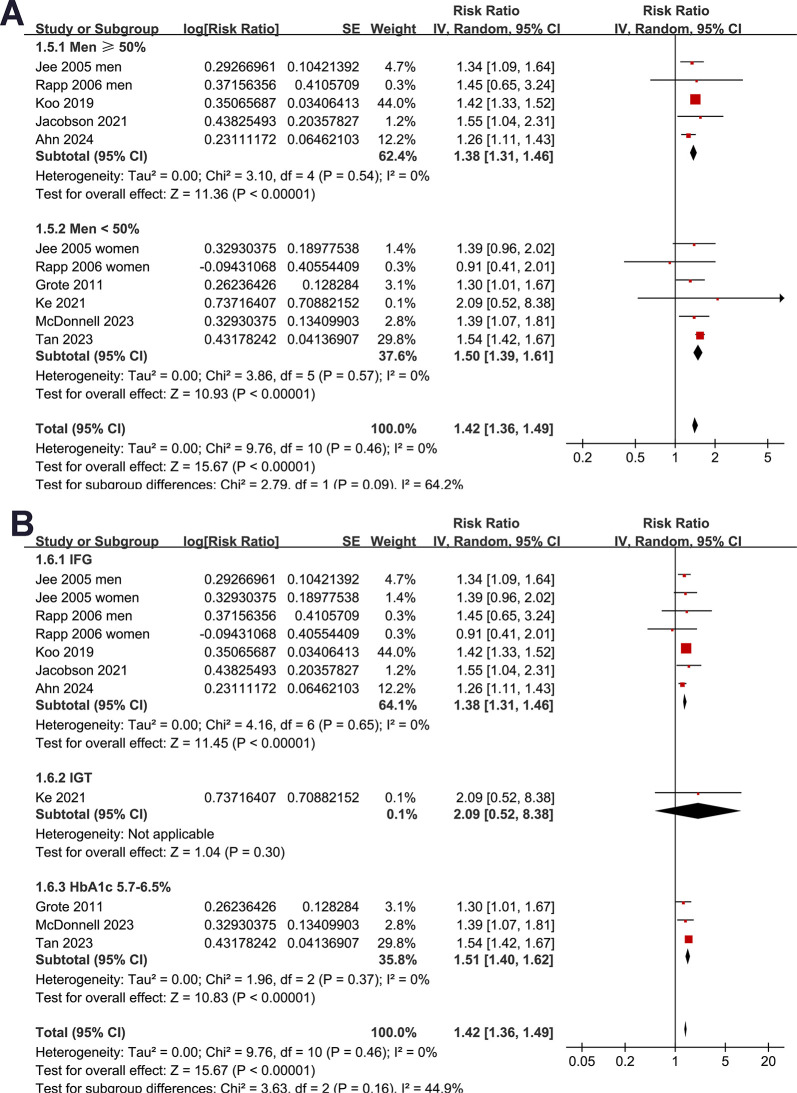
Forest plots for representing the results of the subgroup analysis: A, subgroup analysis according to proportions of men; and B, subgroup analysis according to the definition of prediabetes.

**Fig 5:**
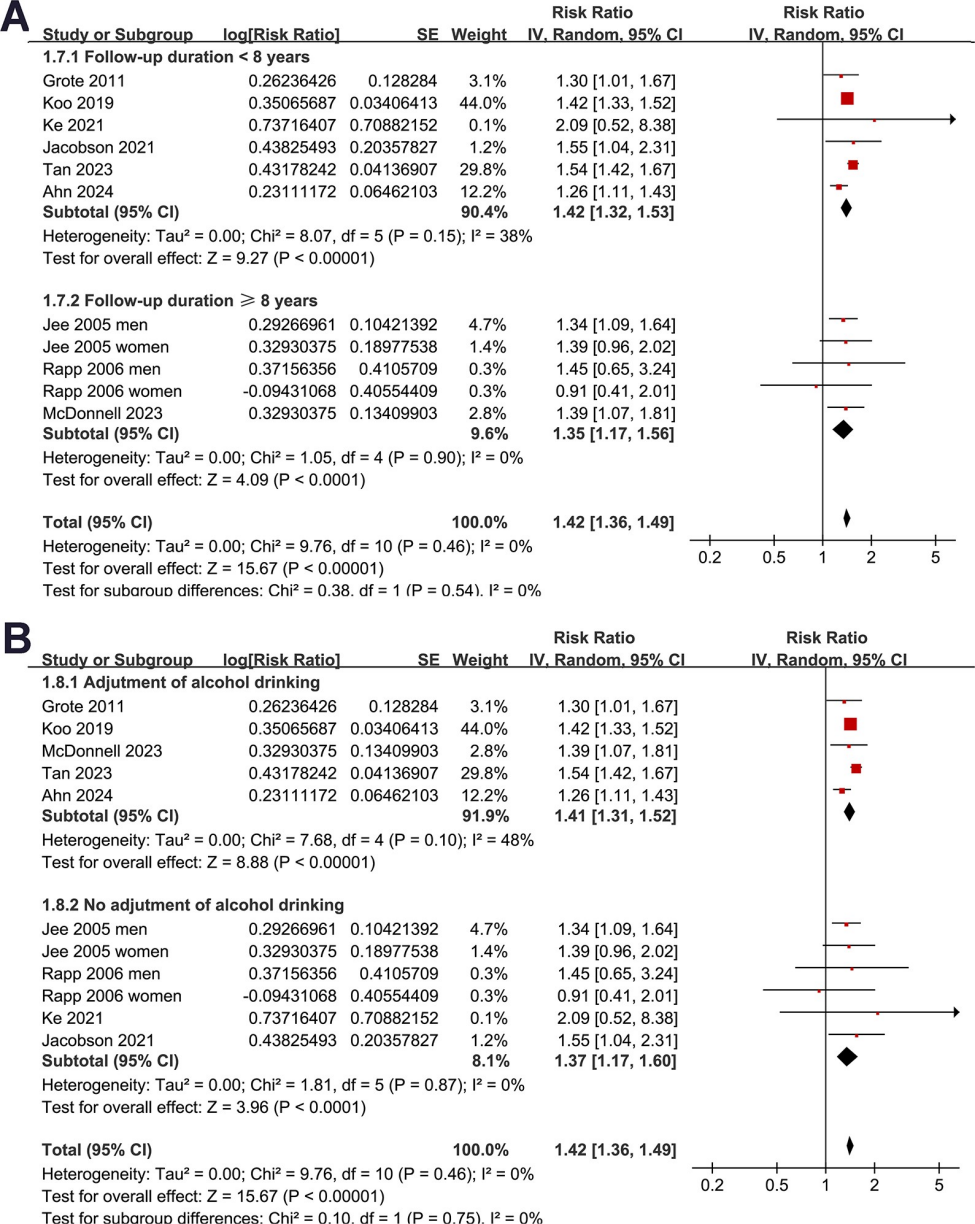
Forest plots for representing the results of the subgroup analysis: A, subgroup analysis according to the mean follow-up duration; and B, subgroup analysis according to the adjustment for alcohol intake.

Subgroup analyses indicated that the association between prediabetes and increased risk of pancreatic cancer was consistent in studies from Asian and European countries (RR: 1.38 [1.31, 1.46] versus 1.50 [1.40, 1.61], *p* for subgroup difference = 0.08; **Fig 2B**), in prospective and retrospective/NCC studies (RR: 1.41 [1.32, 1.50] versus 1.40 [1.25, 1.57], *p* for subgroup difference = 0.93; **Fig 3A**), in participants of mean age < and ≥50 years (RR: 1.38 [1.30, 1.46] versus 1.51 [1.40, 1.62], *p* for subgroup difference = 0.05, **Fig 3B**), and in studies with men ≥ and <50% (RR: 1.38 [1.31, 1.46] versus 1.50 [1.39, 1.61], *p* for subgroup difference = 0.09; **Fig 4A**). The association between prediabetes and the increased incidence of pancreatic cancer was significant in studies with prediabetes defined by IFG (RR: 1.38 [1.31, 1.46], *p*<0.001) and mildly elevated HbA1c (RR: 1.51 [1.40, 1.62], *p*<0.001), but not in studies with predicates defined by IGT (RR: 2.09 [0.52, 8.38], *p* = 0.30), although the subgroup difference was not statistically significant (*p* for subgroup difference = 0.16; **Fig 4B**). Further subgroup analysis showed similar results in studies of follow-up duration < and ≥8 years (RR: 1.42 [1.32, 1.53] versus 1.35 [1.17, 1.56], *p* for subgroup difference = 0.54; **Fig 5A**), and in studies with or without adjustment of alcohol drinking (RR: 1.41 [1.31, 1.52] versus 1.37 [1.17, 1.60], *p* for subgroup difference = 0.75; **Fig 5B**).

### Publication bias

**Fig 6:**
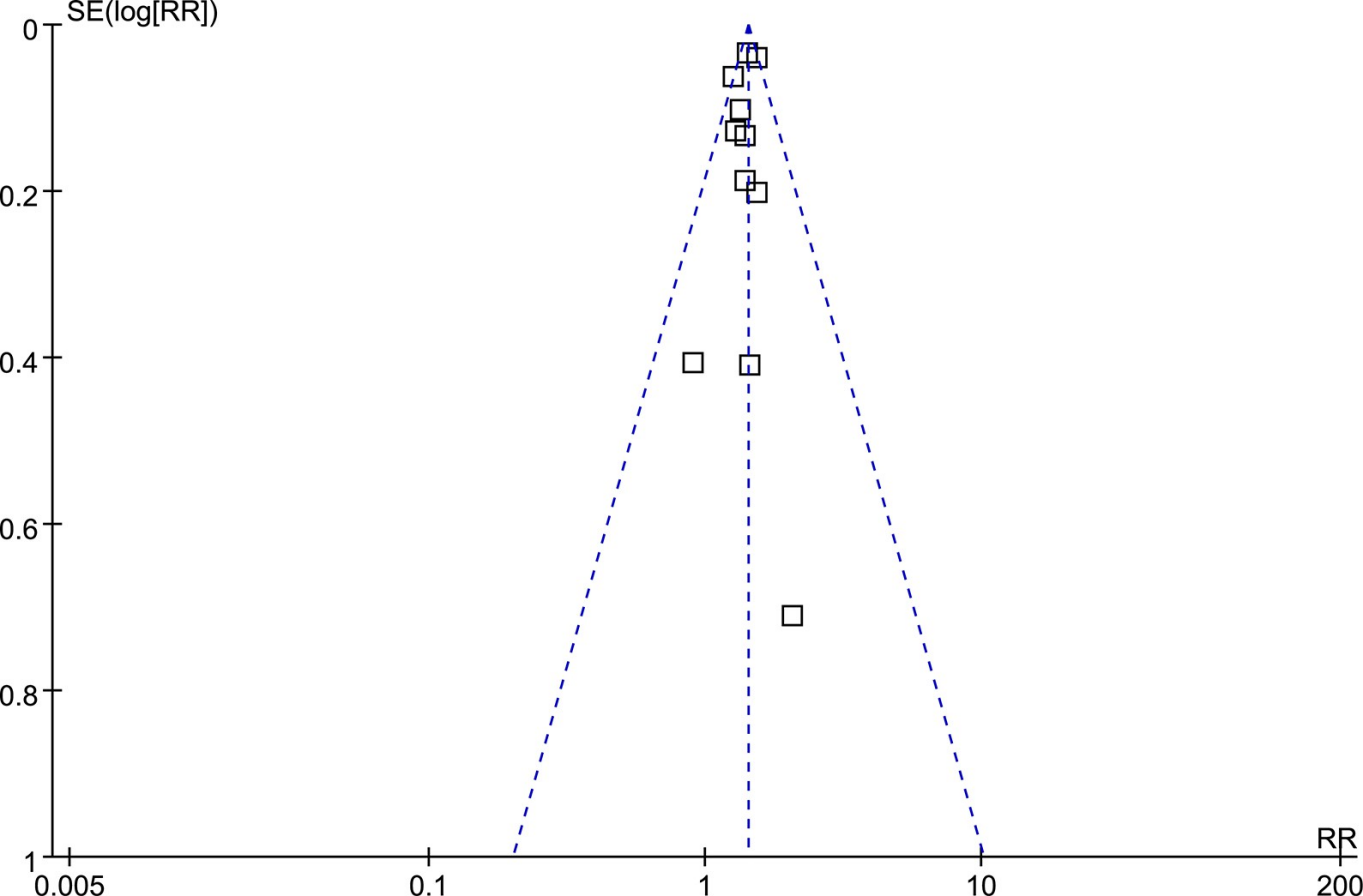
Funnel plots for the meta-analysis of the association between prediabetes and pancreatic cancer incidence.

Upon visual inspection, the funnel plots for meta-analysis of the association between prediabetes and pancreatic cancer risk appear symmetrical, indicating a low likelihood of publication bias (**Fig 6**). Additionally, Egger’s regression test results (*p* = 0.45) also support this conclusion by suggesting a low risk of publication bias.

## Discussion

Our meta-analysis provides up-to-date evidence indicating that prediabetes is associated with an increased risk of pancreatic cancer. The robustness and stability of the finding were further proved by the consistent results in sensitivity and subgroup analyses. This finding has important clinical and public health implications, as prediabetes affects a large proportion of the population and represents a significant risk factor for the development of type 2 diabetes. Understanding the association between prediabetes and pancreatic cancer risk is essential for the development of effective prevention and early detection strategies.

To the best of our knowledge, only one meta-analysis has been performed to evaluate the association between prediabetes and cancer risk [18]. This meta-analysis included studies conducted before 2014 and concluded that prediabetes was related to an increased overall cancer risk, and a subgroup analysis including three studies suggested a possible association between prediabetes and pancreatic cancer [18]. However, besides including studies reporting pancreatic cancer incidence, two studies reporting pancreatic cancer mortality were also included, which may have confounded the results [18]. This concern arises because cancer mortality is a difference outcome from cancer incidence, which may also be influenced by factors such as anticancer treatments. Moreover, the limited number of available studies prevented further investigation of the potential study characteristics related to the association between prediabetes and pancreatic cancer [18]. The current meta-analysis, which included 11 datasets, provides further evidence that prediabetes may increase the incidence of pancreatic cancer. In addition, all of the included studies reported multivariate analyses, which suggests that the association between prediabetes and pancreatic cancer risk was not confounded by study characteristics such as age and sex. In addition, subgroup analysis showed similar results in studies with adjustment for alcohol intake, a recognized risk factor of pancreatic cancer [35]. These findings indicate that continuous hyperglycemia is a potential risk factor for pancreatic cancer, even below the threshold for the diagnosis of diabetes.

The underlying mechanisms linking prediabetes to pancreatic cancer risk are not fully understood but may involve several pathways. Hyperinsulinemia and insulin resistance, hallmark features of prediabetes, have been proposed as potential mediators of this association [36]. Insulin has mitogenic effects and can promote cell proliferation and inhibit apoptosis, which may contribute to the development and progression of pancreatic cancer [37]. Chronic inflammation, another feature of prediabetes, has also been implicated in pancreatic carcinogenesis [38,39]. Additionally, alterations in insulin-like growth factor signaling pathways may play a role in the development of pancreatic cancer in individuals with prediabetes [40].

Emerging evidence suggests a temporal relationship between the onset of hyperglycemia, progressing to overt diabetes, and the development of pancreatic ductal adenocarcinoma (PDAC) [41]. Studies have shown that new-onset diabetes, particularly within three years prior to the diagnosis of PDAC and in older patients, may be an early manifestation of the malignancy rather than a pre-existing condition [42,43]. This observation highlights the potential role of hyperglycemia as an early warning sign of PDAC. The underlying mechanisms remain unclear, but it is hypothesized that tumor-induced alterations in pancreatic islet cells contribute to impaired glucose metabolism, which precedes the clinical presentation of PDAC [44]. Glycemic dysregulation in the context of pancreatic neoplasia is a multifactorial process. PDAC can disrupt normal pancreatic function through direct destruction of insulin-producing β-cells, leading to impaired insulin secretion [45]. Additionally, PDAC-associated systemic inflammation and tumor-derived factors, such as adrenomedullin [46] and islet amyloid polypeptide [47], can induce insulin resistance, further contributing to hyperglycemia. These alterations not only exacerbate the metabolic challenges faced by patients but also complicate the clinical management of PDAC. Following surgical resection of PDAC, the dynamics of glycemic control can vary significantly among patients. While some may experience an improvement in glycemic status due to the removal of the tumor burden [48], others may develop new or worsening diabetes, particularly if a significant portion of the pancreatic tissue is resected [49]. The extent of resection, along with pre-existing metabolic conditions, plays a critical role in determining post-operative glycemic outcomes. Long-term management of glycemic control remains a challenge in this population, necessitating close monitoring and individualized treatment strategies.

Although studies are needed to clarify the key molecular mechanisms underlying the connection between prediabetes and pancreatic cancer risk, our findings highlight the importance of early identification and intervention for individuals with prediabetes to potentially reduce their risk of developing pancreatic cancer. Lifestyle modifications, including dietary changes, increased physical activity, and weight loss, have been shown to prevent or delay the progression from prediabetes to diabetes [50]. These interventions may also help reduce the risk of pancreatic cancer in this population. Furthermore, our meta-analysis reveals a significant association between prediabetes and an increased risk of pancreatic cancer, suggesting that individuals with prediabetes may represent a high-risk group warranting closer surveillance. This finding highlights the potential for integrating glucose tolerance status into pancreatic cancer screening strategies. Given the relatively low incidence of pancreatic cancer, targeted screening for those with prediabetes, particularly those with additional risk factors, could enhance early detection efforts. Further research is needed to refine screening protocols and assess the clinical implications and cost-effectiveness of incorporating prediabetes into risk assessment models. Future research is needed to further elucidate the underlying mechanisms linking prediabetes to pancreatic cancer risk. Longitudinal studies investigating the temporal relationship between prediabetes, diabetes, and pancreatic cancer are warranted to better understand the natural history of these conditions and identify opportunities for intervention. Additionally, studies exploring potential biomarkers for the early detection of pancreatic cancer in individuals with prediabetes are needed.

One of the strengths of our meta-analysis is the large sample size, which increases the statistical power to detect an association between prediabetes and pancreatic cancer risk. Additionally, we utilized a random-effects model to account for between-study heterogeneity and conducted sensitivity analyses to assess the robustness of our findings. Finally, the reliability of the finding was validated by multiple sensitivity and subgroup analyses. However, our meta-analysis also has several limitations. First, the included studies were observational in nature, which precludes establishing causality between prediabetes and pancreatic cancer risk. Second, the definition of prediabetes varied across studies, which may have introduced heterogeneity into our analysis. Third, we cannot rule out the possibility of residual confounding, despite attempts to control for potential confounders in the individual studies. Finally, it remains unknown whether the association between prediabetes and risk of pancreatic cancer was consistent in different pathological types of cancer.

## Conclusions

In conclusion, our meta-analysis provides evidence that prediabetes is associated with an increased risk of pancreatic cancer. Further research is needed to better understand the underlying mechanisms driving this association and to develop targeted interventions aimed at reducing the risk of pancreatic cancer in individuals with prediabetes. Nevertheless, our findings underscore the importance of early identification and intervention for prediabetes as a potential strategy for pancreatic cancer prevention.

## Supporting information

S1 Checklist PRISMA_2020_checklist. (DOCX)

S1 File Full search strategy. (DOCX)

S1 Table Studies identified after excluding duplications. (DOC)

S2 Table All data extracted in primary studies. (XLSX)

S3 Table Completed risk of bias and quality certainty assessments. (XLSX)
