# Supplementary material for: Prediabetes increases the risk of pancreatic cancer: A meta-analysis of longitudinal observational studies
Source: PLoS One. 2024 Oct 15;19(10):e0311911. doi: 10.1371/journal.pone.0311911 (PMC11478827; doi:10.1371/journal.pone.0311911)
Supplement: S1 File — (DOCX) [file pone.0311911.s002.docx]

**Full search strategy for each database**

**Medline**

("prediabetes"[MeSH Terms] OR "pre-diabetes"[All Fields] OR "prediabetic"[All Fields] OR "pre-diabetic"[All Fields] OR "prediabetic state"[All Fields] OR "borderline diabetes"[All Fields] OR "impaired fasting glucose"[All Fields] OR "impaired glucose tolerance"[All Fields] OR "IFG"[All Fields] OR "IGT"[All Fields])

AND ("pancreatic"[All Fields] OR "pancreas"[All Fields] OR "Pancreatic Neoplasms"[MeSH Terms]) AND ("neoplasms"[All Fields] OR "carcinoma"[All Fields] OR "cancer"[All Fields] OR "tumor"[All Fields] OR "malignancy"[All Fields] OR "adenoma"[All Fields] OR "adenocarcinoma"[All Fields] OR "Neoplasms"[MeSH Terms] OR "Carcinoma"[MeSH Terms] OR "Cancer"[MeSH Terms])

**Embase**

('prediabetes'/exp OR 'prediabetes' OR 'pre-diabetes' OR 'prediabetic' OR 'pre-diabetic' OR 'prediabetic state' OR 'borderline diabetes' OR 'impaired fasting glucose' OR 'impaired glucose tolerance' OR 'IFG' OR 'IGT') AND ('pancreatic' OR 'pancreas') AND ('neoplasm'/exp OR 'neoplasms' OR 'carcinoma' OR 'cancer' OR 'tumor' OR 'malignancy' OR 'adenoma' OR 'adenocarcinoma') AND [humans]/lim AND [clinical study]/lim AND [embase]/lim

**Web of Science**

TS=("prediabetes" OR "pre-diabetes" OR "prediabetic" OR "pre-diabetic" OR "prediabetic state" OR "borderline diabetes" OR "impaired fasting glucose" OR "impaired glucose tolerance" OR "IFG" OR "IGT") AND TS=("pancreatic" OR "pancreas") AND TS=("neoplasms" OR "carcinoma" OR "cancer" OR "tumor" OR "malignancy" OR "adenoma" OR "adenocarcinoma")
