# Supplementary material for: Prediabetes increases the risk of pancreatic cancer: A meta-analysis of longitudinal observational studies
Source: PLoS One. 2024 Oct 15;19(10):e0311911. doi: 10.1371/journal.pone.0311911 (PMC11478827; doi:10.1371/journal.pone.0311911)
Supplement: S1 Table — (DOC) [file pone.0311911.s003.doc]

**S1 Table. Studies identified after excluding duplications (n = 691)**

| **Studies excluded in title and abstract screening with reasons (n = 666)** | |
| --- | --- |
| Reasons | Citations |
| Reviews (n = 157) | 1. Sensi S. Some aspects of circadian variations of carbohydrate metabolism and related hormones in man. Chronobiologia. 1974;1(4):396-404.  2. Stogdale L. Definition of diabetes mellitus. The Cornell veterinarian. 1986;76(2):156-74.  3. McLaughlin S. Pancreatic cancer and diabetes. The Diabetes educator. 1994;20(1):20, 4, 6.  4. Cooper GJS, Tse CA. Amylin, amyloid and age-related disease. Drugs and Aging. 1996;9(3):202-12.  5. Hill DJ. Insulin secretion: Lessons from tissue culture. Diabetes Reviews. 1996;4(4):390-402.  6. Pour PM. The role of Langerhans islets in pancreatic ductal adenocarcinoma. Frontiers in bioscience : a journal and virtual library. 1997;2((Pour P.M.) UNMC/Eppley Cancer Center and Department of Pathology and Microbiology, University of Nebraska Medoical Center, 600 South 42 nd Street, Omaha, NE 68198-6805, USA.):d271-82.  7. Hayashi T, Faustman D. The role of the proteasome in autoimmunity. Diabetes/Metabolism Research and Reviews. 2000;16(5):325-37.  8. Tomassetti P, Migliori M, Lalli S, Campana D, Tomassetti V, Corinaldesi R. Epidemiology, clinical features and diagnosis of gastroenteropancreatic endocrine tumours. Annals of Oncology. 2001;12(SUPPLE. 2):S95-S9.  9. Goldstein BJ. Insulin resistance as the core defect in type 2 diabetes mellitus. American Journal of Cardiology. 2002;90(5 SUPPL.):3G-10G.  10. Tobin BW, Uchakin PN, Leeper-Woodford SK. Insulin secretion and sensitivity in space flight: Diabetogenic effects. Nutrition. 2002;18(10):842-8.  11. Corry DB, Tuck ML. The effect of aldosterone on glucose metabolism. Current Hypertension Reports. 2003;5(2):106-9.  12. Saruc M, Pour PM. Diabetes and its relationship to pancreatic carcinoma. Pancreas. 2003;26(4):381-7.  13. Christen U, Juedes A, Homann D, Von Herrath MG. Virally induced inflammation and therapeutic avenues in type 1 diabetes. Endocrinology and Metabolism Clinics of North America. 2004;33(1):45-58.  14. Kennedy RL, Chokkalingham K, Srinivasan R. Obesity in the elderly: Who should we be treating, and why, and how? Current Opinion in Clinical Nutrition and Metabolic Care. 2004;7(1):3-8.  15. Öberg K, Kvols L, Caplin M, Dell Fave G, de Herder W, Rindi G, et al. Consensus report on the use of somatostatin analogs for the management of neuroendocrine tumors of the gastroenteropancreatic system. Annals of Oncology. 2004;15(6):966-73.  16. Ristow M. Neurodegenetive disorders associated with diabetes mellitus. Journal of Molecular Medicine. 2004;82(8):510-29.  17. Andersen G, Hansen T, Pedersen O. Genetics of common forms of glycaemia with pathological impact on vascular biology: Are we on the right tract? Current Molecular Medicine. 2005;5(3):261-74.  18. Mizushige K. Antioxidative activities of thiazolidinediones and dihydropyridine-type calcium antagonists. IRYO - Japanese Journal of National Medical Services. 2005;59(11):581-92.  19. Taiwo BO. Insulin resistance, HIV infection, and anti-HIV therapies. AIDS Reader. 2005;15(4):171-6+9-80.  20. van der Hoek J, Hofland LJ, Lamberts SWJ. Novel subtype specific and universal somatostatin analogues: Clinical potential and pitfalls. Current Pharmaceutical Design. 2005;11(12):1573-92.  21. Yalniz M, Pour PM. Diabetes mellitus: a risk factor for pancreatic cancer? Langenbeck's archives of surgery / Deutsche Gesellschaft für Chirurgie. 2005;390(1):66-72.  22. Emamaullee JA, Shapiro AMJ. Interventional strategies to prevent β-cell apoptosis in islet transplantation. Diabetes. 2006;55(7):1907-14.  23. Nanji SA, Shapiro AMJ. Advances in pancreatic islet transplantation in humans. Diabetes, Obesity and Metabolism. 2006;8(1):15-25.  24. Reue K, Phan J. Metabolic consequences of lipodystrophy in mouse models. Current Opinion in Clinical Nutrition and Metabolic Care. 2006;9(4):436-41.  25. Davies SL, Moral MÀ, Bozzo J. Salicylates targeting insulin resistance. Drugs of the Future. 2007;32(4):361-5.  26. Dedoussis GVZ, Kaliora AC, Panagiotakos DB. Genes, diet and type 2 diabetes mellitus: A review. Review of Diabetic Studies. 2007;4(1):13-24.  27. Maassen JA. Do we inherit or acquire mitochondrial dysfunction in the metabolic syndrome and Type 2 diabetes? Expert Review of Endocrinology and Metabolism. 2007;2(5):599-606.  28. Nino-Fong R, Collins TM, Chan CB. Nutrigenomics, β-cell function and type 2 diabetes. Current Genomics. 2007;8(1):29-32.  29. O'Sullivan EP, Walsh CH. Endocrinopathy of HFE-related hemochromatosis. Expert Review of Endocrinology and Metabolism. 2007;2(2):277-86.  30. Sharma AM, Staels B. Review: Peroxisome proliferator-activated receptor γ and adipose tissue - Understanding obesity-related changes in regulation of lipid and glucose metabolism. Journal of Clinical Endocrinology and Metabolism. 2007;92(2):386-95.  31. Szalat A, Raz I. Gender-specific care of diabetes. Women's Health. 2007;3(6):735-64.  32. Ben-Ziv RG, Hod M. Gestational diabetes mellitus. Fetal and Maternal Medicine Review. 2008;19(3):245-69.  33. Cali AMG, Caprio S. Prediabetes and type 2 diabetes in youth: An emerging epidemic disease? Current Opinion in Endocrinology, Diabetes and Obesity. 2008;15(2):123-7.  34. Dossus L, Kaaks R. Nutrition, metabolic factors and cancer risk. Best Practice and Research: Clinical Endocrinology and Metabolism. 2008;22(4):551-71.  35. Małodobra M, Dobosz T. Genetic basics of insulin resistance and its role in type 2 diabetes pathogenesis. Diabetologia Doswiadczalna i Kliniczna. 2008;8(3):95-103.  36. McTiernan A. Mechanisms linking physical activity with cancer. Nature Reviews Cancer. 2008;8(3):205-11.  37. Qi L, Hu FB, Hu G. Genes, environment, and interactions in prevention of type 2 diabetes: A focus on physical activity and lifestyle changes. Current Molecular Medicine. 2008;8(6):519-32.  38. Jessani S, Millane T, Lip GYH. Vascular damage in impaired glucose tolerance: An unappreciated phenomenon? Current Pharmaceutical Design. 2009;15(29):3417-32.  39. Kobayashi T, Tanaka S, Aida K, Takizawa S, Shimura H, Endo T. Diabetes associated with autoimmune pancreatitis: New insights into the mechanism of β-cell dysfunction. Expert Review of Endocrinology and Metabolism. 2009;4(6):591-602.  40. Naik RG, Brooks-Worrell BM, Palmer JP. Latent autoimmune diabetes in adults. Journal of Clinical Endocrinology and Metabolism. 2009;94(12):4635-44.  41. Resmini E, Minuto F, Colao A, Ferone D. Secondary diabetes associated with principal endocrinopathies: The impact of new treatment modalities. Acta Diabetologica. 2009;46(2):85-95.  42. Staiger H, Machicao F, Fritsche A, Häring HU. Pathomechanisms of type 2 diabetes genes. Endocrine Reviews. 2009;30(6):557-85.  43. Watson D, Loweth AC. Oxidative and nitrosative stress in β-cell apoptosis: Their contribution to β-cell loss in type 1 diabetes mellitus. British Journal of Biomedical Science. 2009;66(4):208-15.  44. Dinneen SF. What is diabetes? Medicine. 2010;38(11):589-91.  45. Golubnitschaja O. Advanced diabetes care: Three levels of prediction, prevention & personalized treatment. Current Diabetes Reviews. 2010;6(1):42-51.  46. Haller MJ, Atkinson MA, Schatz DA. Efforts to prevent and halt autoimmune beta cell destruction. Endocrinology and Metabolism Clinics of North America. 2010;39(3):527-39.  47. Khunti K, Davies M. Glycaemic goals in patients with type 2 diabetes: Current status, challenges and recent advances. Diabetes, Obesity and Metabolism. 2010;12(6):474-84.  48. Kostapanos MS, Liamis GL, Milionis HJ, Elisaf MS. Do statins beneficially or adversely affect glucose homeostasis? Current Vascular Pharmacology. 2010;8(5):612-31.  49. López-Calderero I, Chávez ES, García-Carbonero R. The insulin-like growth factor pathway as a target for cancer therapy. Clinical and Translational Oncology. 2010;12(5):326-38.  50. Milanetti F, Abinun M, Voltarelli JC, Burt RK. Autologous hematopoietic stem cell transplantation for childhood autoimmune disease. Pediatric Clinics of North America. 2010;57(1):239-71.  51. Van Raalte DH, Van Der Zijl NJ, Diamant M. Pancreatic steatosis in humans: Cause or marker of lipotoxicity? Current Opinion in Clinical Nutrition and Metabolic Care. 2010;13(4):478-85.  52. Wong FS, Hu C, Xiang Y, Wen L. To B or not to B-pathogenic and regulatory B cells in autoimmune diabetes. Current Opinion in Immunology. 2010;22(6):723-31.  53. Benito M. Tissue specificity on insulin action and resistance: Past to recent mechanisms. Acta Physiologica. 2011;201(3):297-312.  54. Chen N, Unnikrishnan I R, Anjana RM, Mohan V, Pitchumoni CS. The complex exocrine-endocrine relationship and secondary diabetes in exocrine pancreatic disorders. Journal of Clinical Gastroenterology. 2011;45(10):850-61.  55. Katz JD, Janssen EM. Breaking T cell tolerance to beta cell antigens by merocytic dendritic cells. Cellular and Molecular Life Sciences. 2011;68(17):2873-83.  56. Motoo Y, Shimasaki T, Ishigaki Y, Nakajima H, Kawakami K, Minamoto T. Metabolic disorder, inflammation, and deregulated molecular pathways converging in pancreatic cancer development: Implications for new therapeutic strategies. Cancers. 2011;3(1):446-60.  57. Orban T, Kis JT. Prevention of type 1 diabetes mellitus using a novel vaccine. Therapeutic Advances in Endocrinology and Metabolism. 2011;2(1):9-16.  58. Piątkiewicz P, Czech A. Glucose metabolism disorders and the risk of cancer. Archivum Immunologiae et Therapiae Experimentalis. 2011;59(3):215-30.  59. Verma AR, Papalois V. Evaluating steatosis in pancreatic transplant. Experimental and Clinical Transplantation. 2011;9(3):159-64.  60. Chiang JL, Haller MJ, Schatz DA. Update on Global Intervention Studies in Type 1 Diabetes. Endocrinology and Metabolism Clinics of North America. 2012;41(4):695-712.  61. Ciavattini A, Ciattaglia F, Cecchi S, Gagliardini R, Tranquilli AL. Two successful pregnancies in a woman affected by cystic fibrosis: Case report and review of the literature. Journal of Maternal-Fetal and Neonatal Medicine. 2012;25(2):113-5.  62. Gong Z, Muzumdar RH. Pancreatic function, type 2 diabetes, and metabolism in aging. International Journal of Endocrinology. 2012;2012((Gong Z., zhenwei.gong@einstein.yu.edu; Muzumdar R.H., radhika.muzumdar@einstein.yu.edu) Department of Pediatrics, Albert Einstein College of Medicine, Children's Hospital at Montefiore, Bronx, NY 10461, United States).  63. Muniraj T, Chari ST. Diabetes and pancreatic cancer. Minerva Gastroenterologica e Dietologica. 2012;58(4):331-45.  64. Rinke A, Michl P, Gress T. Medical treatment of gastroenteropancreatic neuroendocrine tumors. Cancers. 2012;4(1):113-29.  65. Wolin EM. The expanding role of somatostatin analogs in the management of neuroendocrine tumors. Gastrointestinal Cancer Research. 2012;5(5):161-8.  66. Aurora RN, Punjabi NM. Obstructive sleep apnoea and type 2 diabetes mellitus: A bidirectional association. The Lancet Respiratory Medicine. 2013;1(4):329-38.  67. Barlow AD, Nicholson ML, Herbert TP. Evidence for rapamycin toxicity in pancreatic β-Cells and a review of the underlying molecular mechanisms. Diabetes. 2013;62(8):2674-82.  68. Cheng Y, Ren X, Hait WN, Yang JM. Therapeutic targeting of autophagy in disease: Biology and pharmacology. Pharmacological Reviews. 2013;65(4):1162-97.  69. Dunmore SJ, Brown JEP. The role of adipokines in β-cell failure of type 2 diabetes. Journal of Endocrinology. 2013;216(1):T37-T45.  70. Duque-Guimarães DE, Ozanne SE. Nutritional programming of insulin resistance: Causes and consequences. Trends in Endocrinology and Metabolism. 2013;24(10):525-35.  71. Gallagher EJ, Leroith D. Diabetes, antihyperglycemic medications and cancer risk: Smoke or fire? Current Opinion in Endocrinology, Diabetes and Obesity. 2013;20(5):485-94.  72. Holst JJ, Deacon CF. Is there a place for incretin therapies in obesity and prediabetes? Trends in Endocrinology and Metabolism. 2013;24(3):145-52.  73. Matsuda M, Shimomura I. Increased oxidative stress in obesity: Implications for metabolic syndrome, diabetes, hypertension, dyslipidemia, atherosclerosis, and cancer. Obesity Research and Clinical Practice. 2013;7(5):e330-e41.  74. Mudaliar S. Choice of early treatment regimen and impact on β-cell preservation in type 2 diabetes. International Journal of Clinical Practice. 2013;67(9):876-87.  75. Park SY, Jeong HJ, Yang WM, Lee W. Implications of microRNAs in the pathogenesis of diabetes. Archives of Pharmacal Research. 2013;36(2):154-66.  76. Rayman MP, Stranges S. Epidemiology of selenium and type 2 diabetes: Can we make sense of it? Free Radical Biology and Medicine. 2013;65((Rayman M.P., m.rayman@surrey.ac.uk) Faculty of Health and Medical Sciences, University of Surrey, Guildford GU2 7XH, United Kingdom):1557-64.  77. Teo AKK, Wagers AJ, Kulkarni RN. New opportunities: Harnessing induced pluripotency for discovery in diabetes and metabolism. Cell Metabolism. 2013;18(6):775-91.  78. Xuan Y, Zhao HY, Liu JM. Vitamin D and Type 2 diabetes mellitus. Journal of Diabetes. 2013;5(3):261-7.  79. Yu DC, Chen WB, Jiang CP, Ding YT. Risk assessment in patients undergoing liver resection. Hepatobiliary and Pancreatic Diseases International. 2013;12(5):473-9.  80. Carter LG, D'Orazio JA, Pearson KJ. Resveratrol and cancer: Focus on in vivo evidence. Endocrine-Related Cancer. 2014;21(3):R209-R25.  81. Haugaard SB. Alteration in pancreatic islet function in human immunodeficiency virus. Endocrinology and Metabolism Clinics of North America. 2014;43(3):697-708.  82. Iepsen EW, Torekov SS, Holst JJ. Therapies for inter-relating diabetes and obesity - GLP-1 and obesity. Expert Opinion on Pharmacotherapy. 2014;15(17):2487-500.  83. Skolnik NS, Ryan DH. Pathophysiology, epidemiology, and assessment of obesity in adults. Journal of Family Practice. 2014;63(7):S3-S10.  84. Tutino GE, Tam WH, Yang X, Chan JCN, Lao TTH, Ma RCW. Diabetes and pregnancy: Perspectives from Asia. Diabetic Medicine. 2014;31(3):302-18.  85. Wu J, Liu S, Yu J, Zhou G, Rao D, Jay CM, et al. Vertically integrated translational studies of PDX1 as a therapeutic target for pancreatic cancer via a novel bifunctional RNAi platform. Cancer Gene Therapy. 2014;21(2):48-53.  86. Sunagawa Y, Katanasaka Y, Hasegawa K, Morimoto T. Clinical applications of curcumin. PharmaNutrition. 2015;3(4):131-5.  87. Vergès B, Cariou B. MTOR inhibitors and diabetes. Diabetes Research and Clinical Practice. 2015;110(2):101-8.  88. Best CJ, Thosani S, Ortiz M, Levesque C, Varghese SS, Lavis VR. Co-Managing Patients with Type 1 Diabetes and Cancer. Current Diabetes Reports. 2016;16(8).  89. Oetjen E. Regulation of Beta-Cell Function and Mass by the Dual Leucine Zipper Kinase. Archiv der Pharmazie. 2016;349(6):410-3.  90. Rizos CV, Kei A, Elisaf MS. The current role of thiazolidinediones in diabetes management. Archives of Toxicology. 2016;90(8):1861-81.  91. Vigneri R, Goldfine ID, Frittitta L. Insulin, insulin receptors, and cancer. Journal of Endocrinological Investigation. 2016;39(12):1365-76.  92. Wewer Albrechtsen NJ, Challis BG, Damjanov I, Holst JJ. Do glucagonomas always produce glucagon? Bosnian journal of basic medical sciences. 2016;16(1):1-7.  93. Barton JC, Acton RT. Diabetes in HFE Hemochromatosis. Journal of Diabetes Research. 2017;2017((Barton J.C., ironmd@isp.com; Acton R.T., rtakma@bellsouth.net) Southern Iron Disorders Center, Birmingham, AL, United States).  94. González-Domínguez R, Sayago A, Fernández-Recamales Á. Direct infusion mass spectrometry for metabolomic phenotyping of diseases. Bioanalysis. 2017;9(1):131-48.  95. Khoury T, Asombang AW, Berzin TM, Cohen J, Pleskow DK, Mizrahi M. The Clinical Implications of Fatty Pancreas: A Concise Review. Digestive Diseases and Sciences. 2017;62(10):2658-67.  96. Öberg K. Medical Therapy of Gastrointestinal Neuroendocrine Tumors. Visceral Medicine. 2017;33(5):352-6.  97. Andersen A, Lund A, Knop FK, Vilsbøll T. Glucagon-like peptide 1 in health and disease. Nature Reviews Endocrinology. 2018;14(7):390-403.  98. Beger HG, Mayer B. Early postoperative and late metabolic morbidity after pancreatic resections: An old and new challenge for surgeons – A review. American Journal of Surgery. 2018;216(1):131-4.  99. Dang YF, Jiang XN, Gong FL, Guo XL. New insights into molecular mechanisms of rosiglitazone in monotherapy or combination therapy against cancers. Chemico-Biological Interactions. 2018;296((Dang Y.-F.; Jiang X.-N.; Gong F.-L.; Guo X.-L., guoxl@sdu.edu.cn) Department of Pharmacology, Key Laboratory of Chemical Biology (Ministry of Education), Drug Screening Unit Platform, School of Pharmaceutical Sciences, Shandong University, Jinan, China):162-70.  100. Gallo M, Ruggeri RM, Muscogiuri G, Pizza G, Faggiano A, Colao A. Diabetes and pancreatic neuroendocrine tumours: Which interplays, if any? Cancer Treatment Reviews. 2018;67((Gallo M., mgallo4@cittadellasalute.to.it) Oncological Endocrinology Unit, Department of Medical Sciences, University of Turin, AOU Città della Salute e della Scienza di Torino, Turin, Italy):1-9.  101. Kakehi E, Kotani K, Nakamura T, Takeshima T, Kajii E. Non-diabetic glucose levels and cancer mortality: A literature review. Current Diabetes Reviews. 2018;14(5):434-45.  102. Khadka R, Tian W, Hao X, Koirala R. Risk factor, early diagnosis and overall survival on outcome of association between pancreatic cancer and diabetes mellitus: Changes and advances, a review. International Journal of Surgery. 2018;52((Khadka R., rkc_lucky@yahoo.com; Tian W., jonathan_weijun@yahoo.com; Hao X.) Department of General Surgery, Tianjin Medical University General Hospital, Tianjin, China):342-6.  103. Madhani K, Farrell JJ. Management of Autoimmune Pancreatitis. Gastrointestinal Endoscopy Clinics of North America. 2018;28(4):493-519.  104. Abudawood M. Diabetes and cancer: A comprehensive review. Journal of Research in Medical Sciences. 2019;24(1).  105. Böni-Schnetzler M, Meier DT. Islet inflammation in type 2 diabetes. Seminars in Immunopathology. 2019;41(4):501-13.  106. Chen B, Li J, Chi D, Sahnoune I, Calin S, Girnita L, et al. Non-coding RNAs in IGF-1R signaling regulation: The underlying pathophysiological link between diabetes and cancer. Cells. 2019;8(12).  107. Grieco GE, Brusco N, Licata G, Nigi L, Formichi C, Dotta F, et al. Targeting microRNAs as a therapeutic strategy to reduce oxidative stress in diabetes. International Journal of Molecular Sciences. 2019;20(24).  108. Maulucci G, Cohen O, Daniel B, Ferreri C, Sasson S. The Combination of Whole Cell Lipidomics Analysis and Single Cell Confocal Imaging of Fluidity and Micropolarity Provides Insight into Stress-Induced Lipid Turnover in Subcellular Organelles of Pancreatic Beta Cells. Molecules (Basel, Switzerland). 2019;24(20).  109. Vila G, Jørgensen JOL, Luger A, Stalla GK. Insulin resistance in patients with acromegaly. Frontiers in Endocrinology. 2019;10((Vila G.; Luger A.) Division of Endocrinology and Metabolism, Department of Internal Medicine III, Medical University of Vienna, Vienna, Austria).  110. Biondo LA, Teixeira AAS, Ferreira KCOS, Neto JCR. Pharmacological strategies for insulin sensitivity in obesity and cancer: Thiazolidinediones and metformin. Current Pharmaceutical Design. 2020;26(9):932-45.  111. Buha A, Dukić-Ćosić D, Ćurčić M, Bulat Z, Antonijević B, Moulis JM, et al. Emerging links between cadmium exposure and insulin resistance: Human, animal, and cell study data. Toxics. 2020;8(3).  112. El Hini SH, Ahmed ATZ, Hamed EMS, Mahmoud YZ, Eldin AMK, Abdelghany HM. Pivotal role of both tnf-α 238g/a and tcf7l2 c/t gene polymorphisms in type 2 diabetes. Open Access Macedonian Journal of Medical Sciences. 2020;8(F):283-6.  113. Kaleağasıoğlu F, Ali DM, Berger MR. Multiple Facets of Autophagy and the Emerging Role of Alkylphosphocholines as Autophagy Modulators. Frontiers in Pharmacology. 2020;11((Kaleağasıoğlu F.) Department of Pharmacology, Faculty of Medicine, Near East University, Mersin, Turkey).  114. Lawal Y, Bello F, Kaoje YS. Prediabetes deserves more attention: A review. Clinical Diabetes. 2020;38(4):328-38.  115. Vivot K, Pasquier A, Goginashvili A, Ricci R. Breaking Bad and Breaking Good: β-Cell Autophagy Pathways in Diabetes. Journal of Molecular Biology. 2020;432(5):1494-513.  116. Bessell E, Markovic TP, Fuller NR. How to provide a structured clinical assessment of a patient with overweight or obesity. Diabetes, Obesity and Metabolism. 2021;23(S1):36-49.  117. Calderón-Hernández MF, Revilla-Monsalve C, Altamirano-Bustamante MM, Altamirano-Bustamante NF, Belen Mosquera-Andrade M. What can we learn from β-cell failure biomarker application in diabetes in childhood? A systematic review. World Journal of Diabetes. 2021;12(8):1325-62.  118. Cheng HC, Chang TK, Su WC, Tsai HL, Wang JY. Narrative review of the influence of diabetes mellitus and hyperglycemia on colorectal cancer risk and oncological outcomes. Translational Oncology. 2021;14(7).  119. Di Giuseppe G, Ciccarelli G, Cefalo CM, Cinti F, Moffa S, Impronta F, et al. Prediabetes: how pathophysiology drives potential intervention on a subclinical disease with feared clinical consequences. Minerva Endocrinology. 2021;46(3):272-92.  120. Duggal N, Kapoor S. Role of gut microbiota in pathogenesis and treatment of type 2 diabetes. Eurasian Journal of Medicine and Oncology. 2021;5(2):103-10.  121. Elnashar M, Vaccarezza M, Al-Salami H. Cutting-edge biotechnological advancement in islet delivery using pancreatic and cellular approaches. Future Science OA. 2021;7(3).  122. Khan U, Chowdhury S, Billah MM, Islam KMD, Thorlacius H, Rahman M. Neutrophil extracellular traps in colorectal cancer progression and metastasis. International Journal of Molecular Sciences. 2021;22(14).  123. Li X, Celotto S, Pizzol D, Gasevic D, Ji MM, Barnini T, et al. Metformin and health outcomes: An umbrella review of systematic reviews with meta-analyses. European Journal of Clinical Investigation. 2021;51(7).  124. Nath P, Anand AC. Hepatogenous Diabetes: A Primer. Journal of Clinical and Experimental Hepatology. 2021;11(5):603-15.  125. Penaforte-Saboia JG, Couri CEB, Albuquerque NV, Silva VLL, Olegario NBDC, Fernandes VO, et al. Emerging roles of dipeptidyl peptidase-4 inhibitors in delaying the progression of type 1 diabetes mellitus. Diabetes, Metabolic Syndrome and Obesity. 2021;14((Penaforte-Saboia J.G.; Albuquerque N.V.; Olegario N.B.D.C.; Fernandes V.O.; Montenegro Junior R.M., renanmmjr@gmail.com) Clinical Research Unit, Walter Cantidio University Hospital, Federal University of Ceará, Fortaleza, Brazil):565-73.  126. Sarma S, Sockalingam S, Dash S. Obesity as a multisystem disease: Trends in obesity rates and obesity-related complications. Diabetes, Obesity and Metabolism. 2021;23(S1):3-16.  127. Sharp RC, Brown ME, Shapiro MR, Posgai AL, Brusko TM. The Immunoregulatory Role of the Signal Regulatory Protein Family and CD47 Signaling Pathway in Type 1 Diabetes. Frontiers in Immunology. 2021;12((Sharp R.C.; Brown M.E.; Shapiro M.R.; Posgai A.L.; Brusko T.M.) Department of Pathology, Immunology, and Laboratory Medicine, College of Medicine, University of Florida, Gainesville, FL, United States).  128. Toledo FGS, Chari S, Yadav D. Understanding the Contribution of Insulin Resistance to the Risk of Pancreatic Cancer. American Journal of Gastroenterology. 2021;116(4):669-70.  129. Villacreses MMC, Panjawatanan P, Chiu KC, Karnchanasorn R, Ou HY. Conundrum of vitamin D on glucose and fuel homeostasis. World Journal of Diabetes. 2021;12(9):1363-85.  130. Xia LZ, Bu XF, Jiang PC, Yu F, Zhang YJ, Meng NN. Agenesis of the dorsal pancreas with chronic suppurative pancreatitis: Case report and literature review. Medicine (United States). 2021;100(49).  131. Zhang X, Jia H, Li F, Fang C, Zhen J, He Q, et al. Ectopic insulinoma diagnosed by 68Ga-Exendin-4PET/CT: A case report and review of literature. Medicine (United States). 2021;100(13):E25076.  132. Bolanowski M, Kałużny M, Witek P, Jawiarczyk-Przybyłowska A. Pasireotide—a novel somatostatin receptor ligand after 20 years of use. Reviews in Endocrine and Metabolic Disorders. 2022;23(3):601-20.  133. Chen S, Gan D, Lin S, Zhong Y, Chen M, Zou X, et al. Metformin in aging and aging-related diseases: clinical applications and relevant mechanisms. Theranostics. 2022;12(6):2722-40.  134. Esmaeilzadeh A, Elahi R, Siahmansouri A, Maleki AJ, Moradi A. Endocrine and metabolic complications of COVID-19: lessons learned and future prospects. Journal of Molecular Endocrinology. 2022;69(3):R125-R50.  135. Kushchayeva Y, Kushchayev S, Jensen K, Brown RJ. Impaired Glucose Metabolism, Anti-Diabetes Medications, and Risk of Thyroid Cancer. Cancers. 2022;14(3).  136. Ni X, Zhang L, Feng X, Tang L. New Hypoglycemic Drugs: Combination Drugs and Targets Discovery. Frontiers in Pharmacology. 2022;13((Ni X.; Zhang L.; Feng X., junxiaofeng132@ustc.edu.cn; Tang L., tangliqin@ustc.edu.cn) Department of Pharmacy, The First Affiliated Hospital of University of USTC, Division of Life Sciences and Medicine, University of Science and Technology of China (USTC), Hefei, China).  137. Nikolaev G, Robeva R, Konakchieva R. Membrane melatonin receptors activated cell signaling in physiology and disease. International Journal of Molecular Sciences. 2022;23(1).  138. Pánico P, Velasco M, Salazar AM, Picones A, Ortiz-Huidobro RI, Guerrero-Palomo G, et al. Is Arsenic Exposure a Risk Factor for Metabolic Syndrome? A Review of the Potential Mechanisms. Frontiers in Endocrinology. 2022;13((Pánico P.; Velasco M.; Picones A.; Ortiz-Huidobro R.I.; Salgado-Bernabé M.E.; Hiriart M., mhiriart@ifc.unam.mx) Department of Cognitive Neurosciences, Instituto de Fisiología Celular, Universidad Nacional Autónoma de México, Mexico City, Mexico).  139. Romano A, Del Vescovo E, Rivetti S, Triarico S, Attinà G, Mastrangelo S, et al. Biomarkers Predictive of Metabolic Syndrome and Cardiovascular Disease in Childhood Cancer Survivors. Journal of Personalized Medicine. 2022;12(6).  140. Roshanravan N, Ghaffari S. The therapeutic potential of Crocus sativus Linn.: A comprehensive narrative review of clinical trials. Phytotherapy Research. 2022;36(1):98-111.  141. Tchéoubi SER, Akpovi CD, Coppée F, Declèves AE, Laurent S, Agbangla C, et al. Molecular and cellular biology of PCSK9: impact on glucose homeostasis. Journal of Drug Targeting. 2022;30(9):948-60.  142. Wagner R, Eckstein SS, Yamazaki H, Gerst F, Machann J, Jaghutriz BA, et al. Metabolic implications of pancreatic fat accumulation. Nature Reviews Endocrinology. 2022;18(1):43-54.  143. Zhang X, Dong Y, Liu D, Yang L, Xu J, Wang Q. Antigen-specific immunotherapies in type 1 diabetes. Journal of Trace Elements in Medicine and Biology. 2022;73((Zhang X.; Liu D.; Yang L.; Wang Q., wang_qing@jlu.edu.cn) Department of Endocrinology, China-Japan Union Hospital of Jilin University, Changchun, China).  144. Al-Rawi MBA, Khan AH, Iqbal MS, El Olemy AT. COVID-19 Versus Diabetes Mellitus: Whom Affect the Other? Asian Journal of Pharmaceutics. 2023;17(1):12-9.  145. Antar SA, Ashour NA, Sharaky M, Khattab M, Zaid RT, Roh EJ, et al. Diabetes mellitus: Classification, mediators, and complications; A gate to identify potential targets for the development of new effective treatments. Biomedicine and Pharmacotherapy. 2023;168((Antar S.A.) Center for Vascular and Heart Research, Fralin Biomedical Research Institute, Virginia Tech, Roanoke, VA, United States).  146. Das H, Ghosh S, Ganguly D, Malakar S, Biswas P, Ghosh D, et al. CURRENT SCENARIO OF DIABETIC MELLITUS WITH RECENT DRUG DEVELOPMENT APPROACHES: A COMPREHENSIVE REVIEW. NeuroQuantology. 2023;21(1):801-23.  147. Gajera D, Trivedi V, Thaker P, Rathod M, Dharamsi A. Detailed Review on Gestational Diabetes Mellitus with Emphasis on Pathophysiology, Epidemiology, Related Risk Factors, and its Subsequent Conversion to Type 2 Diabetes Mellitus. Hormone and Metabolic Research. 2023;55(5):295-303.  148. Gupta UC, Gupta SC, Gupta SS. An Evidence-Based Review of Diabetes Care: History, Types, Relationship to Cancer and Heart Disease, Co-Morbid Factors, and Preventive Measures. Current Nutrition and Food Science. 2023;19(4):399-408.  149. Hasan I, Rainsford KD, Ross JS. Salsalate: a pleotropic anti-inflammatory drug in the treatment of diabetes, obesity, and metabolic diseases. Inflammopharmacology. 2023;31(6):2781-97.  150. Hinnen D, Kruger D, Magwire M. Type 2 diabetes and cardiovascular disease: risk reduction and early intervention. Postgraduate Medicine. 2023;135(1):2-12.  151. Kabir MI, Kumar R, Bugata LSP, Raina K. Anti-cancer Efficacy of Metformin: Recent Updates on Breast and Other Cancers. Current Pharmacology Reports. 2023;9(5):284-328.  152. Marafie SK, Al-Mulla F. An Overview of the Role of Furin in Type 2 Diabetes. Cells. 2023;12(19).  153. Mareschal J, Hemmer A, Douissard J, Dupertuis YM, Collet TH, Koessler T, et al. Surgical Prehabilitation in Patients with Gastrointestinal Cancers: Impact of Unimodal and Multimodal Programs on Postoperative Outcomes and Prospects for New Therapeutic Strategies—A Systematic Review. Cancers. 2023;15(6).  154. Szabó A, Váncsa S, Hegyi P, Váradi A, Forintos A, Filipov T, et al. Lifestyle-, environmental-, and additional health factors associated with an increased sperm DNA fragmentation: a systematic review and meta-analysis. Reproductive Biology and Endocrinology. 2023;21(1).  155. Verma N, Despa F. The association between renal accumulation of pancreatic amyloid-forming amylin and renal hypoxia. Frontiers in Endocrinology. 2023;14((Verma N., nirmal.verma@uky.edu; Despa F.) Department of Pharmacology and Nutritional Sciences, University of Kentucky, Lexington, KY, United States).  156. Yazlcl D, Yaplcl Eser H, Klylcl S, Sancak S, Sezer H, Uygur M, et al. Clinical Impact of Glucagon-Like Peptide-1 Receptor Analogs on the Complications of Obesity. Obesity Facts. 2023;16(2):149-63.  157. Chan KE, Ong EYH, Chung CH, Ong CEY, Koh B, Tan DJH, et al. Longitudinal Outcomes Associated With Metabolic Dysfunction-Associated Steatotic Liver Disease: A Meta-analysis of 129 Studies. Clinical Gastroenterology and Hepatology. 2024;22(3):488-98.e14. |
| Editorial (n = 10) | 1. Gapstur SM, Gann P. Is pancreatic cancer a preventable disease? JAMA. 2001;286(8):967-8.  2. Yanagisawa RT, LeRoith D. Preface. Medical Clinics of North America. 2007;91(6):xiii-xv.  3. Gotthardt M. Beta cell imaging - why we need it and what has been achieved. Current Pharmaceutical Design. 2010;16(14):1545-6.  4. Bytautiene E. A molecular meeting. Science Translational Medicine. 2013;5(192).  5. Lee DH. Lipoproteins and ß-cell functions: From basic to clinical data. Diabetes and Metabolism Journal. 2014;38(4):274-7.  6. Roeyen G, De Block C. A plea for more practical and clinically applicable criteria defining type 3c diabetes. Pancreatology. 2017;17(6):875.  7. Risch HA. Diabetes and pancreatic cancer: Both cause and effect. Journal of the National Cancer Institute. 2019;111(1).  8. Chari ST, Andersen DK. Metabolic Surveillance for Those at High Risk for Developing Pancreatic Cancer. Gastroenterology. 2021;161(5):1379-80.  9. Al Madhoun A, Hamasaki H. Editorial: Rising stars: Clinical diabetes 2021. Frontiers in Endocrinology. 2022;13((Al Madhoun A., ashraf.madhoun@dasmaninstitute.org) Genetics and Bioinformatics, Dasman Diabetes Institute, Dasman, Kuwait).  10. Tarantino G. NAFLD or MAFLD: That is the conundrum. Hepatobiliary and Pancreatic Diseases International. 2022;21(2):103-5. |
| Meta-analysis (n = 9) | 1. Heckman-Stoddard BM, Crandall JP, Edelstein SL, Hamman RF, Prorok PC, Ryan A, et al. Cancer outcomes in the diabetes prevention program outcomes study. Cancer Prevention Research. 2015;8(10).  2. Maisonneuve P. Epidemiology and risk factors of pancreatic cancer. European Journal of Cancer. 2016;57((Maisonneuve P.) Division of Epidemiology and Biostatistics, European Institute of Oncology, Milan, Italy):S4.  3. Tsuei J, Kwan C, Kirkeby K. Development of Type 1 Diabetes Mellitus on Immune Checkpoint Inhibitor. Journal of the Endocrine Society. 2019;3((Tsuei J.; Kwan C.; Kirkeby K.)).  4. Lu C, Ke L, Li J, Zhao H, Lu T, Mentis AFA, et al. Saffron (Crocus sativus L.) and health outcomes: a meta-research review of meta-analyses and an evidence mapping study. Phytomedicine. 2021;91((Lu C., cuncunlu2017@163.com; Li J.; Yang K., kehuyangebm2006@126.com) Evidence-Based Medicine Center, School of Basic Medical Sciences, Lanzhou University, Lanzhou, Gansu, China).  5. Durrani J, Sood A, Sharma B, Khattar K, Buradkar A. A Rare Case of Pembrolizumab Induced Diabetic Ketoacidosis. Journal of the Endocrine Society. 2022;6((Durrani J.; Sood A.; Sharma B.; Khattar K.; Buradkar A.)):A297.  6. Keesari PR, Pulakurthi YS, Kumar V, Appala N, Sadum N, Rida T, et al. Long-Term Risk of Pancreatic Cancer in Patients With Prediabetes: A Systematic Review and Meta-Analysis of Prospective Studies. American Journal of Gastroenterology. 2022;117(10):S19.  7. Sood A, Sharma B, Khattar K. A RARE CASE OF PEMBROLIZUMAB INDUCED DIABETIC KETOACIDOSIS. Journal of General Internal Medicine. 2022;37((Sood A.; Sharma B.; Khattar K.) Internal Medicine, Wright Center for Graduate Medical Education, Scranton, PA, United States):S414-S5.  8. Ong CEY, Chan KE, Ong EYH, Chung CH, Koh B, Tan DJH, et al. LONGITUDINAL OUTCOMES ASSOCIATED WITH NON-ALCOHOLIC FATTY LIVER DISEASE. A META-ANALYSIS OF 129 STUDIES. Gut. 2023;72((Ong C.E.-Y.; Chan K.-E.; Ong E.Y.-H.; Chung C.H.; Koh B.; Tan D.J.-H.; Lim W.-H.; Yong J.-N.; Xiao J.; Syn N.; Ng C.-H.) Yong Loo Lin School of Medicine, National University of Singapore, Singapore):A23.  9. Zahariev OJ, Bunduc S, Kovács A, Demeter D, Havelda L, Veres DS, et al. Risk factors for diabetes mellitus after acute pancreatitis: a systematic review and meta-analysis. Clinical Nutrition ESPEN. 2023;58((Zahariev O.J.; Havelda L.; Erőss B.) Centre for Pancreatic Diseases):460-1. |
| Letters (n = 6) | 1. Wolf S, Obolonczyk L, Sworczak K, Czapiewski P, Sledzinski Z. Renal cell carcinoma metastases to the pancreas and the thyroid gland 19 years after the primary tumour. Przeglad Gastroenterologiczny. 2015;10(3):185-9.  2. Dirice E, Kahraman S, De Jesus DF, El Ouaamari A, Basile G, Baker RL, et al. Increased β-cell proliferation before immune cell invasion prevents progression of type 1 diabetes. Nature Metabolism. 2019;1(5):509-18.  3. Xie J, Dai L, Tang X. Study on the correlation between the changes of TNFR1, TNF-α, and adiponectin in patients with gestational diabetes mellitus and insulin resistance. European Journal of Inflammation. 2019;17((Xie J.; Dai L., moufangzhina2817@163.com) Department of Obstetrics and Gynecology, China Medicine Hospital in Linyi City, Linyi, China).  4. Wright BA, Cannon ME, Ramsey LJ. Reversing the irreversible: Another potential benefit of CFTR modulators. Pediatric Pulmonology. 2020;55(11):2844-5.  5. Ad’hiah AH, Al-Bayatee NT, Ahmed AA. Coronavirus disease 19 and risk of hyperglycemia among Iraqi patients. Egyptian Journal of Medical Human Genetics. 2021;22(1).  6. Chatterjee A, Chahal P. Letter to the Editor Regarding Prospective Assessment for Prediabetes and New-Onset Diabetes in High-Risk Individuals Undergoing Pancreatic Cancer Screening. Gastroenterology. 2022;162(6):1778-9. |
| Irrelevant studies (n = 484) | 1. Tobe T, Kouchi M, Tanimura H, Huang CH. Hyperglycemia after gastrectomy as a prediabetic state. Clinical study of 100 postgastrectomy patients. Archives of surgery (Chicago, Ill : 1960). 1967;94(6):836-40.  2. Adadevoh BK, Lukanmbi FA. Insulin levels in Nigerian adults, children and pregnant women. Hormone and metabolic research Hormon- und Stoffwechselforschung Hormones et métabolisme. 1972;4(3):136-9.  3. Metz SA, Halter JB, Robertson RP. Induction of defective insulin secretion and impaired glucose tolerance by clonidine. Selective stimulation of metabolic alpha-adrenergic pathways. Diabetes. 1978;27(5):554-62.  4. Cassar J, Ghatei MA, Sarson DL. Enteroglucagon and GIP after oral glucose in patients with prolactinoma and acromegaly. Clinical Endocrinology. 1983;18(1):95-102.  5. Levran D, Modan M, Menczer J, Dulitzy M. Increased rate of glucose intolerance in endometrial cancer - A community-based study. Gynecologic and Obstetric Investigation. 1984;18(4):190-3.  6. Srikanta s, Ganda OP, Jackson RA. Pre-Type 1 (insulin-dependent) diabetes: Common endocrinological course despite immunological and immunogenetic heterogeneity. Diabetologia. 1984;27(SUPPL.):146-8.  7. Di Carlo V, Chiesa R, Pontiroli AE, Carlucci M, Staudacher C, Zerbi A, et al. Pancreatoduodenectomy with occlusion of the residual stump by Neoprene® injection. World Journal of Surgery. 1989;13(1):105-11.  8. Wiggins J, Geddes DM. Respiratory aspects of Shwachman's syndrome in adults. European Respiratory Journal. 1989;2(3):285-8.  9. Konomi K, Chijiiwa K, Katsuta T, Yamaguchi K. Pancreatic somatostatinoma: A case report and review of the literature. Journal of Surgical Oncology. 1990;43(4):259-65.  10. Smedmyr B, Wibell L, Simonsson B, Oberg G. Impaired glucose tolerance after autologous bone marrow transplantation. Bone Marrow Transplantation. 1990;6(2):89-92.  11. Cersosimo E, Pisters PWT, Pesola G, McDermott K, Bajorunas D, Brennan MF. Insulin secretion and action in patients with pancreatic cancer. Cancer. 1991;67(2):486-93.  12. Permert J, Larsson J, Ihse I, Pour PM. Diagnosis of pancreatic cancer: Alteration of glucose metabolism. International Journal of Pancreatology. 1991;9((Permert J.; Larsson J.; Ihse I.; Pour P.M.) Department of Surgery, University of Linkoping, Linkoping, Sweden):113-7.  13. Sicolo N, Federspil G, De Palo CB, Vettor R, Martini C, Scandellari C. Endocrine pancreatic function in pheochromocytoma. Journal of Endocrinological Investigation. 1991;14(3):225-9.  14. Chari ST, Mohan V, Jayanthi V, Snehalatha C, Malathi S, Viswanathan M, et al. Comparative study of the clinical profiles of alcoholic chronic pancreatitis and tropical chronic pancreatitis in Tamil Nadu, South India. Pancreas. 1992;7(1):52-8.  15. Smith GD, Egger M, Shipley MJ, Marmot MG. Post-challenge glucose concentration, impaired glucose tolerance, diabetes, and cancer mortality in men. American Journal of Epidemiology. 1992;136(9):1110-4.  16. Gullo L, Ancona D, Pezzilli R, Casadei R, Campione O. Glucose tolerance and insulin secretion in pancreatic cancer. Italian Journal of Gastroenterology. 1993;25(9):487-9.  17. Permert J, Ihse I, Jorfeldt L, Von Schenck H, Arnqvis HJ, Larsson J. Pancreatic cancer is associated with impaired glucose metabolism. European Journal of Surgery, Acta Chirurgica. 1993;159(2):101-7.  18. Pour PM, Permert J, Mogaki M, Fujii H, Kazakoff K. Endocrine aspects of exocrine cancer of the pancreas: Their patterns and suggested biologic significance. American Journal of Clinical Pathology. 1993;100(3):223-30.  19. Böhmer KP, Kolb H, Kuglin B, Zielasek J, Hübinger A, Lampeter EF, et al. Linear loss of insulin secretory capacity during the last six months preceding IDDM: No effect of antiedematous therapy with ketotifen. Diabetes Care. 1994;17(2):138-41.  20. Fogar P, Pasquali C, Basso D, Sperti C, Panozzo MP, Tessari G, et al. Diabetes mellitus in pancreatic cancer follow-up. Anticancer Research. 1994;14(6 B):2827-30.  21. Iser G, Pfohl M, Dorr U, Weiss EM, Seif FJ. Ectopic ACTH secretion due to a bronchopulmonary carcinoid localized by somatostatin receptor scintigraphy. Clinical Investigator. 1994;72(11):887-91.  22. Lin F, Yao E, Xu S, Wei J, Zhao Z, Huang Z, et al. Functional status of pancreatic islet in acute leukemia. Chinese Medical Journal. 1994;107(11):827-31.  23. Permert J, Larsson J, Westermark GT, Herrington MK, Christmanson L, Pour PM, et al. Islet amyloid polypeptide in patients with pancreatic cancer and diabetes. New England Journal of Medicine. 1994;330(5):313-8.  24. Fischer G, Spengler U, Neubrand M, Sauerbruch T. Isolated tuberculosis of the pancreas masquerading as a pancreatic mass. American Journal of Gastroenterology. 1995;90(12):2227-30.  25. Durinovic-Bellò I, Hummel M, Ziegler AG. Cellular immune response to diverse islet cell antigens in IDDM. Diabetes. 1996;45(6):795-800.  26. El Nawawy A, Soliman AT, El Azzouni O, Abbassy AA, Massoud MN, Marzouk S, et al. Interleukin-1-beta, tumour necrosis factor-alpha, islet-cell antibody, and insulin secretion in children with thalassemia major on long-term blood transfusion. Journal of Tropical Pediatrics. 1996;42(6):362-4.  27. Bonadonna RC, Bonora E. Glucose and free fatty acid metabolism in human obesity. Relationships to insulin resistance. Diabetes Reviews. 1997;5(1):21-51.  28. Evans JD, Eggo MC, Donovan IA, Bramhall SR, Neoptolemos JP. Serum levels of insulin-like growth factors (IGF-I and IGF-II) and their binding protein (IGFBP-3) are not elevated in pancreatic cancer. International Journal of Pancreatology. 1997;22(2):95-100.  29. Permert J, Larsson J, Fruin AB, Tatemoto K, Herrington MK, Von Schenck H, et al. Islet hormone secretion in pancreatic cancer patients with diabetes. Pancreas. 1997;15(1):60-8.  30. Wang F, Larsson J, Abdiu A, Gasslander T, Westermark P, Adrian TE, et al. Dissociated secretion of islet amyloid polypeptide and insulin in serum- free culture media conditioned by human pancreatic adenocarcinoma cell lines. International Journal of Pancreatology. 1997;21(2):157-64.  31. Iwahashi H, Itoh N, Yamagata K, Imagawa A, Nakajima H, Tomita K, et al. Molecular mechanisms of pancreatic beta-cell destruction in autoimmune diabetes: Potential targets for preventive therapy. Cytokines, Cellular and Molecular Therapy. 1998;4(1):45-51.  32. Hostens K, Pavlovic D, Zambre Y, Ling Z, Van Schravendijk C, Eizirik DL, et al. Exposure of human islets to cytokines can result in disproportionately elevated proinsulin release. Journal of Clinical Investigation. 1999;104(1):67-72.  33. Panicot L, Mas E, Thivolet C, Lombardo D. Circulating antibodies against an exocrine pancreatic enzyme in type 1 diabetes. Diabetes. 1999;48(12):2316-23.  34. Furukawa H, Hiratsuka M, Ishikawa O, Ikeda M, Imamura H, Masutani S, et al. Total gastrectomy with dissection of lymph nodes along the splenic artery: A pancreas-preserving method. Annals of Surgical Oncology. 2000;7(9):669-73.  35. Kasayama S, Otsuki M, Takagi M, Saito H, Sumitani S, Kouhara H, et al. Impaired β-cell function in the presence of reduced insulin sensitivity determines glucose tolerance status in acromegalic patients. Clinical Endocrinology. 2000;52(5):549-55.  36. Kretowski A, Myśliwiec J, Szelachowska M, Kinalski M, Kinalska I. Nicotinamide inhibits enhanced in vitro production of interleukin-12 and tumour necrosis factor-α in peripheral whole blood of people at high risk of developing Type 1 diabetes and people with newly diagnosed Type 1 diabetes. Diabetes Research and Clinical Practice. 2000;47(2):81-6.  37. Appelros S, Lindgren S, Borgström A. Short and long term outcome of severe acute pancreatitis. European Journal of Surgery. 2001;167(4):281-6.  38. Permert J, Herrington M, Kazakoff K, Pour PM, Adrian TE. The patterns of extrainsular endocrine cells in pancreatic cancer. Teratogenesis Carcinogenesis and Mutagenesis. 2001;21(1):69-81.  39. Schlosser W, Siech M, Görich J, Beger HG. Common bile duct stenosis in complicated chronic pancreatitis. Scandinavian Journal of Gastroenterology. 2001;36(2):214-9.  40. Araújo LMB, Porto MV, Netto EM, Ursich MJ. Association of acanthosis nigricans with race and metabolic disturbances in obese women. Brazilian Journal of Medical and Biological Research. 2002;35(1):59-64.  41. Brand RE, Ding XZ, Young CM, Adrian TE. The specificity of amylin for the diagnosis of pancreatic adenocarcinoma. International Journal of Gastrointestinal Cancer. 2002;31(1-3):123-8.  42. Çetin M, Çolak R, Bayram F, Altnbas M, Ünal A, Keleştimur F. High prevalence of diabetes in patients with pancreatic cancer in central Anatolia, Turkey. Diabetes Research and Clinical Practice. 2002;58(2):97-100.  43. Chin D, Oberfield SE, Silfen ME, McMahon DJ, Manibo AM, Accili D, et al. Proinsulin in girls: Relationship to obesity, hyperinsulinemia, and puberty. Journal of Clinical Endocrinology and Metabolism. 2002;87(10):4673-7.  44. Parkinson C, Drake WM, Roberts ME, Meeran K, Besser GM, Trainer PJ. A comparison of the effects of pegvisomant and octreotide on glucose, insulin, gastrin, cholecystokinin, and pancreatic polypeptide responses to oral glucose and a standard mixed meal. Journal of Clinical Endocrinology and Metabolism. 2002;87(4):1797-804.  45. Baldelli R, Battista C, Leonetti F, Ghiggi MR, Ribaudo MC, Paoloni A, et al. Glucose homeostasis in acromegaly: Effects of long-acting somatostatin analogues treatment. Clinical Endocrinology. 2003;59(4):492-9.  46. Cardozo AK, Proost P, Gysemans C, Chen MC, Mathieu C, Eizirik DL. IL-1β and IFN-γ induce the expression of diverse chemokines and IL-15 in human and rat pancreatic islet cells, and in islets from pre-diabetic NOD mice. Diabetologia. 2003;46(2):255-66.  47. Lu W, Resnick HE, Jain AK, Adams-Campbell LL, Jablonski KA, Gottlieb AM, et al. Effects of isolated post-challenge hyperglycemia on mortality in American Indians: The strong heart study. Annals of Epidemiology. 2003;13(3):182-8.  48. Ronchi CL, Orsi E, Giavoli C, Cappiello V, Epaminonda P, Beck-Peccoz P, et al. Evaluation of insulin resistance in acromegalic patients before and after treatment with somatostatin analogues. Journal of Endocrinological Investigation. 2003;26(6):533-8.  49. Schwartz GG, Il'Yasova D, Ivanova A. Urinary cadmium, impaired fasting glucose, and diabetes in the NHANES III. Diabetes Care. 2003;26(2):468-70.  50. Sigfrid LA, Cunningham JM, Beeharry N, Lortz S, Tiedge M, Lenzen S, et al. Cytokines and nitric oxide inhibit the enzyme activity of catalase but not its protein or mRNA expression in insulin-producing cells. Journal of Molecular Endocrinology. 2003;31(3):509-18.  51. Ishikawa O, Ohigashi H, Eguchi H, Yokoyama S, Yamada T, Takachi K, et al. Long-term follow-up of glucose tolerance function after pancreaticoduodenectomy: Comparison between pancreaticogastrostomy and pancreaticojejunostomy. Surgery. 2004;136(3):617-23.  52. Mohn A, Di Marzio A, Capanna R, Fioritoni G, Chiarelli F. Persistence of impaired pancreatic β-cell function in children treated for acute lymphoblastic leukaemia. Lancet. 2004;363(9403):127-8.  53. Netea RT, Erceg A, Pieters G, Hermus AR. A patient with long-standing skin lesions. Netherlands Journal of Medicine. 2004;62(11):453-62.  54. Pfeffer F, Koczan D, Adam U, Benz S, Von Dobschuetz E, Prall F, et al. Expression of connexin26 in islets of langerhans is associated with impaired glucose tolerance in patients with pancreatic adenocarcinoma. Pancreas. 2004;29(4):284-90.  55. Schatz D, Cuthbertson D, Atkinson M, Salzer MC, Winter W, Muir A, et al. Preservation of C-peptide secretion in subjects at high risk of developing type 1 diabetes mellitus - A new surrogate measure of non-progression? Pediatric Diabetes. 2004;5(2):72-9.  56. Tanaka Y, Asakawa T, Asagiri K, Akiyoshi K, Hikida S, Mizote H. Nesidioblastosis treated successfully by 85% pancreatectomy. Kurume Medical Journal. 2004;51(1):99-103.  57. Wiesli P, Schäffler E, Seifert B, Schmid C, Donath MY. Islet secretory capacity determines glucose homoeostasis in the face of insulin resistance. Swiss Medical Weekly. 2004;134(37-38):559-63.  58. Biarnés J, Fernández-Real JM, Fernández-Castañer M, García MDM, Soler J, Ricart W. Differential regulation of insulin action and tumor necrosis factor α system activity by metformin. Metabolism: Clinical and Experimental. 2005;54(2):235-9.  59. Osei K, Gaillard T, Cook C, Kaplow J, Bullock M, Schuster D. Discrepancies in the regulation of plasma adiponectin and TNF-α levels and adipose tissue gene expression in obese African Americans with glucose intolerance: A pilot study using rosiglitazone. Ethnicity and Disease. 2005;15(4):641-8.  60. Quraishi I, Collins S, Pestaner JP, Harris T, Bagasra O. Role of zinc and zinc transporters in the molecular pathogenesis of diabetes mellitus. Medical Hypotheses. 2005;65(5):887-92.  61. Shalitin S, Abrahami M, Lilos P, Phillip M. Insulin resistance and impaired glucose tolerance in obese children and adolescents referred to a tertiary-care center in Israel. International Journal of Obesity. 2005;29(6):571-8.  62. Ehrmann-Jósko A, Siemińska J, Górnicka B, Ziarkiewicz-Wróblewska B, Ziółkowski B, Muszyński J. Impaired glucose metabolism in colorectal cancer. Scandinavian Journal of Gastroenterology. 2006;41(9):1079-86.  63. Ishida S, Funakoshi A, Miyasaka K, Shimokata H, Ando F, Takiguchi S. Association of SH-2 containing inositol 5′-phosphatase 2 gene polymorphisms and hyperglycemia. Pancreas. 2006;33(1):63-7.  64. Janson ET. Treatment of neuroendocrine tumors with somatostatin analogs. Pituitary. 2006;9(3):249-56.  65. Kwan EP, Xie L, Sheu L, Nolan CJ, Prentki M, Betz A, et al. Munc13-1 deficiency reduces insulin secretion and causes abnormal glucose tolerance. Diabetes. 2006;55(5):1421-9.  66. McCallum RW, Parameswaran V, Burgess JR. Multiple endocrine neoplasia type 1 (MEN 1) is associated with an increased prevalence of diabetes mellitus and impaired fasting glucose. Clinical Endocrinology. 2006;65(2):163-8.  67. Peppas NA, Kavimandan NJ. Nanoscale analysis of protein and peptide absorption: Insulin absorption using complexation and pH-sensitive hydrogels as delivery vehicles. European Journal of Pharmaceutical Sciences. 2006;29(3-4 SPEC. ISS.):183-97.  68. Yuen KCJ, Dunger DB. Impact of treatment with recombinant human GH and IGF-I on visceral adipose tissue and glucose homeostasis in adults. Growth Hormone and IGF Research. 2006;16(SUPPL.):55-61.  69. da Silva RCQ, Miranda WL, Chacra AR, Dib SA. Insulin resistance, β-cell function, and glucose tolerance in Brazilian adolescents with obesity or risk factors for type 2 diabetes mellitus. Journal of Diabetes and its Complications. 2007;21(2):84-92.  70. Dankner R, Chetrit A, Segal P. Glucose tolerance status and 20 year cancer incidence. Israel Medical Association Journal. 2007;9(8):592-6.  71. Franczak T, Burak W, Grzeszczak W, Spychałowicz W, Mykała-Cieśla J. The prevalence of carbohydrates metabolism impairment in patients with neoplastic diseases (based on own material). Diabetologia Doswiadczalna i Kliniczna. 2007;7(3):150-3.  72. Jung HS, Choi SH, Noh JH, Ohi SH, Ahn YR, Lee MK, et al. Healthy Twin Birth After Autologous Islet Transplantation in a Pancreatectomized Patient Due to a Benign Tumor. Transplantation Proceedings. 2007;39(5):1723-5.  73. Katsumichi I, Pour PM. Diabetes mellitus in pancreatic cancer: is it a causal relationship? American Journal of Surgery. 2007;194(4 SUPPL.):S71-S5.  74. Mai VQ, Jones RC, Dickert JM, Clyde PW, Shakir KM. Plasma glucagon levels suppressed by a glucose load in a man with incidental pancreatic glucagonoma. Endocrine practice : official journal of the American College of Endocrinology and the American Association of Clinical Endocrinologists. 2007;13(7):780-4.  75. Sakurai A, Katai M, Yamashita K, Mori JI, Fukushima Y, Hashizume K. Long-term follow-up of patients with multiple endocrine neoplasia type 1. Endocrine Journal. 2007;54(2):295-302.  76. Sinha SK, Bhangoo A, Anhalt H, Maclaren N, Marshall JD, Collin GB, et al. Effect of metformin and rosiglitazone in a prepubertal boy with Alström syndrome. Journal of Pediatric Endocrinology and Metabolism. 2007;20(9):1045-52.  77. Skyler JS. Prediction and prevention of type 1 diabetes: Progress, problems, and prospects. Clinical Pharmacology and Therapeutics. 2007;81(5):768-71.  78. Batty GD, Kivimaki M, Gray L, Smith GD, Marmot MG, Shipley MJ. Cigarette smoking and site-specific cancer mortality: Testing uncertain associations using extended follow-up of the original Whitehall study. Annals of Oncology. 2008;19(5):996-1002.  79. Glode A, Abdelghany S. A dipeptidyl peptidase-IV inhibitor for the treatment of type 2 diabetes. Formulary. 2008;43(9):317-25.  80. Han SL, Chen J, Zhou HZ, Lan SH, Zhang PC, Zhu GB. Indications and surgical treatment of chronic pancreatitis. Hepatobiliary and Pancreatic Diseases International. 2008;7(6):638-42.  81. Litwin J, Dobrowolski S, Orłowska-Kunikowska E, Śledziński Z. Changes in glucose metabolism after Kausch-Whipple pancreatectomy in pancreatic cancer and chronic pancreatitis patients. Pancreas. 2008;36(1):26-30.  82. Murakami Y, Uemura K, Hayashidani Y, Sudo T, Hashimoto Y, Ohge H, et al. Long-term pancreatic endocrine function following pancreatoduodenectomy with pancreaticogastrostomy. Journal of Surgical Oncology. 2008;97(6):519-22.  83. Soriguer F, Rojo-Martínez G, Almaraz MC, Esteva I, Ruiz De Adana MS, Morcillo S, et al. Incidence of type 2 diabetes in southern Spain (Pizarra Study). European Journal of Clinical Investigation. 2008;38(2):126-33.  84. Thompson AM, Church TS, Janssen I, Katzmarzyk PT, Earnest CP, Blair SN. Cardiorespiratory fitness as a predictor of cancer mortality among men with pre- diabetes and diabetes. Diabetes Care. 2008;31(4):764-9.  85. Xu X, Ling Q, He ZL, Gao F, Zheng SS. Post-transplant diabetes mellitus in liver transplantation: Hangzhou experience. Hepatobiliary and Pancreatic Diseases International. 2008;7(5):465-70.  86. Bo S, Gambino R, Ciccone G, Rosato R, Milanesio N, Villois P, et al. Effects of TCF7L2 polymorphisms on glucose values after a lifestyle intervention. American Journal of Clinical Nutrition. 2009;90(6):1502-8.  87. Kamisawa T, Shimosegawa T, Okazaki K, Nishino T, Watanabe H, Kanno A, et al. Standard steroid treatment for autoimmune pancreatitis. Gut. 2009;58(11):1504-7.  88. Krechler T, Jachymova M, Pavlikova M, Vecka M, Zeman M, Krska Z, et al. Polymorphism -23HPhI in the promoter of insulin gene and pancreatic cancer: A pilot study. Neoplasma. 2009;56(1):26-32.  89. Martínez Martín FJ. Manidipine in hypertensive patients with metabolic syndrome: The MARIMBA study. Expert Review of Cardiovascular Therapy. 2009;7(7):863-9.  90. Riboldi M, Sharp GC, Baroni G, Chen GTY. Four-dimensional targeting error analysis in image-guided radiotherapy. Physics in Medicine and Biology. 2009;54(19):5995-6008.  91. Saruç M, Karaarslan M, Rasa K, Saygili Ö, Ince Ü, Baysal Ç, et al. Pancreatic cancer and glucose metabolism. Turkish Journal of Gastroenterology. 2009;20(4):257-60.  92. Breuer TGK, Menge BA, Banasch M, Uhl W, Tannapfel A, Schmidt WE, et al. Proinsulin levels in patients with pancreatic diabetes are associated with functional changes in insulin secretion rather than pancreatic β-cell area. European Journal of Endocrinology. 2010;163(4):551-8.  93. Doi M, Sugiyama T, Izumiyama H, Yoshimoto T, Hirata Y. Clinical features and management of ectopic ACTH syndrome at a single institute in Japan. Endocrine Journal. 2010;57(12):1061-9.  94. Eren E, Özkan TB, Papatya Çakir ED, Saǧlam H, Tarim Ö. Acquired generalized lipodystrophy associated with autoimmune hepatitis and low serum C4 level. JCRPE Journal of Clinical Research in Pediatric Endocrinology. 2010;2(1):39-42.  95. Gur C, Porgador A, Elboim M, Gazit R, Mizrahi S, Stern-Ginossar N, et al. The activating receptor NKp46 is essential for the development of type 1 diabetes. Nature Immunology. 2010;11(2):121-8.  96. Heni M, Machann J, Staiger H, Schwenzer NF, Peter A, Schick F, et al. Pancreatic fat is negatively associated with insulin secretion in individuals with impaired fasting glucose and/or impaired glucose tolerance: A nuclear magnetic resonance study. Diabetes/Metabolism Research and Reviews. 2010;26(3):200-5.  97. Hwang ST, Cho YK, Yun JW, Park JH, Kim HJ, Park DI, et al. Impact of non-alcoholic fatty liver disease on microalbuminuria in patients with prediabetes and diabetes. Internal Medicine Journal. 2010;40(6):437-42.  98. Iyer A, Brown L. Lipid mediators and inflammation in glucose intolerance and insulin resistance. Drug Discovery Today: Disease Mechanisms. 2010;7(3-4):e191-e7.  99. Krechler T, Jáchymová M, Mestek O, Žák A, Zima T, Kalousová M. Soluble receptor for advanced glycation end-products (sRAGE) and polymorphisms of RAGE and glyoxalase I genes in patients with pancreas cancer. Clinical Biochemistry. 2010;43(10-11):882-6.  100. Li SW, Koya V, Li Y, Donelan W, Lin P, Reeves WH, et al. Pancreatic duodenal homeobox 1 protein is a novel Β-cell-specific autoantigen for type i diabetes. Laboratory Investigation. 2010;90(1):31-9.  101. Matsukuma S, Sato K. Pancreatic neuroma-like lesions after upper abdominal surgery: A clinicopathological postmortem study. Virchows Archiv. 2010;457(6):651-7.  102. Nolan CJ. Failure of islet β-cell compensation for insulin resistance causes type 2 diabetes: What causes non-alcoholic fatty liver disease and non-alcoholic steatohepatitis? Journal of Gastroenterology and Hepatology (Australia). 2010;25(10):1594-7.  103. Pallayova M, Steele KE, Magnuson TH, Schweitzer MA, Hill NR, Bevans-Fonti S, et al. Sleep apnea predicts distinct alterations in glucose homeostasis and biomarkers in obese adults with normal and impaired glucose metabolism. Cardiovascular Diabetology. 2010;9((Pallayova M., maria.pallayova@upjs.sk; Bevans-Fonti S., sbevans1@jhmi.edu; Schwartz A.R., aschwar2@jhmi.edu) Johns Hopkins Sleep Disorders Center, Division of Pulmonary and Critical Care Medicine, Johns Hopkins University, Baltimore, MD, United States).  104. Pober BR, Wang E, Caprio S, Petersen KF, Brandt C, Stanley T, et al. High prevalence of diabetes and pre-diabetes in adults with Williams syndrome. American Journal of Medical Genetics, Part C: Seminars in Medical Genetics. 2010;154(2):291-8.  105. Reynier F, Pachot A, Paye M, Xu Q, Turrel-Davin F, Petit F, et al. Specific gene expression signature associated with development of autoimmune type-I diabetes using whole-blood microarray analysis. Genes and Immunity. 2010;11(3):269-78.  106. Roggli E, Britan A, Gattesco S, Lin-Marq N, Abderrahmani A, Meda P, et al. Involvement of microRNAs in the cytotoxic effects exerted by proinflammatory cytokines on pancreatic β-cells. Diabetes. 2010;59(4):978-86.  107. T Hart LM, Simonis-Bik AM, Nijpels G, Van Haeften TW, Schäfer SA, Houwing-Duistermaat JJ, et al. Combined risk allele score of eight type 2 diabetes genes is associated with reduced first-phase glucose-stimulated insulin secretion during hyperglycemic clamps. Diabetes. 2010;59(1):287-92.  108. Tabata R, Tabata C, Okamoto T, Omori K, Terada M, Nagai T. Autoimmune pancreatitis associated with myelodysplastic syndrome. International Archives of Allergy and Immunology. 2010;151(2):168-72.  109. Tsay J, Pomeranz C, Hassoun A, Zandieh SO, Rutledge J, Vogiatzi MG, et al. Screening markers of impaired glucose tolerance in the obese pediatric population. Hormone Research in Paediatrics. 2010;73(2):102-7.  110. Wang F, Larsson J, Herrington MK, Permert J. PP56 improves energy homeostasis in a mouse model of pancreatic cancer. Tumor Biology. 2010((Wang F., feng.wang@ki.se; Larsson J.; Herrington M.K.; Permert J.) Department of Clinical Science, Intervention and Technology, Division of Surgery, Karolinska University Hospital, Huddinge, Sweden):1-5.  111. Akaza I, Yoshimoto T, Iwashima F, Nakayama C, Doi M, Izumiyama H, et al. Clinical outcome of subclinical Cushing's syndrome after surgical and conservative treatment. Hypertension Research. 2011;34(10):1111-5.  112. Dagogo-Jack S, Edeoga C, Nyenwe E, Chapp-Jumbo E, Wan J. Pathobiology of prediabetes in a biracial cohort (POP-ABC): Design and methods. Ethnicity and Disease. 2011;21(1):33-9.  113. Kowall B, Rathmann W, Heier M, Giani G, Peters A, Thorand B, et al. Categories of glucose tolerance and continuous glycemic measures and mortality. European Journal of Epidemiology. 2011;26(8):637-45.  114. Arima H, Hiroi-Mizutani M, Okada Y, Oiso Y. Glucose tolerance was improved in an acromegalic patient treated with somatostatin analogue while insulin release was markedly suppressed. Diabetology International. 2012;3(2):99-102.  115. Bahíllo-Curieses MP, Hermoso-López F, Martínez-Sopena MJ, Cobreros-García P, García-Saseta P, Tríguez-García M, et al. Prevalence of insulin resistance and impaired glucose tolerance in a sample of obese Spanish children and adolescents. Endocrine. 2012;41(2):289-95.  116. Chen Y, Blaser MJ. Association between gastric helicobacter pylori colonization and glycated hemoglobin levels. Journal of Infectious Diseases. 2012;205(8):1195-202.  117. Chuengsamarn S, Rattanamongkolgul S, Luechapudiporn R, Phisalaphong C, Jirawatnotai S. Curcumin extract for prevention of type 2 diabetes. Diabetes Care. 2012;35(11):2121-7.  118. Dumbrava L, Popa A, Brink S. Risk factors for prediabetes in overweight and obese pre-teens and adolescents. Romanian Journal of Diabetes, Nutrition and Metabolic Diseases. 2012;19(3):255-63.  119. Fujihara S, Mori H, Kobara H, Nishiyama N, Kobayashi M, Oryu M, et al. Metabolic syndrome, obesity, and gastrointestinal cancer. Gastroenterology Research and Practice. 2012((Fujihara S., joshin@med.kagawa-u.ac.jp; Mori H., hiro4884@med.kagawa-u.ac.jp; Kobara H., kobara@med.kagawa-u.ac.jp; Nishiyama N., n-nori@med.kagawa-u.ac.jp; Kobayashi M., koba@med.kagawa-u.ac.jp; Oryu M., oryu@kkr-ta-hp.gr.jp; Masaki T., tmasaki@med.kagawa-u.ac.jp) Department of Gastroenterology and Neurology, Faculty of Medicine, Kagawa University, 1750-1 Ikenobe, Miki-cho, Kita-gun, Takamatsu, Kagawa 761-0793, Japan).  120. Gómez-Díaz RA, Talavera JO, Pool EC, Ortiz-Navarrete FV, Solórzano-Santos F, Mondragón-González R, et al. Metformin decreases plasma resistin concentrations in pediatric patients with impaired glucose tolerance: A placebo-controlled randomized clinical trial. Metabolism: Clinical and Experimental. 2012;61(9):1247-55.  121. Hirakawa Y, Ninomiya T, Mukai N, Doi Y, Hata J, Fukuhara M, et al. Association between Glucose Tolerance Level and Cancer Death in a General Japanese population. American Journal of Epidemiology. 2012;176(10):856-64.  122. Kim SK, Suh S, Lee JI, Hur KY, Chung JH, Lee MK, et al. The Ability of β-cells to compensate for insulin resistance is restored with a reduction in excess growth hormone in Korean acromegalic patients. Journal of Korean Medical Science. 2012;27(2):177-83.  123. Kuller LH. Metformin use among individuals at risk for type 2 diabetes. Current Diabetes Reports. 2012;12(3):265-73.  124. Meier JJ, Breuer TGK, Bonadonna RC, Tannapfel A, Uhl W, Schmidt WE, et al. Pancreatic diabetes manifests when beta cell area declines by approximately 65% in humans. Diabetologia. 2012;55(5):1346-54.  125. Mori Y, Ohtsuka T, Tsutsumi K, Yasui T, Ueda J, Takahata S, et al. Different incretin responses after pancreatoduodenectomy and distal pancreatectomy. Pancreas. 2012;41(3):455-60.  126. Muscaritoli M, Costelli P, Molfino A, Penna F, Baccino FM, Gioia G, et al. New strategies for metabolic support in cancer. Current Nutrition and Food Science. 2012;8(2):139-48.  127. Onur MR, Yalniz M, Poyraz AK, Özercan IH, Ozkan Y. Pancreatic islet cell amyloidosis manifesting as a large pancreas. Korean Journal of Radiology. 2012;13(1):94-7.  128. Roberts RE, Zhao M, Whitelaw BC, Ramage J, Diaz-Cano S, Le Roux CW, et al. GLP-1 and glucagon secretion from a pancreatic neuroendocrine tumor causing diabetes and hyperinsulinemic hypoglycemia. Journal of Clinical Endocrinology and Metabolism. 2012;97(9):3039-45.  129. Thondam SK, Shrotri M, Oyegade A, Daousi C, Cuthbertson DJ. Transient insulin-treated diabetes mellitus in a patient with pancreatic tuberculosis. Practical Diabetes. 2012;29(7):292-4.  130. Van Wijk JPH, Dreijerink KMA, Pieterman CRC, Lips CJM, Zelissen PMJ, Valk GD. Increased prevalence of impaired fasting glucose in MEN1 gene mutation carriers. Clinical Endocrinology. 2012;76(1):67-71.  131. Yeung WCG, Al-Shabeeb A, Pang CNI, Wilkins MR, Catteau J, Howard NJ, et al. Children with islet autoimmunity and enterovirus infection demonstrate a distinct cytokine profile. Diabetes. 2012;61(6):1500-8.  132. Åkerman L, Ludvigsson J, Casas R. Low C-peptide levels and decreased expression of TNF and CD45 in children with high risk of type 1 diabetes. Clinical Immunology. 2013;148(1):4-15.  133. Bottiglieri S, Tierson N, Patel R, Mo JH, Mehdi S. Gemcitabine-induced gouty arthritis attacks. Journal of Oncology Pharmacy Practice. 2013;19(3):284-8.  134. Goldstein MR, Mascitelli L. Do statins cause diabetes? Current Diabetes Reports. 2013;13(3):381-90.  135. Hata T, Sakata N, Aoki T, Yoshida H, Kanno A, Fujishima F, et al. Repeated pancreatectomy for metachronous duodenal and pancreatic metastases of renal cell carcinoma. Case Reports in Gastroenterology. 2013;7(3):442-8.  136. Hayashi SY, Faintuch J, Yagi OK, Yamaguchi CM, Faintuch JJ, Cecconello I. Does Roux-en-Y gastrectomy for gastric cancer influence glucose homeostasis in lean patients? Surgical Endoscopy. 2013;27(8):2829-35.  137. Home P. Insulin therapy and cancer. Diabetes Care. 2013;36(SUPPL.2):S240-S4.  138. Hsu CN, Chang CH, Lin YS, Lin JW, Caffrey JL. Association of Serum C-Peptide Concentrations with Cancer Mortality Risk in Pre-Diabetes or Undiagnosed Diabetes. PLoS ONE. 2013;8(2).  139. Huang H, Guo Q, Qiu C, Huang B, Fu X, Yao J, et al. Associations of green tea and rock tea consumption with risk of impaired fasting glucose and impaired glucose tolerance in Chinese men and women. PLoS ONE. 2013;8(11).  140. Janssen JAMJL, Lamberts SW. Diabetes associated with glucocorticoid excess. 2013. p. 22-33.  141. Jin SM, Oh SH, Kim SK, Jung HS, Choi SH, Jang KT, et al. Diabetes-free survival in patients who underwent islet Autotransplantation after 50% to 60% distal partial Pancreatectomy for benign pancreatic tumors. Transplantation. 2013;95(11):1396-403.  142. Karnevi E, Said K, Andersson R, Rosendahl AH. Metformin-mediated growth inhibition involves suppression of the IGF-I receptor signalling pathway in human pancreatic cancer cells. BMC Cancer. 2013;13((Karnevi E., emelie.karnevi@med.lu.se; Said K., katarzyna.said@med.lu.se; Andersson R., roland.andersson@med.lu.se; Rosendahl A.H., ann.rosendahl@med.lu.se) Department of Surgery, Clinical Sciences Lund, Skåne University Hospital and Lund University, Lund SE-221 84, Sweden).  143. Kasuga M, Ueki K, Tajima N, Noda M, Ohashi K, Noto H, et al. Report of the Japan diabetes society/Japanese cancer association joint committee on diabetes and cancer. Cancer Science. 2013;104(7):965-76.  144. Kibirige D, Ssekitoleko R. Endocrine and metabolic abnormalities among HIV-infected patients: A current review. International Journal of STD and AIDS. 2013;24(8):603-11.  145. Kos-Kudła B, Blicharz-Dorniak J, Handkiewicz-Junak D, Jarza̧b B, Jarza̧b M, Kunikowska J, et al. Diagnostic and therapeutic guidelines for gastro-entero-pancreatic neuroendocrine neoplasms (recommended by the Polish Network of Neuroendocrine Tumours). Endokrynologia Polska. 2013;64(6):418-43.  146. Li XN, Herrington J, Petrov A, Ge L, Eiermann G, Xiong Y, et al. The role of voltage-gated potassium channels Kv2.1 and Kv2.2 in the regulation of insulin and somatostatin release from pancreatic isletss. Journal of Pharmacology and Experimental Therapeutics. 2013;344(2):407-16.  147. Ljubicic S, Bezzi P, Brajkovic S, Nesca V, Guay C, Ohbayashi N, et al. The GTPase Rab37 Participates in the Control of Insulin Exocytosis. PLoS ONE. 2013;8(6).  148. Lucas R, Parikh SJ, Sridhar S, Guo DH, Bhagatwala J, Dong Y, et al. Cytokine profiling of young overweight and obese female African American adults with prediabetes. Cytokine. 2013;64(1):310-5.  149. Majumder S, Berzin TM, Mahadevan A, Pawa R, Ellsmere J, Sepe PS, et al. Endoscopic ultrasound-guided pancreatic fiducial placement: How important is ideal fiducial geometry? Pancreas. 2013;42(4):692-5.  150. Marchetti P, Bugliani M, Boggi U, Masini M, Marselli L. The pancreatic β cells in human type 2 diabetes. 2013. p. 288-309.  151. Minuto F, Ferone D, Boschetti M, Albertelli M, Gatto F. Diabetes secondary to neuroendocrine gastroenteropancreatic tumors. 2013. p. 64-76.  152. Neggers SJ, Van Der Lely AJ. Diabetes from growth hormone excess. 2013. p. 1-9.  153. Rose AJ, Herzig S. Metabolic control through glucocorticoid hormones: An update. Molecular and Cellular Endocrinology. 2013;380(1-2):65-78.  154. Soumaya K. Molecular mechanisms of insulin resistance in diabetes. 2013. p. 240-51.  155. Thakkar U, Vanikar A, Trivedi H. Co-infusion of autologous adipose tissue derived insulin-secreting mesenchymal stem cells and bone marrow derived hematopoietic stem cells: Viable therapy for type III C. a diabetes mellitus. Biomedical Journal. 2013;36(6):304-7.  156. Thomas A, Kautzky-Willer A. Diabetes in hyperparathyroidism. 2013. p. 92-100.  157. Tiengo A, De Kreutzenberg SV, Del Prato S. Diabetes in pancreatitis, pancreatectomy and other pancreatic diseases. 2013. p. 119-43.  158. Umeno A, Shichiri M, Ishida N, Hashimoto Y, Abe K, Kataoka M, et al. Singlet Oxygen Induced Products of Linoleates, 10- and 12-(Z,E)-Hydroxyoctadecadienoic Acids (HODE), Can Be Potential Biomarkers for Early Detection of Type 2 Diabetes. PLoS ONE. 2013;8(5).  159. Xiang YF, Zhao YJ, Zhou ZG. Latent autoimmune diabetes in adults: Evidences for diabetes spectrum? Chinese Medical Journal. 2013;126(4):783-8.  160. Agrawal NK, Kant S. Targeting inflammation in diabetes: Newer therapeutic options. World Journal of Diabetes. 2014;5(5):697-710.  161. Alexopoulou O, Bex M, Kamenicky P, Mvoula AB, Chanson P, Maiter D. Prevalence and risk factors of impaired glucose tolerance and diabetes mellitus at diagnosis of acromegaly: A study in 148 patients. Pituitary. 2014;17(1):81-9.  162. Almoosawi S, Cole D, Nicholson S, Bayes I, Teucher B, Bates B, et al. Biomarkers of diabetes risk in the National Diet and Nutrition Survey rolling programme (2008-2011). Journal of Epidemiology and Community Health. 2014;68(1):51-6.  163. Andersen GS, Thybo T, Cederberg H, Orešič M, Esteller M, Zorzano A, et al. The DEXLIFE study methods: Identifying novel candidate biomarkers that predict progression to type 2 diabetes in high risk individuals. Diabetes Research and Clinical Practice. 2014;106(2):383-9.  164. Borné Y, Fagerberg B, Persson M, Sallsten G, Forsgard N, Hedblad B, et al. Cadmium exposure and incidence of diabetes mellitus - Results from the Malmö Diet and Cancer study. PLoS ONE. 2014;9(11).  165. Ceriello A, Barakat M, Bahendeka S, Colagiuri S, Gerich J, Hanefeld M, et al. Guideline for management of postmeal glucose in diabetes. Diabetes Research and Clinical Practice. 2014;103(2):256-68.  166. Chatterjee S, Roy N, Saha A, Roy S, Chatterjee A, Hazra N, et al. Black tea consumption enhance antioxidant status, reduce inflammatory stress vis-a-vis insulin resistance: Hint from a small clinical cohort study on pre-diabetic subjects. International Journal of Pharmaceutical Sciences Review and Research. 2014;28(2):278-83.  167. Cordero-Franco HF, Salinas-Martínez AM, Abundis A, Espinosa-Flores EM, Vázquez-Lara J, Guerrero-Romero F. The effect of insulin resistance on breast cancer risk in Latinas of Mexican origin. Metabolic Syndrome and Related Disorders. 2014;12(9):477-83.  168. De Beer JC, Liebenberg L. Does cancer risk increase with HbA 1c, independent of diabetes? British Journal of Cancer. 2014;110(9):2361-8.  169. Engström G, Smith JG, Persson M, Nilsson PM, Melander O, Hedblad B. Red cell distribution width, haemoglobin A1c and incidence of diabetes mellitus. Journal of Internal Medicine. 2014;276(2):174-83.  170. Gagnon C, Daly RM, Carpentier A, Lu ZX, Shore-Lorenti C, Sikaris K, et al. Effects of combined calcium and vitamin D supplementation on insulin secretion, insulin sensitivity and β-cell function in multi-ethnic vitamin D-deficient adults at risk for type 2 diabetes: A pilot randomized, placebo-controlled trial. PLoS ONE. 2014;9(10).  171. Homsak E. Diabetes as autoimmune disease - Diabetes type I. Biochemia Medica. 2014;24(3):S35-S8.  172. Huang Y, Cai X, Qiu M, Chen P, Tang H, Hu Y. Prediabetes and the risk of cancer: a meta-analysis. Diabetologia. 2014;57(11):2261-9.  173. Janssen JAMJL, Lamberts SW. Diabetes associated with glucocorticoid excess. 2014. p. 22-33.  174. Karnevi E, Andersson R, Rosendahl AH. Tumour-educated macrophages display a mixed polarisation and enhance pancreatic cancer cell invasion. Immunology and Cell Biology. 2014;92(6):543-52.  175. Kim HJ, Ahn HY, Kwak JH, Shin DY, Kwon YI, Oh CG, et al. The effects of chitosan oligosaccharide (GO2KA1) supplementation on glucose control in subjects with prediabetes. Food & function. 2014;5(10):2662-9.  176. Kinoshita H, Miyagatani Y, Murao M, Kamimura Y. Regular follow-up of olanzapine blood levels and impaired glucose tolerance in olanzapine-induced diabetic ketoacidosis: A case report. Clinical Neuropsychopharmacology and Therapeutics. 2014;5((Kinoshita H., haru.kinoshita@gmail.com) Department of Cardiology, National Hospital Organization Kure Medical Center and Chugoku Cancer Center, Kure, Hiroshima, Japan).  177. Kumar NP, Banurekha VV, Nair D, Sridhar R, Kornfeld H, Nutman TB, et al. Coincident pre-diabetes is associated with dysregulated cytokine responses in pulmonary tuberculosis. PLoS ONE. 2014;9(11).  178. Li MJ, Peng SSF, Lu MY, Chang HH, Yang YL, Jou ST, et al. Diabetes mellitus in patients with thalassemia major. Pediatric Blood and Cancer. 2014;61(1):20-4.  179. Li W, Zhang S, Liu H, Wang L, Zhang C, Leng J, et al. Different associations of diabetes withβ-celldysfunction and insulin resistance among obese and nonobese chinesewomen with prior gestational diabetes mellitus. Diabetes Care. 2014;37(9):2533-9.  180. Lu LJ, Gan L, Hu JB, Ran L, Cheng QF, Wang RJ, et al. On the status of β-cell dysfunction and insulin resistance of breast cancer patient without history of diabetes after systemic treatment. Medical Oncology. 2014;31(5).  181. Matejak-Górska MA, Durlik M, Kaułza B, Milczarczyk A, Franek E. Preoperative glucose abnormalities in patients with pancreatic tumours. Przeglad Gastroenterologiczny. 2014;9(2):105-8.  182. Parker AR, Byham-Gray L, Denmark R, Winkle PJ. The effect of medical nutrition therapy by a registered dietitian nutritionist in patients with prediabetes participating in a randomized controlled clinical research trial. Journal of the Academy of Nutrition and Dietetics. 2014;114(11):1739-48.  183. Patel R, Ede J, Collins J, Willens D. Pancreatic cancer presenting as new-onset diabetes. Case Reports in Oncology. 2014;7(1):171-4.  184. Sakamaki JI, Fu A, Reeks C, Baird S, Depatie C, Al Azzabi M, et al. Role of the SIK2-p35-PJA2 complex in pancreatic β-cell functional compensation. Nature Cell Biology. 2014;16(3):234-44.  185. Sannappa RM, Buragohain J, Sarma D, Saikia UK, Choudhury BK. Agenesis of dorsal pancreas associated with periampullary pancreaticobiliary type adenocarcinoma. Journal of the Pancreas. 2014;15(5):489-92.  186. Stagi S, Lapi E, Cecchi C, Chiarelli F, D'Avanzo MG, Seminara S, et al. Williams-beuren syndrome is a genetic disorder associated with impaired glucose tolerance and diabetes in childhood and adolescence: New insights from a longitudinal study. Hormone Research in Paediatrics. 2014;82(1):38-43.  187. Wei C, Thyagiarajan M, Hunt L, Cox R, Bradley K, Elson R, et al. Reduced beta-cell reserve and pancreatic volume in survivors of childhood acute lymphoblastic leukaemia treated with bone marrow transplantation and total body irradiation. Clinical Endocrinology. 2014((Wei C.; Thyagiarajan M.; Hunt L.; Cox R.; Bradley K.; Elson R.; Hamilton-Shield J.; Stevens M.; Crowne E., Liz.Crowne@UHBristol.nhs.uk) Departments of Paediatric Endocrinology and Diabetes, Paediatric Oncology and Paediatric Radiology Bristol Royal Hospital for Children Bristol UK).  188. Westwell-Roper CY, Ehses JA, Verchere CB. Resident macrophages mediate islet amyloid polypeptide-induced islet IL-1β production and β-cell dysfunction. Diabetes. 2014;63(5):1698-711.  189. Yorifuji T, Hashimoto Y, Kawakita R, Hosokawa Y, Fujimaru R, Hatake K, et al. Relapsing 6q24-related transient neonatal diabetes mellitus successfully treated with a dipeptidyl peptidase-4 inhibitor: A case report. Pediatric Diabetes. 2014;15(8):606-10.  190. Zhang D, Zhang L, Zheng Y, Yue F, Russell RD, Zeng Y. Circulating zonulin levels in newly diagnosed Chinese type 2 diabetes patients. Diabetes Research and Clinical Practice. 2014;106(2):312-8.  191. Zhang P, Zou M, Wen X, Gu F, Li J, Liu G, et al. Development of serum parameters panels for the early detection of pancreatic cancer. International Journal of Cancer. 2014;134(11):2646-55.  192. Burch TC, Morris MA, Campbell-Thompson M, Pugliese A, Nadler JL, Nyalwidhe JO. Proteomic analysis of disease stratified human pancreas tissue indicates unique signature of type 1 diabetes. PLoS ONE. 2015;10(8).  193. Ehehalt F, Sturm D, Rösler M, Distler M, Weitz J, Kersting S, et al. Blood glucose homeostasis in the course of partial pancreatectomy - Evidence for surgically reversible diabetes induced by cholestasis. PLoS ONE. 2015;10(8).  194. Ferdaoussi M, Dai X, Jensen MV, Wang R, Peterson BS, Huang C, et al. Isocitrate-to-SENP1 signaling amplifies insulin secretion and rescues dysfunctional β cells. Journal of Clinical Investigation. 2015;125(10):3847-60.  195. Gupta SP, Mittal A, Jha DK, Pandeya DR. Association of fatty pancreas and diabetes. A case control study from Kathmandu Valley. Biomedical Research (India). 2015;26(2):389-91.  196. Hammer MJ, Aouizerat BE, Schmidt BL, Cartwright F, Wright F, Miaskowski C. Glycosylated Hemoglobin A1c and Lack of Association With Symptom Severity in Patients Undergoing Chemotherapy for Solid Tumors. Oncology nursing forum. 2015;42(6):581-90.  197. Jung YS, Shin MH, Kweon SS, Lee YH, Kim OJ, Kim YJ, et al. Periodontal disease associated with blood glucose levels in urban Koreans aged 50 years and older: the Dong-gu study. Gerodontology. 2015;32(4):267-73.  198. Ko KP, Ma SH, Yang JJ, Hwang Y, Ahn C, Cho YM, et al. Metformin intervention in obese non-diabetic patients with breast cancer: phase II randomized, double-blind, placebo-controlled trial. Breast Cancer Research and Treatment. 2015;153(2):361-70.  199. Kühn JP, Berthold F, Mayerle J, Völzke H, Reeder SB, Rathmann W, et al. Pancreatic steatosis demonstrated at MR imaging in the general population: Clinical relevance. Radiology. 2015;276(1):129-36.  200. Kwon JH, Kim SC, Shim IK, Song KB, Lee JH, Hwang DW, et al. Factors affecting the development of diabetes mellitus after pancreatic resection. Pancreas. 2015;44(8):1296-303.  201. Liao WC, Tu YK, Wu MS, Lin JT, Wang HP, Chien KL. Blood glucose concentration and risk of pancreatic cancer: Systematic review and dose-response meta-analysis. BMJ (Online). 2015;349((Liao W.-C.; Wu M.-S.; Wang H.-P.; Chien K.-L., klchien@ntu.edu.tw) Department of Internal Medicine, National Taiwan University Hospital, National Taiwan University College of Medicine, 7 Chang Shan South Road, Taipei, Taiwan).  202. Malle EK, Zammit NW, Walters SN, Koay YC, Wu J, Tan BM, et al. Nuclear factor κB-inducing kinase activation as a mechanism of pancreatic β cell failure in obesity. Journal of Experimental Medicine. 2015;212(8):1239-54.  203. Memon AA, Bennet L, Zöller B, Wang X, Palmer K, Sundquist K, et al. Circulating human epidermal growth factor receptor 2 (HER2) is associated with hyperglycaemia and insulin resistance. Journal of Diabetes. 2015;7(3):369-77.  204. Millar SR, Perry IJ, Phillips CM. HbA<inf>1c</inf> alone is a poor indicator of cardiometabolic risk in middle-aged subjects with pre-diabetes but is suitable for Type 2 diabetes diagnosis: A cross-sectional study. PLoS ONE. 2015;10(8).  205. Pfeifer CD, Schoennagel BP, Grosse R, Wang ZJ, Graessner J, Nielsen P, et al. Pancreatic iron and fat assessment by MRI-R2∗ in patients with iron overload diseases. Journal of Magnetic Resonance Imaging. 2015;42(1):196-203.  206. Shimodaira M, Niwa T, Nakajima K, Kobayashi M, Hanyu N, Nakayama T. The relation between CA 19-9 level and early-phase insulin secretion in normoglycemic and prediabetic subjects. International Journal of Biological Markers. 2015;30(2):e169-e73.  207. Sogabe M, Okahisa T, Taniguchi T, Tomonari T, Tanaka T, Tanaka H, et al. Light alcohol consumption plays a protective role against non-alcoholic fatty liver disease in Japanese men with metabolic syndrome. Liver International. 2015;35(6):1707-14.  208. Uygun A, Kadayifci A, Demirci H, Saglam M, Sakin YS, Ozturk K, et al. The effect of fatty pancreas on serum glucose parameters in patients with nonalcoholic steatohepatitis. European Journal of Internal Medicine. 2015;26(1):37-41.  209. Vrkljan AM, Grašić D, Kruljac I, Nikolić M, Filipović-Čugura J, Ulamec M, et al. Gastriccarcinoid type 1 in a patient with autoimmune polyglandular syndrome: Additional endocrinological evaluation required. Acta Clinica Croatica. 2015;54(4):525-9.  210. Breitling LP. Evidence of non-linearity in the association of glycemic control with influenza/pneumonia mortality: A study of 19 000 adults from the US general population. Diabetes/Metabolism Research and Reviews. 2016;32(1):111-20.  211. Busek P, Vanickova Z, Hrabal P, Brabec M, Fric P, Zavoral M, et al. Increased tissue and circulating levels of dipeptidyl peptidase-IV enzymatic activity in patients with pancreatic ductal adenocarcinoma. Pancreatology. 2016;16(5):829-38.  212. Cheng L, Qin T, Ma J, Duan W, Xu Q, Li X, et al. Hypoxia-inducible factor-1α mediates hyperglycemia-induced pancreatic cancer glycolysis. Anti-Cancer Agents in Medicinal Chemistry. 2016;19(12):1503-12.  213. Duksal T, Tiftikcioglu BI, Bilgin S, Kose S, Zorlu Y. Role of inflammation in sensory neuropathy in prediabetes or diabetes. Acta Neurologica Scandinavica. 2016;133(5):384-90.  214. Ferroni P, Riondino S, Laudisi A, Portarena I, Formica V, Alessandroni J, et al. Pretreatment insulin levels as a prognostic factor for breast cancer progression. Oncologist. 2016;21(9):1041-9.  215. Fu DX, Cui HB, Guo NN, Su N, Xu JX, Wang GY. Prediabetes and the risk of pancreatic cancer: A meta-analysis. International Journal of Clinical and Experimental Medicine. 2016;9(10):19474-9.  216. Gillies N, Pendharkar SA, Asrani VM, Mathew J, Windsor JA, Petrov MS. Interleukin-6 is associated with chronic hyperglycemia and insulin resistance in patients after acute pancreatitis. Pancreatology. 2016;16(5):748-55.  217. Hemmingsen B, Krogh J, Metzendorf MI, Richter B. Dipeptidyl-peptidase (DPP)-4 inhibitors or glucagon-like peptide (GLP)-1 analogues for prevention or delay of type 2 diabetes mellitus and its associated complications in persons at increased risk for the development of type 2 diabetes mellitus. Cochrane Database of Systematic Reviews. 2016;2016(5).  218. Hillson R. Pancreatitis, pancreatic cancer, and diabetes. Practical Diabetes. 2016;33(3):77-8.  219. Iavazzo C, McComiskey M, Datta M, Ryan M, Kiernan J, Winter-Roach B, et al. Preoperative HBA1c and risk of postoperative complications in patients with gynaecological cancer. Archives of Gynecology and Obstetrics. 2016;294(1):161-4.  220. Iizuka K, Fujisawa T, Takeda J. Concurrent insulinoma and impaired glucose tolerance suspected as owing to obesity. BMJ Case Reports. 2016;2016((Iizuka K., kiizuka@gifu-u.ac.jp; Fujisawa T.; Takeda J.) Diabetes and Endocrinology, Graduate School of Medicine, Gifu University, Gifu, Japan).  221. Jonas C, Maiter D, Alexopoulou O. Evolution of Glucose Tolerance after Treatment of Acromegaly: A Study in 57 Patients. Hormone and Metabolic Research. 2016;48(5):299-305.  222. Kang JS, Jang JY, Kang MJ, Kim E, Jung W, Chang J, et al. Endocrine Function Impairment After Distal Pancreatectomy: Incidence and Related Factors. World journal of surgery. 2016;40(2):440-6.  223. Li W, Zhang L, Chen X, Jiang Z, Zong L, Ma Q. Hyperglycemia Promotes the Epithelial-Mesenchymal Transition of Pancreatic Cancer via Hydrogen Peroxide. Oxidative Medicine and Cellular Longevity. 2016;2016((Li W., li.wei.1985@stu.xjtu.edu.cn; Zhang L., zhanglun3781@stu.xjtu.edu.cn; Chen X., 892241553@qq.com; Jiang Z., 806764362@qq.com; Zong L., zong_liang@sohu.com; Ma Q., qyma56@mail.xjtu.edu.cn) Department of Hepatobiliary Surgery, First Affiliated Hospital of Xi'an Jiaotong University, Xi'an, China).  224. Murakami R, Kawai T, Meguro S, Hayashi M, Itoh H. A case of autoimmune polyendocrine syndrome type I with strong positive GAD antibody titer, followed up with glucose tolerance measured by oral glucose tolerance test. Neuroendocrinology Letters. 2016;37(8):540-2.  225. Nakajima K, Suwa K. Association between positive fecal occult blood test and diabetes in a population undergoing health screening. Clinical Biochemistry. 2016((Nakajima K., nakajima-rsh@kuhs.ac.jp) School of Nutrition and Dietetics, Faculty of Health and Social Services, Kanagawa University of Human Services, 1-10-1 Heisei-cho, Yokosuka, Kanagawa 238-8522, Japan).  226. Pereira JPT, Romão V, Eulálio M, Jorge R, Breda F, Calretas S, et al. Sclerosing mesenteritis and disturbance of glucose metabolism: A new relationship? A case series. American Journal of Case Reports. 2016;17((Pereira J.P.T., joaoptavares85@gmail.com; Romão V.; Eulálio M.; Jorge R.; Breda F.; Calretas S.; Leitão S.; Santos R.; Carvalho A.) Department of Internal Medicine, Centro Hospitalar e Universitário de Coimbra, Coimbra, Portugal):55-9.  227. Rhee EJ, Park SE, Chang Y, Ryu S, Lee WY. Baseline glycemic status and mortality in 241,499 Korean metropolitan subjects: A Kangbuk Samsung Health Study. Metabolism: Clinical and Experimental. 2016;65(2):68-77.  228. Roeyen G, Jansen M, Chapelle T, Bracke B, Hartman V, Ysebaert D, et al. Diabetes mellitus and pre-diabetes are frequently undiagnosed and underreported in patients referred for pancreatic surgery. A prospective observational study. Pancreatology. 2016;16(4):671-6.  229. Saito H, Kashiyama H, Murohashi T, Sasaki K, Misawa R, Ohwada S. Case of Six-Year Disease-Free Survival with Undifferentiated Carcinoma of the Pancreas. Case Reports in Gastroenterology. 2016;10(2):472-8.  230. Sakaguchi K, Takeda K, Maeda M, Ogawa W, Sato T, Okada S, et al. Glucose area under the curve during oral glucose tolerance test as an index of glucose intolerance. Diabetology International. 2016;7(1):53-8.  231. Škrha P, Hořínek A, Pazourková E, Hajer J, Frič P, Škrha J, et al. Serum microRNA-196 and microRNA-200 in pancreatic ductal adenocarcinoma of patients with diabetes mellitus. Pancreatology. 2016;16(5):839-43.  232. Stewart SL, Dang J, Chen MS. Diabetes Prevalence and Risk Factors in Four Asian American Communities. Journal of community health. 2016;41(6):1264-73.  233. Sun ZJ, Yang YC, Wu JS, Wang MC, Chang CJ, Lu FH. Increased risk of glomerular hyperfiltration in subjects with impaired glucose tolerance and newly diagnosed diabetes. Nephrology Dialysis Transplantation. 2016;31(8):1295-301.  234. Susanto H, Liu TY, Chen CC, Purnomo JD, Chen SF, Wang CH. Increased serum levels of betatrophin in pancreatic cancer-associated diabetes. Oncotarget. 2016;7(27):42330-9.  235. Wallbach M, Duque Escobar J, Babaeikelishomi R, Stahnke MJ, Blume R, Schröder S, et al. Distinct functions of the dual leucine zipper kinase depending on its subcellular localization. Cellular Signalling. 2016;28(4):272-83.  236. Wilding JPH, Overgaard RV, Jacobsen LV, Jensen CB, Le Roux CW. Exposure-response analyses of liraglutide 3.0mg for weight management. Diabetes, Obesity and Metabolism. 2016;18(5):491-9.  237. Zhong GC, Ye MX, Cheng JH, Zhao Y, Gong JP. HbA1c and Risks of All-Cause and Cause-Specific Death in Subjects without Known Diabetes: A Dose-Response Meta-Analysis of Prospective Cohort Studies. Scientific reports. 2016;6((Zhong G.-C.; Ye M.-X.; Gong J.-P.) Department of Hepatobiliary Surgery, The Second Affiliated Hospital of Chongqing Medical University, Chongqing 400010, China):24071.  238. Zhou M, Zhu L, Cui X, Feng L, Zhao X, He S, et al. Influence of diet on leukocyte telomere length, markers of inflammation and oxidative stress in individuals with varied glucose tolerance: A Chinese population study. Nutrition Journal. 2016;15(1).  239. Akour A, Kasabri V, Bulatova N, Muhaissen SA, Naffa R, Fahmawi H, et al. Association of oxytocin with glucose intolerance and inflammation biomarkers in metabolic syndrome patients with and without prediabetes. Review of Diabetic Studies. 2017;14(4):364-71.  240. Arthur R, Rohrmann S, Møller H, Selvin E, Dobs AS, Kanarek N, et al. Pre-diabetes and serum sex steroid hormones among US men. Andrology. 2017;5(1):49-57.  241. Baye E, Menon K, De Courten MP, Earnest A, Cameron J, De Courten B. Does supplementation with carnosine improve cardiometabolic health and cognitive function in patients with pre-diabetes and type 2 diabetes? study protocol for a randomised, double-blind, placebo-controlled trial. BMJ Open. 2017;7(9).  242. Cansu GB, Atılgan S, Balcı MK, Sarı R, Özdem S, Altunbaş HA. Which type 2 diabetes mellitus patients should be screened for subclinical Cushing’s syndrome? Hormones. 2017;16(1):22-32.  243. Chen CC, Liu K, Hsu CC, Chang HY, Chung HC, Liu JS, et al. Healthy lifestyle and normal waist circumference are associated with a lower 5-year risk of type 2 diabetes in middle-aged and elderly individuals: Results from the healthy aging longitudinal study in Taiwan (HALST). Medicine (United States). 2017;96(6).  244. Chow WH, Chrisman M, Daniel CR, Ye Y, Gomez H, Dong Q, et al. Cohort profile: The Mexican American Mano a Mano Cohort. International Journal of Epidemiology. 2017;46(2).  245. Eaglehouse YL, Venditti EM, Kramer MK, Arena VC, Vanderwood KK, Rockette-Wagner B, et al. Factors related to lifestyle goal achievement in a diabetes prevention program dissemination study. Translational Behavioral Medicine. 2017;7(4):873-80.  246. El-Sayed SAM, Gashgary RA. Role of HA1c in early detections of prediabetic children. International Journal of Pharma and Bio Sciences. 2017;8(4):B526-B31.  247. Gillies NA, Pendharkar SA, Singh RG, Windsor JA, Bhatia M, Petrov MS. Fasting levels of insulin and amylin after acute pancreatitis are associated with pro-inflammatory cytokines. Archives of physiology and biochemistry. 2017;123(4):238-48.  248. Guadagni F, RionDino S, Formica V, Del Monte G, Morelli AM, Lucchetti J, et al. Clinical significance of glycemic parameters on venous thromboembolism risk preDiction in gastrointestinal cancer. World Journal of Gastroenterology. 2017;23(28):5187-95.  249. Hopkins L, Brown-Broderick J, Hearn J, Malcolm J, Chan J, Hicks-Boucher W, et al. Implementation of a referral to discharge glycemic control initiative for reduction of surgical site infections in gynecologic oncology patients. Gynecologic Oncology. 2017;146(2):228-33.  250. Hwang HK, Park J, Choi SH, Kang CM, Lee WJ. Predicting new-onset diabetes after minimally invasive subtotal distal pancreatectomy in benign and borderline malignant lesions of the pancreas. Medicine (United States). 2017;96(51).  251. Inaba H, Funahashi T, Ariyasu H, Iwakura H, Furuta H, Nishi M, et al. Diabetic ketoacidosis in a patient with acromegaly and central diabetes insipidus treated with octreotide long-acting release. Diabetology International. 2017;8(2):237-42.  252. Jia Y, Luo X, Ji Y, Xie J, Jiang H, Fu M, et al. Circulating CTRP9 levels are increased in patients with newly diagnosed type 2 diabetes and correlated with insulin resistance. Diabetes Research and Clinical Practice. 2017;131((Jia Y.; Ji Y.; Xie J.; Jiang H.; Li X., lxqh1977@163.com) Ministry of Education Key Laboratory of Child Development and Disorders, Key Laboratory of Pediatrics in Chongqing, Chongqing International Science and Technology Cooperation Center for Child Development and Disorders, Children's Hospital of Chongqing Medical University, Chongqing, China):116-23.  253. Jian-Yu E, Lu SE, Lin Y, Graber JM, Rotter D, Zhang L, et al. Differential and Joint effects of metformin and statins on overall survival of elderly patients with pancreatic adenocarcinoma: A large population-based study. Cancer Epidemiology Biomarkers and Prevention. 2017;26(8):1225-32.  254. Li Y, Ran W, Zhang J, Chen S, Luo D, Wang C, et al. Elevated serum milk fat globule-epidermal growth factor 8 levels in type 2 diabetic patients are suppressed by overweight or obese status. IUBMB Life. 2017;69(2):63-71.  255. Roeyen G, Jansen M, Hartman V, Chapelle T, Bracke B, Ysebaert D, et al. The impact of pancreaticoduodenectomy on endocrine and exocrine pancreatic function: A prospective cohort study based on pre- and postoperative function tests. Pancreatology. 2017;17(6):974-82.  256. Saad EA, Habib SA, Refai WA, Elfayoumy AA. Malondialdehyde, adiponectin, nitric oxide, C-reactive protein, tumor necrosis factor-alpha and insulin resistance relationships and interrelationships in type 2 diabetes early stage. Is metformin alone adequate in this stage? International Journal of Pharmacy and Pharmaceutical Sciences. 2017;9(10):176-81.  257. Shetty N, Thomas A, Cutinha FP, Rai P, Thamizholi S, Ashraf K. Association of diabetes and cancer: An analysis on the prevalence of diabetes among cancer patients. Research Journal of Pharmaceutical, Biological and Chemical Sciences. 2017;8(6):235-41.  258. Škrha J, Bušek P, Uhrová J, Hrabal P, Kmochová K, Laclav M, et al. Lower plasma levels of glucose-dependent insulinotropic peptide (GIP) and pancreatic polypeptide (PP) in patients with ductal adenocarcinoma of the pancreas and their relation to the presence of impaired glucoregulation and weight loss. Pancreatology. 2017;17(1):89-94.  259. Su X, He X, Ben Q, Wang W, Song H, Ye Q, et al. Effect of p53 on pancreatic cancer-glucose tolerance abnormalities by regulating transglutaminase 2 in resistance to glucose metabolic stress. Oncotarget. 2017;8(43):74299-311.  260. Thongsroy J, Patchsung M, Mutirangura A. The association between Alu hypomethylation and severity of type 2 diabetes mellitus. Clinical Epigenetics. 2017;9(1).  261. Vahid F, Shivappa N, Karamati M, Naeini AJ, Hebert JR, Davoodi SH. Association between Dietary Inflammatory Index (DII) and risk of prediabetes: a case-control study. Applied physiology, nutrition, and metabolism = Physiologie appliquee, nutrition et metabolisme. 2017;42(4):399-404.  262. Van Laecke S, Caluwe R, Huybrechts I, Nagler EV, Vanholder R, Peeters P, et al. Effect of magnesium supplements on insulin secretion after kidney transplantation: A randomized controlled trial. Annals of Transplantation. 2017;22((Van Laecke S., steven.vanlaecke@ugent.be; Nagler E.V.; Vanholder R.; Peeters P.; Van Biesen W.) Renal Division, Department of Internal Medicine, Ghent University Hospital, Ghent, Belgium):524-31.  263. Wróbel MP, Marek B, Kajdaniuk D, Rokicka D, Szymborska-Kajanek A, Strojek K. Metformin - A new old drug. Endokrynologia Polska. 2017;68(4):482-96.  264. Abedini A, Cao P, Plesner A, Zhang J, He M, Derk J, et al. RAGE binds preamyloid IAPP intermediates and mediates pancreatic β cell proteotoxicity. Journal of Clinical Investigation. 2018;128(2):682-98.  265. Abu Bakar MH, Hairunisa N, Zaman Huri H. Reduced mitochondrial DNA content in lymphocytes is associated with insulin resistance and inflammation in patients with impaired fasting glucose. Clinical and Experimental Medicine. 2018;18(3):373-82.  266. Alkandari A, Longenecker JC, Barengo NC, Alkhatib A, Weiderpass E, Al-Wotayan R, et al. The prevalence of pre-diabetes and diabetes in the Kuwaiti adult population in 2014. Diabetes Research and Clinical Practice. 2018;144((Alkandari A., abdullah.alkandari@dasmaninstitute.org; Alkhatib A.; Weiderpass E.; Al Duwairi Q.; Tuomilehto J.) Dasman Diabetes Institute, Kuwait City, Kuwait):213-23.  267. Alsalhi MS, Devanesan S, Alzahrani KE, Alshebly M, Al-Qahtani F, Farhat K, et al. Impact of diabetes mellitus on human erythrocytes: Atomic force microscopy and spectral investigations. International Journal of Environmental Research and Public Health. 2018;15(11).  268. Bulatova N, Kasabri V, Qotineh A, Al-Athami T, Yousef AM, AbuRuz S, et al. Effect of metformin combined with lifestyle modification versus lifestyle modification alone on proinflammatory-oxidative status in drug-naïve pre-diabetic and diabetic patients: A randomized controlled study. Diabetes and Metabolic Syndrome: Clinical Research and Reviews. 2018;12(3):257-67.  269. Dittus KL, Harvey JR, Bunn JY, Kokinda ND, Wilson KM, Priest J, et al. Impact of a behaviorally-based weight loss intervention on parameters of insulin resistance in breast cancer survivors. BMC Cancer. 2018;18(1).  270. Harding JJ, Do RK, Dika IE, Hollywood E, Uhlitskykh K, Valentino E, et al. A phase 1 study of ADI-PEG 20 and modified FOLFOX6 in patients with advanced hepatocellular carcinoma and other gastrointestinal malignancies. Cancer Chemotherapy and Pharmacology. 2018;82(3):429-40.  271. Iacovazzo D, Flanagan SE, Walker E, Quezado R, De Sousa Barros FA, Caswell R, et al. MAFA missense mutation causes familial insulinomatosis and diabetes mellitus. Proceedings of the National Academy of Sciences of the United States of America. 2018;115(5):1027-32.  272. Iwayama H, Hirase S, Nomura Y, Ito T, Morita H, Otake K, et al. Spontaneous adrenocorticotropic hormone (ACTH) normalisation due to tumour regression induced by metyrapone in a patient with ectopic ACTH syndrome: Case report and literature review. BMC Endocrine Disorders. 2018;18(1).  273. Joshipura KJ, Muñoz-Torres FJ, Dye BA, Leroux BG, Ramírez-Vick M, Pérez CM. Longitudinal association between periodontitis and development of diabetes. Diabetes Research and Clinical Practice. 2018;141((Joshipura K.J., kaumudi.joshipura@upr.edu; Muñoz-Torres F.J.) Center for Clinical Research and Health Promotion, University of Puerto Rico Medical Sciences Campus, San Juan, Puerto Rico):284-93.  274. Keskin M, Tekin Ö, Or Koca A, Dağdeviren M, Altay M, Ertuğrul DT. Lichen Sclerosis et Atrophicus May be Associated with Prediabetes. Turkish Journal of Endocrinology and Metabolism. 2018;22(2):S21-S.  275. Mészáros G, Pasquier A, Vivot K, Goginashvili A, Ricci R. Lysosomes in nutrient signalling: A focus on pancreatic β-cells. Diabetes, Obesity and Metabolism. 2018;20((Mészáros G.; Pasquier A.; Vivot K.; Goginashvili A., agoginashvili@ucsd.edu; Ricci R., ricci@igbmc.fr) Institut de Génétique et de Biologie Moléculaire et Cellulaire, Illkirch, France):104-15.  276. Moxley EW, Smith D, Quinn L, Park C. Relationships Between Glycemic Control and Cardiovascular Fitness. Biological research for nursing. 2018;20(4):422-8.  277. Ozturk K, Dogan T, Celikkanat S, Ozen A, Demirci H, Kurt O, et al. The association of fatty pancreas with subclinical atherosclerosis in nonalcoholic fatty liver disease. European Journal of Gastroenterology and Hepatology. 2018;30(4):411-7.  278. Peterson CM, Beyl RA, Marlatt KL, Martin CK, Aryana KJ, Marco ML, et al. Effect of 12 wk of resistant starch supplementation on cardiometabolic risk factors in adults with prediabetes: A randomized controlled trial. American Journal of Clinical Nutrition. 2018;108(3):492-501.  279. Schofield HK, Tandon M, Park MJ, Halbrook CJ, Ramakrishnan SK, Kim EC, et al. Pancreatic HIF2α Stabilization Leads to Chronic Pancreatitis and Predisposes to Mucinous Cystic Neoplasm. Cellular and Molecular Gastroenterology and Hepatology. 2018;5(2):169-85.e2.  280. Simeone P, Liani R, Tripaldi R, Di Castelnuovo A, Guagnano MT, Tartaro A, et al. Thromboxane-dependent platelet activation in obese subjects with prediabetes or early type 2 diabetes: Effects of liraglutide-or lifestyle changes-induced weight loss. Nutrients. 2018;10(12).  281. Singh AN, Pal S, Kilambi R, Madhusudhan KS, Dash NR, Tandon N, et al. Diabetes after pancreaticoduodenectomy: can we predict it? Journal of Surgical Research. 2018;227((Singh A.N., anandnsingh@gmail.com; Pal S.; Kilambi R.; Dash N.R.; Sahni P.) Department of Gastrointestinal Surgery and Liver Transplantation, All India Institute of Medical Sciences, New Delhi, India):211-9.  282. Song R, Cao S. Prediabetes Directly Deteriorates into Diabetic Ketoacidosis and Hyperosmolar Hyperglycemic Syndrome Triggered by Acute Pancreatitis: A Case Report Illustrating a “Chicken and Egg” Paradigm in Ketosis-Prone Diabetes. Diabetes Therapy. 2018;9(3):1377-83.  283. Thomson H, Oliver N, Godsland IF, Darzi A, Srivanichakorn W, Majeed A, et al. Protocol for a clinical trial of text messaging in addition to standard care versus standard care alone in prevention of type 2 diabetes through lifestyle modification in India and the UK. BMC endocrine disorders. 2018;18(1):63.  284. Wang M, Tang X, Li L, Liu D, Liu H, Zheng H, et al. C1q/TNF-related protein-6 is associated with insulin resistance and the development of diabetes in Chinese population. Acta Diabetologica. 2018;55(12):1221-9.  285. Werner C, Lupp A, Drescher R, Freesmeyer M, Mireskandari M, Stoykow C, et al. Morphologically ‘invisible’ proinsulin – secreting adenoma detected by Ga-68 Exendin-4 (GLP-1 Receptor) positron emission tomography/CT. Journal of Medical Imaging and Radiation Oncology. 2018;62(3):370-4.  286. Zeng J, Xu Y, Shi Y, Jiang C. Inflammation role in sensory neuropathy in Chinese patients with diabetes/prediabetes. Clinical Neurology and Neurosurgery. 2018;166((Zeng J., jtnfft@163.com) Department of Neurology, Zhongshan Traditional Chinese Medicine Hospital, Zhongshan, China):136-40.  287. Zhang Y, Huang S, Li P, Chen Q, Li Y, Zhou Y, et al. Pancreatic cancer-derived exosomes suppress the production of GIP and GLP-1 from STC-1cells in vitro by down-regulating the PCSK1/3. Cancer Letters. 2018;431((Zhang Y.; Huang S.; Li P.; Chen Q.; Li Y.; Zhou Y.; Wang L.; Kang M.; Zhang B.; Yang B.; Wu Y., yulianwu@zju.edu.cn) Department of Surgery, The Second Affiliated Hospital, Zhejiang University School of Medicine, Hangzhou, Zhejiang, China):190-200.  288. Zhang Y, Xing Y, Yuan H, Gang X, Guo W, Li Z, et al. Impaired glucose metabolisms of patients with obstructive sleep apnea and type 2 diabetes. Journal of Diabetes Research. 2018;2018((Zhang Y.; Gang X.; Guo W.; Li Z., zhuoli@jlu.edu.cn; Wang G., gwang168@jlu.edu.cn) Department of Endocrinology and Metabolism, First Hospital of Jilin University, Changchun, Jilin, China).  289. Appelbaum R, Alvarado FJ, Blackham AU, Brodsky J. A case of acute pancreatitis associated with early-stage adenocarcinoma of the gallbladder. American Journal of Case Reports. 2019;20((Appelbaum R., rachel.appelbaum@lvhn.org; Alvarado F.J.) Department of Surgery, Lehigh Valley Health Network, Allentown, PA, United States):957-60.  290. Ares J, Valdés S, Botas P, Sánchez-Ragnarsson C, Rodríguez-Rodero S, Morales-Sánchez P, et al. Mortality risk in adults according to categories of impaired glucose metabolism after 18 years of follow-up in the North of Spain: The Asturias study. PLoS ONE. 2019;14(1).  291. Baldwin C, Zerofsky M, Sathe M, Troendle DM, Perito ER. Acute Recurrent and Chronic Pancreatitis as Initial Manifestations of Cystic Fibrosis and Cystic Fibrosis Transmembrane Conductance Regulator-Related Disorders. Pancreas. 2019;48(7):888-93.  292. Bharmal SH, Pendharkar S, Singh RG, Cho J, Petrov MS. Glucose Counter-regulation after Acute Pancreatitis. Pancreas. 2019;48(5):670-81.  293. Cai Z, Liang Y, Xing C, Wang H, Hu P, Li J, et al. Cancer‑associated adipocytes exhibit distinct phenotypes and facilitate tumor progression in pancreatic cancer. Oncology Reports. 2019;42(6):2537-49.  294. De Ritter R, Sep SJS, Van Der Kallen CJH, Schram MT, Koster A, Kroon AA, et al. Adverse differences in cardiometabolic risk factor levels between individuals with pre-diabetes and normal glucose metabolism are more pronounced in women than in men: The Maastricht Study. BMJ Open Diabetes Research and Care. 2019;7(1).  295. Dobri G, Niwattisaiwong S, Bena JF, Gupta M, Kirwan J, Kennedy L, et al. Is GH nadir during OGTT a reliable test for diagnosis of acromegaly in patients with abnormal glucose metabolism? Endocrine. 2019;64(1):139-46.  296. Dutta P, Hajela A, Gupta P, Rai A, Sachdeva N, Mukherjee KK, et al. The predictors of recovery from diabetes mellitus following neurosurgical treatment of acromegaly: A prospective study over a decade. Neurology India. 2019;67(3):757-62.  297. El-Ashmawy HM, Selim FO, Hosny TAM, Almassry HN. Association of low serum Meteorin like (Metrnl) concentrations with worsening of glucose tolerance, impaired endothelial function and atherosclerosis. Diabetes Research and Clinical Practice. 2019;150((El-Ashmawy H.M., hazemashmawy@zu.edu.eg; Selim F.O.) Department of Internal Medicine, Zagazig University, Faculty of Medicine, Egypt):57-63.  298. Friedrich K, Sommer M, Strobel S, Thrum S, Blüher M, Wagner U, et al. Perturbation of the monocyte compartment in human obesity. Frontiers in Immunology. 2019;10(AUG).  299. Gröber U, Holick MF. Diabetes prevention: Vitamin D supplementation may not provide any protection if there is no evidence of deficiency! Nutrients. 2019;11(11).  300. Hasan A, Kochumon S, Al-Ozairi E, Tuomilehto J, Ahmad R. Association between adipose tissue interleukin-33 and immunometabolic markers in individuals with varying degrees of glycemia. Disease Markers. 2019;2019((Hasan A., amal.hasan@dasmaninstitute.org; Kochumon S., shihab.kochumon@dasmaninstitute.org; Tuomilehto J., jaakko.tuomilehto@thl.fi; Ahmad R., rasheed.ahmad@dasmaninstitute.org) Research Division, Dasman Diabetes Institute, Dasman, Kuwait).  301. Hasan Z, Irfan M, Masood Q, Ahmed O, Moosajee US, Rao S, et al. Raised levels of IFN-gamma and IL-13 are associated with pre-diabetes amongst newly diagnosed patients with tuberculosis. Journal of the Pakistan Medical Association. 2019;69(4):468-73.  302. He W, Yan L, Wang M, Li Q, He M, Ma Z, et al. Surgical outcomes and predictors of glucose metabolism alterations for growth hormone-secreting pituitary adenomas: a hospital-based study of 151 cases. Endocrine. 2019;63(1):27-35.  303. Hu W, Fan X, Zhou B, Li L, Tian B, Fang X, et al. Circulating alarin concentrations are high in patients with type 2 diabetes and increased by glucagon-like peptide-1 receptor agonist treatment: An Consort-compliant study. Medicine (United States). 2019;98(28).  304. Ilie MD, Raverot V, Tronc F, Vasiljevic A, Borson-Chazot F, Raverot G. Cabergoline in severe ectopic or occult Cushing's syndrome. European Journal of Endocrinology. 2019;181(1):K1-K9.  305. Lee CMY, Colagiuri S, Woodward M, Gregg EW, Adams R, Azizi F, et al. Comparing different definitions of prediabetes with subsequent risk of diabetes: An individual participant data meta-analysis involving 76 513 individuals and 8208 cases of incident diabetes. BMJ Open Diabetes Research and Care. 2019;7(1).  306. Lee JE, Oh TJ, Moon JH, Park KS, Jang HC, Choi SH. Serum neopterin concentration and impaired glucose metabolism: Relationship with β-cell function and insulin resistance. Frontiers in Endocrinology. 2019;10(FEB).  307. Li Y, Deng S, Peng J, Wang X, Essandoh K, Mu X, et al. MicroRNA-223 is essential for maintaining functional β-cell mass during diabetes through inhibiting both FOXO1 and SOX6 pathways. Journal of Biological Chemistry. 2019;294(27):10438-48.  308. Maschio DA, Matheus VA, Collares-Buzato CB. Islet cells are the source of Wnts that can induce beta-cell proliferation in vitro. Journal of Cellular Physiology. 2019;234(11):19852-65.  309. Murtola TJ, Sälli SM, Talala K, Taari K, Tammela TLJ, Auvinen A. Blood glucose, glucose balance, and disease-specific survival after prostate cancer diagnosis in the Finnish Randomized Study of Screening for Prostate Cancer. Prostate Cancer and Prostatic Diseases. 2019;22(3):453-60.  310. Nomoto H, Gurlo T, Rosenberger M, Girgis MD, Dry S, Butler PC. Low Grade Islet but Marked Exocrine Pancreas Inflammation in an Adult with Autoimmune Pre-Diabetes. Case Reports in Endocrinology. 2019;2019((Nomoto H., hnomoto@med.hokudai.ac.jp; Gurlo T., tgurlo@mednet.ucla.edu; Rosenberger M., mrosenberger@mednet.ucla.edu; Butler P.C., pbutler@mednet.ucla.edu) David Geffen School of Medicine at UCLA, Larry L. Hillblom Islet Research Center, Los Angeles, CA, United States).  311. Slade AN. Prostate cancer and subsequent nutritional outcomes: the role of diagnosis and treatment. Journal of cancer survivorship : research and practice. 2019;13(2):171-9.  312. Tavares AM, Silva JH, de Oliveira Bensusan C, Ferreira ACF, de Lima Matos LP, de Araujo e Souza KL, et al. Altered superoxide dismutase-1 activity and intercellular adhesion molecule 1 (ICAM-1) levels in patients with type 2 diabetes mellitus. PLoS ONE. 2019;14(5).  313. Umemoto K, Tsuchikawa T, Nakamura T, Okamura K, Noji T, Asano T, et al. Postoperative nutritional benefits of proximal parenchymal pancreatectomy for low-grade malignant lesions in the pancreatic head. HPB. 2019;21(11):1491-6.  314. Vigneri R, Sciacca L. Diabetes and Cancer. 2019. p. 1-34.  315. Wang Z, Gao L, Guo X, Feng C, Deng K, Lian W, et al. Preoperative Fasting C-Peptide Acts as a Promising Predictor of Improved Glucose Tolerance in Patients With Acromegaly After Transsphenoidal Surgery: A Retrospective Study of 64 Cases From a Large Pituitary Center in China. Frontiers in Endocrinology. 2019;10((Wang Z.; Gao L.; Guo X.; Feng C.; Deng K.; Lian W.; Feng M.; Bao X.; Xing B., xingbingemail@aliyun.com) Department of Neurosurgery, Peking Union Medical College Hospital, Chinese Academy of Medical Sciences and Peking Union Medical College, Beijing, China).  316. Zhang M, Chua MS, Hu J, Li H, Zhang S, Wu L, et al. High inflammatory factor grading predicts poor disease-free survival in AJCC stage I-II hepatocellular carcinoma patients after R0 resection. Cancer Management and Research. 2019;11((Zhang M.; Li H.; Zhang S.; Wu L., wulq581031@163.com; Han B., wfkevinhan@163.com) Department of Hepatobiliary and Pancreatic Surgery, The Affiliated Hospital of Qingdao University, Qingdao, Shandong, China):10623-32.  317. Zhong JW, Yu LM, Ying QJ, Peng WW, Xu CZ, Cai ZZ, et al. Performance of endoscopic ultrasound for diagnosis of agenesis of the dorsal pancreas: a case report. Journal of International Medical Research. 2019;48(5).  318. Akabori H, Tani M, Kitamura N, Maehira H, Imashuku Y, Tsujita Y, et al. Perioperative tight glycemic control using artificial pancreas decreases infectious complications via suppression of inflammatory cytokines in patients who underwent pancreaticoduodenectomy: A prospective, non-randomized clinical trial. American Journal of Surgery. 2020;220(2):365-71.  319. Altaiee MS, Mohammed TU, Muhsin ADA. Evaluation of serum adropin levels in nonalcoholic fatty liver disease as a complication of hypothyroidism in Iraqi patients. International Journal of Pharmaceutical Research. 2020;12((Altaiee M.S., marwa.altaiee90@gmail.com; Mohammed T.U.) Department of Chemistry, College of Education for Pure Sciences (Ibn-AL-Haitham), Baghdad University, Iraq):2993-8.  320. Ayoub NM, Jaradat SK, Alhusban A, Tahaineh L. Glycosylated hemoglobin A1c is associated with anthropometric measurements and tumor characteristics in breast cancer patients. International Journal of Women's Health. 2020;12((Ayoub N.M., nmayoub@just.edu.jo; Jaradat S.K.; Alhusban A.; Tahaineh L.) Department of Clinical Pharmacy, Faculty of Pharmacy, Jordan University of Science and Technology (JUST, Irbid, Jordan):139-49.  321. Bar-Mashiah A, Aronson A, Naparst M, Dimaio CJ, Lucas AL. Elevated hemoglobin A1c is associated with the presence of pancreatic cysts in a high-risk pancreatic surveillance program. BMC Gastroenterology. 2020;20(1).  322. Bartlett DB, Slentz CA, Willis LH, Hoselton A, Huebner JL, Kraus VB, et al. Rejuvenation of Neutrophil Functions in Association With Reduced Diabetes Risk Following Ten Weeks of Low-Volume High Intensity Interval Walking in Older Adults With Prediabetes – A Pilot Study. Frontiers in Immunology. 2020;11((Bartlett D.B., david.bartlett@duke.edu; Slentz C.A.; Willis L.H.; Hoselton A.; Huebner J.L.; Kraus V.B.; Moss J.; Muehlbauer M.J.; Muoio D.M.; Koves T.R.; Wu H.; Huffman K.M.; Kraus W.E.) Duke Molecular Physiology Institute, School of Medicine, Duke University, Durham, NC, United States).  323. Bellafante E, McIlvride S, Nikolova V, Fan HM, Manna LB, Chambers J, et al. Maternal glucose homeostasis is impaired in mouse models of gestational cholestasis. Scientific reports. 2020;10(1):11523.  324. Chen YC, Mains RE, Eipper BA, Hoffman BG, Czyzyk TA, Pintar JE, et al. PAM haploinsufficiency does not accelerate the development of diet- and human IAPP-induced diabetes in mice. Diabetologia. 2020;63(3):561-76.  325. Chestnut C, Smelser W, Dum T, Bechtel M, Hand L, Michel C, et al. Glycemic impact of a diet and lifestyle intervention on diabetics and prediabetics during treatment for non-muscle invasive bladder cancer. Nutrition and Cancer. 2020;72(7):1219-24.  326. Hachiya H, Aoki T, Iso Y, Shimizu T, Tago K, Park KH, et al. Effects of branched-chain amino acids on postoperative tumor recurrence in patients undergoing curative resection for hepatocellular carcinoma: A randomized clinical trial. Journal of Hepato-Biliary-Pancreatic Sciences. 2020;27(11):819-29.  327. Hajiahmadi S, Nadjarzadeh A, Gharipour M, Hosseinzadeh M, Fallahzadeh H, Mohsenpour MA. Effect of flaxseed oil on glycemic control and inflammatory markers in overweight adults with pre-diabetes: A double-blind randomized controlled clinical trial. Journal of Herbal Medicine. 2020;24((Hajiahmadi S.; Mohsenpour M.A.) Department of Nutrition, International Campus, Shahid Sadughi University of Medical Sciences, Yazd, Iran).  328. Harada E, Morizono T, Kanno T, Saito M, Kawagishi H. Medicinal mushroom, grifola gargal (Agaricomycetes), lowers triglyceride in animal models of obesity and diabetes and in adults with prediabetes. International Journal of Medicinal Mushrooms. 2020;22(1):79-91.  329. Horii T, Fujita Y, Ishibashi C, Fukui K, Eguchi H, Kozawa J, et al. Islet inflammation is associated with pancreatic fatty infiltration and hyperglycemia in type 2 diabetes. BMJ Open Diabetes Research and Care. 2020;8(1).  330. Hu L, He F, Huang M, Zhao Q, Cheng L, Said N, et al. SPARC promotes insulin secretion through down-regulation of RGS4 protein in pancreatic β cells. Scientific reports. 2020;10(1):17581.  331. Jaghutriz BA, Wagner R, Heni M, Lehmann R, Machann J, Stefan N, et al. Metabolomic characteristics of fatty pancreas. Experimental and Clinical Endocrinology and Diabetes. 2020;128(12):804-10.  332. Kira S, Ito C, Fujikawa R, Misumi M. Increased cancer mortality among Japanese individuals with hyperinsulinemia. Metabolism Open. 2020;7((Kira S., skira.medical-court@uvgt-medical.com; Ito C.; Fujikawa R.) Grand Tower Medical Court, Hiroshima, Japan).  333. Kobayashi D, Kuriyama N, Noto H, Aida A, Takahashi O, Shimbo T. Development of malignancies and changes in time-dependent hemoglobin A1c among a nondiabetic population: longitudinal analysis. Acta Diabetologica. 2020;57(2):189-96.  334. Kuwabara-Ohmura Y, Iizuka K, Liu Y, Takao K, Nonomura K, Kato T, et al. A case of mody5-like manifestations without mutations or deletions in coding and minimal promoter regions of the hnf1b gene. Endocrine Journal. 2020;67(9):981-8.  335. Leong A, Lim VJY, Wang C, Chai JF, Dorajoo R, Heng CK, et al. Association of G6PD variants with hemoglobin A1c and impact on diabetes diagnosis in East Asian individuals. BMJ Open Diabetes Research and Care. 2020;8(1).  336. Li HL, Fang J, Zhao LG, Liu DK, Wang J, Han LH, et al. Personal Characteristics Effects on Validation of Self-reported Type 2 Diabetes From a Cross-sectional Survey Among Chinese Adults. Journal of epidemiology. 2020;30(11):516-21.  337. Liu Y, Zeng Y, Miao Y, Cheng X, Deng S, Hao X, et al. Relationships among pancreatic beta cell function, the Nrf2 pathway, and IRS2: a cross-sectional study. Postgraduate Medicine. 2020;132(8):720-6.  338. Lou B, Boger M, Bennewitz K, Sticht C, Kopf S, Morgenstern J, et al. Elevated 4-hydroxynonenal induces hyperglycaemia via Aldh3a1 loss in zebrafish and associates with diabetes progression in humans. Redox Biology. 2020;37((Lou B.; Boger M.; Bennewitz K.; Kroll J., jens.kroll@medma.uni-heidelberg.de) Department of Vascular Biology and Tumor Angiogenesis, European Center for Angioscience (ECAS), Medical Faculty Mannheim, Heidelberg University, Mannheim, Germany).  339. Maluf S, Salgado JV, Cysne DN, Camelo DMF, Nascimento JR, Maluf BVT, et al. Increased Glycated Hemoglobin Levels in Patients With Helicobacter pylori Infection Are Associated With the Grading of Chronic Gastritis. Frontiers in Immunology. 2020;11((Maluf S.; Nascimento J.R.; Silva L.D.M.; Belfort M.R.D.C.) Health Science Graduate Program, Federal University of Maranhão, São Luís, Brazil).  340. Nagpal SJS, Kandlakunta H, Her T, Sharma A, Sannapaneni S, Smyrk TC, et al. Pancreatic ductal adenocarcinoma is associated with a unique endocrinopathy distinct from type 2 diabetes mellitus. Pancreatology. 2020;20(5):929-35.  341. Nomoto H, Pei L, Montemurro C, Rosenberger M, Furterer A, Coppola G, et al. Activation of the HIF1α/PFKFB3 stress response pathway in beta cells in type 1 diabetes. Diabetologia. 2020;63(1):149-61.  342. Panozzo G, Staurenghi G, Dalla Mura G, Giannarelli D, Alessio G, Alongi S, et al. Prevalence of diabetes and diabetic macular edema in patients undergoing senile cataract surgery in Italy: The DIabetes and CATaract study. European Journal of Ophthalmology. 2020;30(2):315-20.  343. Petrák O, Klímová J, Mráz M, Haluzíková D, Dole alová RP, Kratochvílová H, et al. Pheochromocytoma with adrenergic biochemical phenotype shows decreased GLP-1 secretion and impaired glucose tolerance. Journal of Clinical Endocrinology and Metabolism. 2020;105(6):1878-87.  344. Petrák O, Klímová J, Mráz M, Haluzíková D, Doležalová RP, Kratochvílová H, et al. Pheochromocytoma with adrenergic biochemical phenotype shows decreased GLP-1 secretion and impaired glucose tolerance. Journal of Clinical Endocrinology and Metabolism. 2020;105(6):1878-87.  345. Sztromwasser P, Michalak A, Małachowska B, Młudzik P, Antosik K, Hogendorf A, et al. A cross-sectional study of patients referred for HNF1B-MODY genetic testing due to cystic kidneys and diabetes. Pediatric Diabetes. 2020;21(3):422-30.  346. Tariq M, Jajja MR, Maxwell DW, Galindo RJ, Sweeney JF, Sarmiento JM. Diabetes development after distal pancreatectomy: results of a 10 year series. HPB. 2020;22(7):1034-41.  347. Toshimitsu T, Gotou A, Sashihara T, Hachimura S, Shioya N, Suzuki S, et al. Effects of 12-week ingestion of yogurt containing lactobacillus plantarum OLL2712 on glucose metabolism and chronic inflammation in prediabetic adults: A randomized placebo-controlled trial. Nutrients. 2020;12(2).  348. Utzschneider KM, Johnson TN, Breymeyer KL, Bettcher L, Raftery D, Newton KM, et al. Small changes in glucose variability induced by low and high glycemic index diets are not associated with changes in β-cell function in adults with pre-diabetes. Journal of Diabetes and its Complications. 2020;34(8).  349. Wang T, Lu J, Shi L, Chen G, Xu M, Xu Y, et al. Association of insulin resistance and β-cell dysfunction with incident diabetes among adults in China: a nationwide, population-based, prospective cohort study. The Lancet Diabetes and Endocrinology. 2020;8(2):115-24.  350. Wu T, Hsu FC, Wang S, Luong D, Pierce JP. Hemoglobin a1c levels modify associations between dietary acid load and breast cancer recurrence. Nutrients. 2020;12(2).  351. Yip L, Fuhlbrigge R, Alkhataybeh R, Fathman CG. Gene Expression Analysis of the Pre-Diabetic Pancreas to Identify Pathogenic Mechanisms and Biomarkers of Type 1 Diabetes. Frontiers in Endocrinology. 2020;11((Yip L.; Fuhlbrigge R.; Alkhataybeh R.; Fathman C.G.) Division of Immunology and Rheumatology, Department of Medicine, Stanford University, Stanford, CA, United States).  352. Zhuge X, Wang Y, Chen X, Guo C. Diabetes in Patients With Pancreatic Neuroendocrine Neoplasms. Frontiers in Endocrinology. 2020;11((Zhuge X.) Department of Laboratory Medicine, The First Affiliated Hospital, Zhejiang University School of Medicine, Hangzhou, China).  353. Akirov A, Gorshtein A, Dotan I, Khazen NS, Pauker Y, Gershinsky M, et al. Long-term safety and efficacy of long-acting pasireotide in acromegaly. Endocrine. 2021;74(2):396-403.  354. Alkabab YMA, Biswas S, Ahmed S, Paul K, Nagajyothi J, Banu S, et al. Differentiating transient from persistent diabetic range hyperglycemia in a cohort of people completing tuberculosis treatment in Dhaka, Bangladesh. PLoS ONE. 2021;16(11 November).  355. Bharmal SH, Alarcon Ramos GC, Ko J, Petrov MS. Abdominal fat distribution modulates the metabolic effects of exogenous ketones in individuals with new-onset prediabetes after acute pancreatitis: Results from a randomized placebo-controlled trial. Clinical Nutrition ESPEN. 2021;43((Bharmal S.H.; Alarcon Ramos G.C.; Ko J.; Petrov M.S., max.petrov@gmail.com) School of Medicine, University of Auckland, Auckland, New Zealand):117-29.  356. Bian C, Gao J, Wang Y, Li J, Luan Z, Lu H, et al. Association of SIRT6 circulating levels with urinary and glycometabolic markers in pre-diabetes and diabetes. Acta Diabetologica. 2021;58(11):1551-62.  357. Bolasco G, Capriati T, Maggiore G, Diamanti A, Grimaldi C, Spada M, et al. Long-term outcome of pancreatic function following oncological surgery in children: Institutional experience and review of the literature. World Journal of Clinical Cases. 2021;9(25):7340-9.  358. Chabot K, Gillis C, Minnella EM, Ferreira V, Awasthi R, Baldini G, et al. Functional capacity of prediabetic patients: effect of multimodal prehabilitation in patients undergoing colorectal cancer resection. Acta Oncologica. 2021;60(8):1025-31.  359. Chang X, Wang Y, Fu S, Tang X, Liu J, Zhao N, et al. The detection of thyroid nodules in prediabetes population and analysis of related factors. Risk Management and Healthcare Policy. 2021;14((Chang X.; Wang Y.; Fu S., fusb@lzu.edu.cn; Tang X.; Liu J.; Zhao N.; Jing G.; Niu Q.; Ma L.) Department of Endocrinology, First Hospital of Lanzhou University, Gansu, Lanzhou, China):4875-82.  360. Chiloiro S, Giampietro A, Visconti F, Rossi L, Donfrancesco F, Fleseriu CM, et al. Glucose metabolism outcomes in acromegaly patients on treatment with pasireotide-LAR or pasireotide-LAR plus Pegvisomant. Endocrine. 2021;73(3):658-66.  361. Coletta DK, Hlusko LJ, Scott GR, Garcia LA, Vachon CM, Norman AD, et al. Association of EDARV370A with breast density and metabolic syndrome in Latinos. PLoS ONE. 2021;16(10 October).  362. da Rocha AF, Pereira Junior PS, Calefi GS, Marquezine GF, Morimoto HK, Mazzuco TL, et al. Growth hormone directly favors hepatic ketogenesis in persons with prediabetes or type 2 diabetes mellitus treated with empagliflozin. Endocrine. 2021;73(2):325-30.  363. Gong Y, Fan Z, Zhang P, Qian Y, Huang Q, Deng S, et al. High pre-operative fasting blood glucose levels predict a poor prognosis in patients with pancreatic neuroendocrine tumour. Endocrine. 2021;71(2):494-501.  364. Hempel S, Oehme F, Tahirukaj E, Kolbinger FR, Müssle B, Welsch T, et al. More is More? Total Pancreatectomy for Periampullary Cancer as an Alternative in Patients with High-Risk Pancreatic Anastomosis: A Propensity Score-Matched Analysis. Annals of Surgical Oncology. 2021;28(13):8309-17.  365. Ito T, Fujimori N, Honma Y, Kudo A, Hijioka S, Katsushima S, et al. Long-term safety and efficacy of lanreotide autogel in Japanese patients with neuroendocrine tumors: Final results of a phase II open-label extension study. Asia-Pacific Journal of Clinical Oncology. 2021;17(5):e153-e61.  366. Kumaran S, Unnikrishnan AG. Fibrocalculous pancreatic diabetes. Journal of Diabetes and its Complications. 2021;35(1).  367. Li M, Zhao Z, Qin G, Chen L, Lu J, Huo Y, et al. Non-alcoholic fatty liver disease, metabolic goal achievement with incident cardiovascular disease and eGFR-based chronic kidney disease in patients with prediabetes and diabetes. Metabolism: Clinical and Experimental. 2021;124((Li M.; Zhao Z.; Lu J.; Xu M.; Chen Y.; Wang T.; Wang S.; Ning G.; Bi Y., byf10784@rjh.com.cn; Xu Y., jane.yuxu@gmail.com; Wang W., wqingw61@163.com) Department of Endocrine and Metabolic Diseases, Shanghai Institute of Endocrine and Metabolic Diseases, Shanghai National Clinical Research Center for Metabolic Diseases, Key Laboratory for Endocrine and Metabolic Diseases of the National Health Commission of the People's Republic of China, Shanghai National Center for Translational Medicine, Ruijin Hospital, Shanghai Jiaotong University School of Medicine, Shanghai, China).  368. Lindsay RS, Whitesell JC, Dew KE, Rodriguez E, Sandor AM, Tracy D, et al. MERTK on mononuclear phagocytes regulates T cell antigen recognition at autoimmune and tumor sites. Journal of Experimental Medicine. 2021;218(10).  369. Ma J, Xing B, Cao Y, He X, Bennett KE, Tong C, et al. Menin-regulated Pbk controls high fat diet-induced compensatory beta cell proliferation. EMBO Molecular Medicine. 2021;13(5).  370. Menini S, Iacobini C, Vitale M, Pesce C, Pugliese G. Diabetes and pancreatic cancer-a dangerous liaison relying on carbonyl stress. Cancers. 2021;13(2):1-26.  371. Meo SA, Abukhalaf AA, Alomar AA, Alessa OM, Sumaya OY, Meo AS. Prevalence of prediabetes and type 2 diabetes mellitus in football players: A novel multi football clubs cross sectional study. International Journal of Environmental Research and Public Health. 2021;18(4):1-9.  372. Mezza T, Ferraro PM, Giuseppe GD, Moffa S, Cefalo CMA, Cinti F, et al. Pancreaticoduodenectomy model demonstrates a fundamental role of dysfunctional β cells in predicting diabetes. Journal of Clinical Investigation. 2021;131(12).  373. Neves JS, Teles L, Guerreiro V, Lau E, Oliveira AI, Graça L, et al. Clinical characteristics and incidence of glucose metabolism disorders during the follow-up of surgically treated insulinomas. Endocrine. 2021;71(2):351-6.  374. Ose DJ, Viskochil R, Holowatyj AN, Larson M, Wilson D, Dunson WA, et al. Understanding the prevalence of prediabetes and diabetes in patients with cancer in clinical practice: A real-world cohort study. JNCCN Journal of the National Comprehensive Cancer Network. 2021;19(6):709-18.  375. Pratheeksha, Chandran V, Rao GM, Rao D. Correlation of CA 19-9 with HbA1c in prediabetic and diabetic groups. Biomedicine (India). 2021;41(4):737-41.  376. Si Y, Wang A, Yang Y, Liu H, Gu S, Mu Y, et al. Fasting Blood Glucose and 2-h Postprandial Blood Glucose Predict Hypertension: A Report from the REACTION Study. Diabetes Therapy. 2021;12(4):1117-28.  377. Singla M, Saini JK. Diabetes mellitus of pituitary origin: A case report. touchREVIEWS in Endocrinology. 2021;17(1):68-70.  378. Smith SM, Boppana A, Traupman JA, Unson E, Maddock DA, Chao K, et al. Impaired glucose metabolism in patients with diabetes, prediabetes, and obesity is associated with severe COVID-19. Journal of Medical Virology. 2021;93(1):409-15.  379. Speaker SL, Rastogi R, Sussman TA, Hu B, Misra-Hebert AD, Rothberg MB. Treatment of Patients with Prediabetes in a Primary Care Setting 2011–2018: an Observational Study. Journal of General Internal Medicine. 2021;36(4):923-9.  380. Stentz FB, Mikhael A, Kineish O, Christman J, Sands C. High protein diet leads to prediabetes remission and positive changes in incretins and cardiovascular risk factors. Nutrition, Metabolism and Cardiovascular Diseases. 2021;31(4):1227-37.  381. Sun L, Fang W, Yi D, Sun W, Wang C. Analysis of the clinical characteristics of insulin autoimmune syndrome induced by methimazole. Journal of Clinical Pharmacy and Therapeutics. 2021;46(2):470-5.  382. Takenaga K, Akimoto M, Koshikawa N, Nagase H. Obesity reduces the anticancer effect of AdipoRon against orthotopic pancreatic cancer in diet-induced obese mice. Scientific reports. 2021;11(1):2923.  383. Tsai K, Yu AC, Livhits MJ, Sajed D, Leung AM, Cheung DS. Systemic light-chain amyloidosis incidentally diagnosed after subtotal parathyroidectomy and thyroid lobectomy. BMJ Case Reports. 2021;14(4).  384. Villanueva-Rodríguez LG, Reza-Albarrán AA, Cárdenas E, Ángeles A, Lester J. Impaired glucose tolerance with neurological manifestations in insulinoma. Clinical Case Reports. 2021;9(9).  385. Weaver JR, Odanga JJ, Breathwaite EK, Treadwell ML, Murchinson AC, Walters G, et al. An increase in inflammation and islet dysfunction is a feature of prediabetes. Diabetes/Metabolism Research and Reviews. 2021;37(6).  386. Wen J, Liu Q, Liu M, Wang B, Li M, Wang M, et al. Increasing Imbalance of Treg/Th17 Indicates More Severe Glucose Metabolism Dysfunction in Overweight/obese Patients. Archives of Medical Research. 2021;52(3):339-47.  387. Wilkin C, Colonval M, Dehairs J, Esser N, Iovino M, Gianfrancesco MA, et al. New insights on the pbmcs phospholipidome in obesity demonstrate modulations associated with insulin resistance and glycemic status. Nutrients. 2021;13(10).  388. Witek P, Bolanowski M, Szamotulska K, Wojciechowska-Luźniak A, Jawiarczyk-Przybyłowska A, Kałużny M. The Effect of 6 Months’ Treatment With Pasireotide LAR on Glucose Metabolism in Patients With Resistant Acromegaly in Real-World Clinical Settings. Frontiers in Endocrinology. 2021;12((Witek P.; Wojciechowska-Luźniak A.) Department of Internal Medicine, Endocrinology and Diabetes, Mazovian Bródno Hospital, Medical University of Warsaw, Warsaw, Poland).  389. Xia X, Xue S, Yang G, Li Y, Liu H, Chen C, et al. Association of serum fetuin-B with insulin resistance and pre-diabetes in young Chinese women: evidence from a cross-sectional study and effect of liraglutide. PeerJ. 2021;9((Xia X.; Li L., liling@cqmu.edu.cn) The Key Laboratory of Laboratory Medical Diagnostics in the Ministry of Education and Department of Clinical Biochemistry, College of Laboratory Medicine, Chongqing Medical University, Chongqing, China).  390. Alrawashdeh AY, Al Shhab MA, Zihlif MA. High Throughput Study for Molecular Mechanism of Metformin Pre-Diabetic Protection via Microarray Approach. Endocrine, Metabolic and Immune Disorders - Drug Targets. 2022;22(1):71-99.  391. Alsahhaf A, Alali Y, Albeshri S, Subayt AKA, Alomayri A, Abduljabbar T, et al. Clinical, Radiographic, and Inflammatory Peri-Implant Parameters around Narrow Diameter Implant Crowns among Prediabetic and Non-Diabetic Subjects. Medicina (Kaunas, Lithuania). 2022;58(12).  392. Amisi CA. Markers of insulin resistance in Polycystic ovary syndrome women: An update. World Journal of Diabetes. 2022;13(3):129-49.  393. Araujo-Castro M, Ojeda CM, Centeno RG, López-García MC, Lamas C, Hanzu FA, et al. Glycemic disorders in patients with pheochromocytomas and sympathetic paragangliomas. Endocrine-Related Cancer. 2022;29(12):645-55.  394. Beunen K, Vercauter L, Van Crombrugge P, Moyson C, Verhaeghe J, Vandeginste S, et al. Type 1 diabetes-related autoimmune antibodies in women with gestational diabetes mellitus and the long-term risk for glucose intolerance. Frontiers in Endocrinology. 2022;13((Beunen K., kaat.beunen@kuleuven.be; Moyson C.; Mathieu C.; Benhalima K.) Department of Endocrinology, University Hospitals Gasthuisberg, KU Leuven, Leuven, Belgium).  395. Boursi B, Finkelman B, Giantonio BJ, Haynes K, Rustgi AK, Rhim AD, et al. A clinical prediction model to assess risk for pancreatic cancer among patients with prediabetes. European Journal of Gastroenterology and Hepatology. 2022;34(1):33-8.  396. Cao M, Isaac R, Yan W, Ruan X, Jiang L, Wan Y, et al. Cancer-cell-secreted extracellular vesicles suppress insulin secretion through miR-122 to impair systemic glucose homeostasis and contribute to tumour growth. Nature Cell Biology. 2022;24(6):954-67.  397. Cao Q, Xin Z, He R, Wang T, Xu M, Lu J, et al. Age-specific difference in the association between prediabetes and subclinical atherosclerosis: an analysis of a chinese prospective cohort study. Cardiovascular Diabetology. 2022;21(1).  398. Cao Y, Feng Z, He X, Zhang X, Xing B, Wu Y, et al. Prolactin-regulated Pbk is involved in pregnancy-induced β-cell proliferation in mice. Journal of Endocrinology. 2022;252(2):107-23.  399. Deng J, Guo Y, Gu J, Du J, Kong L, Tao B, et al. The Role of Diabetes Mellitus in the Malignant Pancreatic Cyst Neoplasm Diagnosis and Prognosis. Cancer Management and Research. 2022;14((Deng J.; Guo Y.; Gu J.; Du J.; Kong L.; Tao B.; Li J., liji@huashan.org.cn; Fu D.) Department of Pancreatic Surgery, Pancreatic Disease Institute, Huashan Hospital, Fudan University, Shanghai, China):2091-104.  400. Díaz-Prieto LE, Gómez-Martínez S, Vicente-Castro I, Heredia C, González-Romero EA, Martín-Ridaura MC, et al. Effects of Moringa oleifera Lam. Supplementation on Inflammatory and Cardiometabolic Markers in Subjects with Prediabetes. Nutrients. 2022;14(9).  401. Fjeld K, Gravdal A, Brekke RS, Alam J, Wilhelm SJ, El Jellas K, et al. The genetic risk factor CEL-HYB1 causes proteotoxicity and chronic pancreatitis in mice. Pancreatology. 2022;22(8):1099-111.  402. González-Viveros N, Castro-Ramos J, Gómez-Gil P, Cerecedo-Núñez HH, Gutiérrez-Delgado F, Torres-Rasgado E, et al. Quantification of glycated hemoglobin and glucose in vivo using Raman spectroscopy and artificial neural networks. Lasers in Medical Science. 2022;37(9):3537-49.  403. He S, Wang J, Shen X, Qian X, An Y, Gong Q, et al. Cancer and its predictors in Chinese adults with newly diagnosed diabetes and impaired glucose tolerance (IGT): a 30-year follow-up of the Da Qing IGT and Diabetes Study. British Journal of Cancer. 2022;127(1):102-8.  404. Helminen O, Pokka T, Aspholm S, Ilonen J, Simell OG, Knip M, et al. First-emerging islet autoantibody and glucose metabolism: search for type 1 diabetes subtypes. Endocrine Connections. 2022;11(9).  405. Herndon J, Kaur RJ, Romportl M, Smith E, Koenigs A, Partlow B, et al. The Effect of Curative Treatment on Hyperglycemia in Patients with Cushing Syndrome. Journal of the Endocrine Society. 2022;6(1).  406. Hong H, Lin X, Xu Y, Tong T, Zhang J, He H, et al. Cadmium induces ferroptosis mediated inflammation by activating Gpx4/Ager/p65 axis in pancreatic β-cells. Science of the Total Environment. 2022;849((Hong H.; Lin X.; Xu Y.; Tong T.; Zhang J.; He H.; Zhou Z., lunazhou@zju.edu.cn) Department of Emergency Medicine of First Affiliated Hospital and Department of Environmental Medicine, Zhejiang University School of Medicine, Hangzhou, China).  407. Hossain MM, Paul S, Das M, Saha T, Faruque MO, Hassan Z. Tumour necrosis factor-α −308G/A polymorphism is associated with insulin secretory defects in Bangladeshi prediabetic/diabetic subjects. Journal of Taibah University Medical Sciences. 2022;17(2):241-7.  408. Huang J, Ding X, Ma Y, Li S, Peng Y. The Value of Asprosin on the Development in Prediabetic Individuals: A Prospective Study. Journal of Biological Regulators and Homeostatic Agents. 2022;36(4):1091-8.  409. Infante M, Fabbri A, Padilla N, Pacifici F, Di Perna P, Vitiello L, et al. BNT162b2 mRNA COVID-19 Vaccine Does Not Impact the Honeymoon Phase in Type 1 Diabetes: A Case Report. Vaccines. 2022;10(7).  410. Jende JME, Kender Z, Morgenstern J, Renn P, Mooshage C, Juerchott A, et al. Fractional Anisotropy and Troponin T Parallel Structural Nerve Damage at the Upper Extremities in a Group of Patients With Prediabetes and Type 2 Diabetes – A Study Using 3T Magnetic Resonance Neurography. Frontiers in Neuroscience. 2022;15((Jende J.M.E.; Renn P.; Juerchott A.; Bendszus M.; Kurz F.T., felix.kurz@med.uni-heidelberg.de) Department of Neuroradiology, Heidelberg University Hospital, Heidelberg, Germany).  411. Jiang Y, Xia J, Che C, Wei Y. Data-driven classification of prediabetes using cardiometabolic biomarkers: Data from National Health and Nutrition Examination Survey 2007–2016. Frontiers in Endocrinology. 2022;13((Jiang Y.; Che C.) Medical Department, Hwa Mei Hospital, University of Chinese Academy of Sciences, Ningbo, China).  412. Kim JH, Lim JS. The association between C-reactive protein, metabolic syndrome, and prediabetes in Korean children and adolescents. Annals of Pediatric Endocrinology and Metabolism. 2022;27(4):273-80.  413. Kim YJ, Oh CM, Park SK, Jung JY, Kim MH, Ha E, et al. Fasting blood glucose and risk of incident pancreatic cancer. PLoS ONE. 2022;17(10 October).  414. Kirthi V, Zuckerman BP, Alam U, Bunce C, Hopkins D, Jackson TL. ASSOCIATIONS BETWEEN DYSGLYCEMIA, RETINAL NEURODEGENERATION, AND MICROALBUMINURIA IN PREDIABETES AND TYPE 2 DIABETES. Retina. 2022;42(3):442-9.  415. Li J, Gower B, McLain A, Yarar-Fisher C. Effects of a low-carbohydrate/high-protein diet on metabolic health in individuals with chronic spinal cord injury: An exploratory analysis of results from a randomized controlled trial. Physiological Reports. 2022;10(22).  416. Nakamae H, Yamamoto M, Sakaida E, Kanda Y, Ohmine K, Ono T, et al. Nilotinib vs. imatinib in Japanese patients with newly diagnosed chronic myeloid leukemia in chronic phase: 10-year follow‑up of the Japanese subgroup of the randomized ENESTnd trial. International Journal of Hematology. 2022;115(1):33-42.  417. Nicolaisen SK, Thomsen RW, Lau CJ, Sørensen HT, Pedersen L. Development of a 5-year risk prediction model for type 2 diabetes in individuals with incident HbA1c-defined pre-diabetes in Denmark. BMJ Open Diabetes Research and Care. 2022;10(5).  418. Oh H, Park HJ, Oh J, Lee ES, Park SB, Cha MJ, et al. Hyperechoic pancreas on ultrasonography: an analysis of its severity and clinical implications. Ultrasonography. 2022;41(2):335-43.  419. Piñeros AR, Kulkarni A, Gao H, Orr KS, Glenn L, Huang F, et al. Proinflammatory signaling in islet β cells propagates invasion of pathogenic immune cells in autoimmune diabetes. Cell Reports. 2022;39(13).  420. Roest S, Goedendorp-Sluimer MM, Köbben JJ, Constantinescu AA, Taverne YJHJ, Zijlstra F, et al. Oral Glucose Tolerance Test for the Screening of Glucose Intolerance Long Term Post‐Heart Transplantation. Transplant International. 2022;35((Roest S.; Goedendorp-Sluimer M.M.; Constantinescu A.A.; Zijlstra F.; Manintveld O.C., o.manintveld@erasmusmc.nl) Department of Cardiology, Thorax Center, Erasmus MC, University Medical Center Rotterdam, Rotterdam, Netherlands).  421. Ryrsø CK, Dungu AM, Hegelund MH, Jensen AV, Sejdic A, Faurholt-Jepsen D, et al. Body composition, physical capacity, and immuno-metabolic profile in community-acquired pneumonia caused by COVID-19, influenza, and bacteria: a prospective cohort study. International Journal of Obesity. 2022;46(4):817-24.  422. Saleh M, Kim JY, March C, Gebara N, Arslanian S. Youth prediabetes and type 2 diabetes: Risk factors and prevalence of dysglycaemia. Pediatric Obesity. 2022;17(1).  423. Samin KA, Ullah S, Amjad Z, Kumar A, Jabbar U, Ibrahim MT. Prevalence of Diabetes Mellitus and Impaired Glucose Tolerance in Patients with Covid-19. Pakistan Journal of Medical and Health Sciences. 2022;6(1):1131-4.  424. Schlesinger S, Neuenschwander M, Barbaresko J, Lang A, Maalmi H, Rathmann W, et al. Prediabetes and risk of mortality, diabetes-related complications and comorbidities: umbrella review of meta-analyses of prospective studies. Diabetologia. 2022;65(2):275-85.  425. Shakya P, Shrestha A, Karmacharya BM, Kulseng BE, Skovlund E, Sen A. Prevalence of prediabetes and associated factors of prediabetic stages: A cross-sectional study among adults in Nepal. BMJ Open. 2022;12(12).  426. Sipe AT, Neuhouser ML, Breymeyer KL, Utzschneider KM. Effect of Dietary Glycemic Index on β-Cell Function in Prediabetes: A Randomized Controlled Feeding Study. Nutrients. 2022;14(4).  427. Stoica RA, Drăgana N, Ancuceanu R, Geicu OI, Guja C, Pantea-Stoian A, et al. Interleukin-8, CXCL10, CXCL11 and their role in insulin resistance in adult females with subclinical hypothyroidism and prediabetes. Journal of Clinical and Translational Endocrinology. 2022;28((Stoica R.A., roxana88stoica@gmail.com; Guja C.; Pantea-Stoian A.; Serafinceanu C.; Ionescu-Tîrgoviște C.) Department of Diabetes, Nutrition and Metabolic Diseases, “Carol Davila” University of Medicine and Pharmacy, Bucharest, Romania).  428. Tan Q, Wang X, Chen C, Liu X, Chen Y, Tan C. Prognostic value of preoperative diabetes mellitus in patients with non-functional pancreatic neuroendocrine neoplasms. American Journal of Surgery. 2022;224(4):1162-7.  429. Vasepalli P, Noor MT, Thakur BS. Hepatogenous Diabetes - A Report from Central India. Journal of Clinical and Experimental Hepatology. 2022;12(2):312-8.  430. Wu TE, Chen HS. The role of growth hormone and IGF-1 in retinopathy: a prospective study of retinopathy in patients with acromegaly and impaired fasting glucose. Diabetology and Metabolic Syndrome. 2022;14(1).  431. Xu W, Song Q, Wang X, Zhao Z, Meng X, Xia C, et al. Association of stress hyperglycemia ratio and in-hospital mortality in patients with coronary artery disease: insights from a large cohort study. Cardiovascular Diabetology. 2022;21(1).  432. Yang J, Zhang J, Wang R, Liu Y, Chen Y. Prevalence of dysglycemia and associated risk factors in patients with pancreatic benign and low-grade malignant tumors before pancreatic surgery: A prospective observational study. Frontiers in Endocrinology. 2022;13((Yang J.; Wang R.; Liu Y.; Chen Y., chenyonghua2007@163.com) Department of Pancreatic Surgery, West China Hospital of Sichuan University, Chengdu, China).  433. Yoo TK, Lee MY, Lee SA, Cheong ES, Seo MH, Sung KC. Association of Glycosylated Hemoglobin Level and Cancer-Related Mortality in Patients without Diabetes. Journal of Clinical Medicine. 2022;11(19).  434. Yue C, Zhang C, Ying C, Jiang H. Diabetes associated with cervical carcinoma among high-risk HPV-infected patients with cytologically diagnosed high grade squamous intraepithelial lesion. Frontiers in Endocrinology. 2022;13((Yue C.; Zhang C.; Ying C., ycmzh2012@163.com; Jiang H., jianghua@fudan.edu.cn) Obstetrics and Gynecology Hospital of Fudan University, Shanghai, China).  435. Zhang P, Xiao Z, Xu H, Zhu X, Wang L, Huang D, et al. Hyperglycemia is associated with adverse prognosis in patients with pancreatic neuroendocrine neoplasms. Endocrine. 2022;77(2):262-71.  436. Ali MK, Imperatore G, Benoit SR, O'Brien MJ, Holliday CS, Echouffo-Tcheugui JB, et al. Impact of changes in diabetes screening guidelines on testing eligibility and potential yield among adults without diagnosed diabetes in the United States. Diabetes Research and Clinical Practice. 2023;197((Ali M.K., mkali@emory.edu; Imperatore G.; Benoit S.R.; Holliday C.S.; McKeever Bullard K.) Division of Diabetes Translation, Centers for Disease Control and Prevention, Atlanta, GA, United States).  437. Anuradha N. ASSOCIATION OF THE IMPACT OF H. PYLORI INFECTION ON DIABETIC AND LIPID PROFILE IN PRE-DIABETIC PATIENTS. International Journal of Academic Medicine and Pharmacy. 2023;5(1):381-5.  438. Balaban DV, Coman L, Balaban M, Zoican A, Pușcașu DA, Ayatollahi S, et al. Glycemic Abnormalities in Pancreatic Cystic Lesions—A Single-Center Retrospective Analysis. Gastroenterology Insights. 2023;14(2):191-203.  439. Basavarajappa HD, Irimia JM, Bauer BM, Fueger PT. The Adaptor Protein NumbL Is Involved in the Control of Glucolipotoxicity-Induced Pancreatic Beta Cell Apoptosis. International Journal of Molecular Sciences. 2023;24(4).  440. Bejjani J, Papachristou GI, Dungan K, Evans Phillips A, Singh V, Toledo FG, et al. Incident diabetes following acute pancreatitis in a multicenter prospective observational cohort. Pancreatology. 2023;23(8):900-3.  441. Chen G, Zhang R, Tan C, Liu X, Yu L, Chen Y. Optimal glycated hemoglobin A1c value for prediabetes and diabetes in patients with pancreatic diseases. Frontiers in Endocrinology. 2023;14((Chen G.; Tan C.; Liu X.; Chen Y., chenyonghua2007@163.com) Division of Pancreatic Surgery, Department of General Surgery, West China Hospital, Sichuan University, Chengdu, China).  442. Cheng Y, Zhu H, Ren J, Wu HY, Yu JE, Jin LY, et al. Follicle-stimulating hormone orchestrates glucose-stimulated insulin secretion of pancreatic islets. Nature Communications. 2023;14(1).  443. Fiffer MR, Li H, Iyer HS, Nethery RC, Sun Q, James P, et al. Associations between air pollution, residential greenness, and glycated hemoglobin (HbA1c) in three prospective cohorts of U.S. adults. Environmental Research. 2023;239((Fiffer M.R., MFiffe2@uic.edu; Li H.; James P.; Hart J.E.; Laden F.) Harvard T.H. Chan School of Public Health, Department of Environmental Health, Boston, MA, United States).  444. Fukushima T, Chubachi S, Namkoong H, Asakura T, Tanaka H, Lee H, et al. Clinical significance of prediabetes, undiagnosed diabetes and diagnosed diabetes on critical outcomes in COVID-19: Integrative analysis from the Japan COVID-19 task force. Diabetes, Obesity and Metabolism. 2023;25(1):144-55.  445. Gu ZJ, Song QJ, Gu WQ, Zhang GP, Su Y, Tang Y, et al. New approaches in the diagnosis and prognosis of gestational diabetes mellitus. European Review for Medical and Pharmacological Sciences. 2023;27(21):10583-94.  446. Harun R, Sane R, Yoshida K, Sutaria DS, Jin JY, Lu J. Risk Factors of Hyperglycemia After Treatment With the AKT Inhibitor Ipatasertib in the Prostate Cancer Setting: A Machine Learning-Based Investigation. JCO clinical cancer informatics. 2023;7((Harun R.; Sane R.; Yoshida K.; Sutaria D.S.; Jin J.Y.; Lu J.) Genentech Inc, South San Francisco, CA, United States):e2200168.  447. Hirano Y, Fujita T, Konishi T, Takemura R, Sato K, Kurita D, et al. Impact of pre-diabetes, well-controlled diabetes, and poorly controlled diabetes on anastomotic leakage after esophagectomy for esophageal cancer: a two-center retrospective cohort study of 1901 patients. Esophagus. 2023;20(2):246-55.  448. Kasujja FX, Nuwaha F, Ekirapa EK, Kusolo R, Mayega RW. The association between asymptomatic malaria and blood glucose among outpatients in a rural low-income setting. Diabetes Epidemiology and Management. 2023;9((Kasujja F.X., fxkasujja@musph.ac.ug; Kusolo R.; Mayega R.W.) Department of Epidemiology and Biostatistics, School of Public Health, College of Health Sciences, Makerere University, Kampala, Uganda).  449. Koo DH, Han K, Park CY. Impact of cumulative hyperglycemic burden on the pancreatic cancer risk: A nationwide cohort study. Diabetes Research and Clinical Practice. 2023;195((Koo D.-H.) Division of Hematology/Oncology, Department of Internal Medicine, Kangbuk Samsung Hospital, Sungkyunkwan University School of Medicine, Seoul, South Korea).  450. Lee YL, Ting TH, Lim CT, Arrumugam-Arthini C, Karuppiah T, Ling KH. Novel PAX4 variant in a child and family with diabetes mellitus - case report and review of the literature. Journal of Pediatric Endocrinology and Metabolism. 2023;36(10):988-92.  451. Lin D, Yu J, Lin L, Ou Q, Quan H. MRPS6 modulates glucose-stimulated insulin secretion in mouse islet cells through mitochondrial unfolded protein response. Scientific reports. 2023;13(1):16173.  452. Liu J, Fu H, Kang F, Ning G, Ni Q, Wang W, et al. β-Cell glucokinase expression was increased in type 2 diabetes subjects with better glycemic control. Journal of Diabetes. 2023;15(5):409-18.  453. Liu JB, Gu QB, Liu P. Inflammatory myofibroblastic tumor of the pancreatic neck misdiagnosed as neuroendocrine tumor: A case report. World Journal of Gastroenterology. 2023;29(20):3216-21.  454. Masuda S, Yamada T, Hanzawa N. Impact of prediabetes with a high risk of diabetes stratified by glycated hemoglobin level on the severity of coronavirus disease 2019 during admission. Diabetology International. 2023;14(4):372-80.  455. Meloni A, Nobile M, Keilberg P, Positano V, Santarelli MF, Pistoia L, et al. Pancreatic fatty replacement as risk marker for altered glucose metabolism and cardiac iron and complications in thalassemia major. European Radiology. 2023;33(10):7215-25.  456. Osher E, Geva R, Wolf I, Tordjman K, Klausner J, Sofer Y, et al. Dysglycemia in non-functioning pancreatic neuroendocrine tumors (NF-PNET): Further insights into an under recognized entity. Journal of Clinical and Translational Endocrinology. 2023;33((Osher E., esteros@tlvmc.gov.il; Tordjman K.; Sofer Y.; Stern N.; Greenman Y.) Institute of Endocrinology, Metabolism, and Hypertension, Tel Aviv-Sourasky Medical Center, Faculty of Medicine, Tel Aviv University, Israel).  457. Pagán L, Yang W, Shao H, Wang Y, Zhang P. Medical expenditure trajectory and HbA1c progression prior to and after clinical diagnosis of type 2 diabetes in a commercially insured population in the USA. BMJ Open Diabetes Research and Care. 2023;11(6).  458. Pausch TM, Liu X, Dincher J, Contin P, Cui J, Wei J, et al. Middle Segment-Preserving Pancreatectomy to Avoid Pancreatic Insufficiency: Individual Patient Data Analysis of All Published Cases from 2003–2021. Journal of Clinical Medicine. 2023;12(5).  459. Peng Y, Wang P, Gong J, Liu F, Qiao Y, Si C, et al. Association between the Finnish Diabetes Risk Score and cancer in middle-aged and older adults: Involvement of inflammation. Metabolism: Clinical and Experimental. 2023;144((Peng Y.; Wang P.; Gong J.; Liu F.; Qiao Y.; Si C.; Wang X.; Zhou H.; Song F., songfangfang@tmu.edu.cn) Department of Epidemiology and Biostatistics, Key Laboratory of Molecular Cancer Epidemiology, Tianjin, National Clinical Research Center for Cancer, Tianjin's Clinical Research Center for Cancer, Tianjin Medical University Cancer Institute and Hospital, Tianjin, China).  460. Quintas J, Mowatt KB, Mullally JA, Steinberg A. New-onset persistent hyperglycemia with initiation of brentuximab treatment. Journal of Oncology Pharmacy Practice. 2023((Quintas J., joseph.quintas@wmchealth.org) Internal Medicine, Westchester Medical Center, Valhalla, NY, United States).  461. Ritsinger V, Hagström E, Hambraeus K, James S, Jernberg T, Lagerqvist B, et al. Design and rationale of the myocardial infarction and new treatment with metformin study (MIMET) - Study protocol for a registry-based randomised clinical trial. Journal of Diabetes and its Complications. 2023;37(10).  462. Sabharwal I, Bawa A, Kaur S. The Influence of Inflammation on HBA1C Levels and Insulin Resistance in Prediabetes: Insights from Inflammatory Biomarkers. International Journal of Pharmaceutical and Clinical Research. 2023;15(8):1054-8.  463. Sandforth A, von Schwartzenberg RJ, Arreola EV, Hanson RL, Sancar G, Katzenstein S, et al. Mechanisms of weight loss-induced remission in people with prediabetes: a post-hoc analysis of the randomised, controlled, multicentre Prediabetes Lifestyle Intervention Study (PLIS). The Lancet Diabetes and Endocrinology. 2023;11(11):798-810.  464. Shah I, Silva-Santisteban A, Germansky KA, Trindade A, Raphael KL, Kushnir V, et al. Pancreatic Cancer Screening for At-Risk Individuals (Pancreas Scan Study): Yield, Harms, and Outcomes from a Prospective Multicenter Study. American Journal of Gastroenterology. 2023;118(9):1664-70.  465. Shaheen M, Schrode KM, Tedlos M, Pan D, Najjar SM, Friedman TC. Racial/ethnic and gender disparity in the severity of NAFLD among people with diabetes or prediabetes. Frontiers in Physiology. 2023;14((Shaheen M., magdashaheen@cdrewu.edu; Schrode K.M.; Tedlos M.; Pan D.; Friedman T.C.) Charles R. Drew University, Los Angeles, CA, United States).  466. Swauger SE, Fashho K, Hornung LN, Elder DA, Thapaliya S, Anton CG, et al. Association of pancreatic fat on imaging with pediatric metabolic co-morbidities. Pediatric Radiology. 2023;53(10):2030-9.  467. Tanaka H, Matsusaki S, Asakawa H, Tsuruga S, Nose K, Kumazawa H, et al. A Novel Scoring System to Improve the Detection Efficiency of Pancreatic Cystic Lesions in the General Population. Internal Medicine. 2023;62(3):335-44.  468. Tanaka S, Akagawa H, Azuma K, Watanabe K, Higuchi S, Iwasaki N. A novel pathogenic variant in the glucokinase gene found in two Japanese siblings with maturity-onset diabetes of the young 2. Endocrine Journal. 2023;70(6):629-34.  469. Thongsroy J, Mutirangura A. Decreased Alu methylation in type 2 diabetes mellitus patients increases HbA1c levels. Journal of Clinical Laboratory Analysis. 2023;37(17-18).  470. Treiber G, Guilleux A, Huynh K, Bonfanti O, Flaus–Furmaniuk A, Couret D, et al. Lipoatrophic diabetes in familial partial lipodystrophy type 2: From insulin resistance to diabetes. Diabetes and Metabolism. 2023;49(2).  471. Varun K, Zoltan K, Alba S, Manuel B, Elisabeth K, Dimitrios T, et al. Elevated markers of DNA damage and senescence are associated with the progression of albuminuria and restrictive lung disease in patients with type 2 diabetes. eBioMedicine. 2023;90((Varun K.; Zoltan K.; Alba S.; Manuel B.; Elisabeth K.; Dimitrios T.; Jan B G.; Maik B.; Thomas F.; Julia S.; Peter N.; Stefan K., stefan.kopf@med.uni-heidelberg.de) Department of Endocrinology, Diabetology and Clinical Chemistry (Internal Medicine I), University Hospital of Heidelberg, Heidelberg, Germany).  472. Wang Z, Yu D, Osuagwu UL, Pickering K, Baker J, Cutfield R, et al. Health Inequality in Eight Adverse Outcomes Over a 25-Year Period in a Multi-Ethnic Population in New Zealand Population with Impaired Glucose Tolerance and/or Impaired Fasting Glucose: An Age-Period-Cohort Analysis. Clinical Epidemiology. 2023;15((Wang Z.; Yu D.; Cai Y.; Zhao Z., zhanzhengzhao@zzu.edu.cn; Simmons D., Da.Simmons@westernsydney.edu.au) Department of Nephrology, The First Affiliated Hospital, Zhengzhou University, Zhengzhou, China):1123-43.  473. Yang J, Tan C, Zheng Z, Wang X, Liu X, Chen Y. Elevated Bile Acid Is Associated with Worsened Impaired Glucose Homeostasis in Pancreatic Ductal Adenocarcinoma Patients with Extrahepatic Cholestasis through Increased Hepatic Insulin Clearance. Journal of Clinical Medicine. 2023;12(6).  474. Zhang J, Olsen A, Halkjær J, Petersen KE, Tjønneland A, Overvad K, et al. Self-reported and measured anthropometric variables in association with cardiometabolic markers: A Danish cohort study. PLoS ONE. 2023;18(7 July).  475. Zhang X, Wu H, Fan B, Shi M, Lau ESH, Yang A, et al. The role of age on the risk relationship between prediabetes and major morbidities and mortality: Analysis of the Hong Kong diabetes surveillance database of 2 million Chinese adults: Age, prediabetes and major clinical events. The Lancet Regional Health - Western Pacific. 2023;30((Zhang X.; Wu H.; Fan B.; Shi M.; Lau E.S.H.; Yang A.; Chow E.; Kong A.P.S.; Chan J.C.N.; Ma R.C.W., rcwma@cuhk.edu.hk; Luk A.O.Y., andrealuk@cuhk.edu.hk) Department of Medicine and Therapeutics, The Chinese University of Hong Kong, Hong Kong Special Administrative Region, Hong Kong).  476. Zheng R, Xu Y, Li M, Gao Z, Wang G, Hou X, et al. Data-driven subgroups of prediabetes and the associations with outcomes in Chinese adults. Cell Reports Medicine. 2023;4(3).  477. Zhu L, Wang S, Sun Z, Liu J, Dai M, Han X, et al. Extracellular volume fraction of the pancreas predicts glucose intolerance in patients undergoing major pancreatic surgeries. European Journal of Radiology. 2023;164((Zhu L., zhuliang_pumc@163.com; Wang S.; Sun Z.; Liu J.; Xue H.; Jin Z.) Department of Radiology, Peking Union Medical College Hospital, Beijing, China).  478. Candemir B, Kisip K, Akın, Tuba Sanal H, Taşar M, Altunkaynak B, et al. Pancreatosteatosis in patients with adrenal incidentaloma: A risk factor for impaired glucose metabolism. Diabetes Research and Clinical Practice. 2024;208((Candemir B., bilalogluburcu@gmail.com; Akın; Ersöz Gülçelik N.) University of Health Sciences, Gulhane Faculty of Medicine, Department of Endocrinology and Metabolism, Ankara, Turkey).  479. Hua J, Lin H, Wang X, Qian ZM, Vaughn MG, Tabet M, et al. Associations of glycosylated hemoglobin, pre-diabetes, and type 2 diabetes with incident lung cancer: A large prospective cohort study. Diabetes and Metabolic Syndrome: Clinical Research and Reviews. 2024;18(2).  480. Melo RH, Pontes AG, Delmanto LRMG, Bueloni-Dias FN, Vespoli HDL, Nahas EAP. The role of glycated hemoglobin in the diagnosis of prediabetes and diabetes mellitus in young women with polycystic ovary syndrome. Clinical Endocrinology. 2024;100(2):124-31.  481. Mukherjee N, Contreras CJ, Lin L, Colglazier KA, Mather EG, Kalwat MA, et al. RIPK3 promotes islet amyloid-induced β-cell loss and glucose intolerance in a humanized mouse model of type 2 diabetes. Molecular Metabolism. 2024;80((Mukherjee N.; Templin A.T., templin@iu.edu) Department of Biochemistry & Molecular Biology, Indiana University School of Medicine, Indianapolis, IN, United States).  482. Saito Y, Sakamoto T, Kobayashi M, Takekuma Y, Higuchi I, Okamoto K, et al. Evaluation of Prediabetes in Cisplatin-induced Nephrotoxicity in the Short Hydration Method: A Subgroup Analysis. In Vivo. 2024;38(2):800-6.  483. Suryakala D, Shaik YA. Study of Non-Alcoholic Fatty Liver Disease in Andhra Pradesh Population. International Journal of Pharmaceutical and Clinical Research. 2024;16(1):271-5.  484. Wang SY, Zhang WS, Jiang CQ, Jin YL, Zhu T, Zhu F, et al. Association of Measures of Glucose Metabolism with Colorectal Cancer Risk in Older Chinese: A 13-Year Follow-up of the Guangzhou Biobank Cohort Study-Cardiovascular Disease Substudy and Meta-Analysis. Diabetes and Metabolism Journal. 2024;48(1):134-45. |
| **Studies excluded in full-text review with reasons (n = 16)** | |
| Cross-sectional studies  (n = 2) | 1. Zhan YS, Feng L, Tang SH, Li WG, Xu M, Liu TF, et al. Glucose metabolism disorders in cancer patients in a Chinese population. Medical Oncology. 2010;27(2):177-84.  2. Brewer MJ, Doucette JT, Bar-Mashiah A, Glickman JW, Kessel E, Aronson A, et al. Glycemic Changes and Weight Loss Precede Pancreatic Ductal Adenocarcinoma by up to 3 Years in a Diverse Population. Clin Gastroenterol Hepatol. 2022;20(5):1105-11 e2. |
| PreD not evaluated as exposure (n = 4) | 1. Stolzenberg-Solomon RZ, Graubard BI, Chari S, Limburg P, Taylor PR, Virtamo J, et al. Insulin, glucose, insulin resistance, and pancreatic cancer in male smokers. JAMA. 2005;294(22):2872-8.  2. Wolpin BM, Bao Y, Qian ZR, Wu C, Kraft P, Ogino S, et al. Hyperglycemia, insulin resistance, impaired pancreatic β-cell function, and risk of pancreatic cancer. J Natl Cancer Inst. 2013;105(14):1027-35.  3. Huang BZ, Pandol SJ, Jeon CY, Chari ST, Sugar CA, Chao CR, et al. New-Onset Diabetes, Longitudinal Trends in Metabolic Markers, and Risk of Pancreatic Cancer in a Heterogeneous Population. Clin Gastroenterol Hepatol. 2020;18(8):1812-21 e7.  4. Wu BU, Butler RK, Lustigova E, Lawrence JM, Chen W. Association of Glycated Hemoglobin Levels With Risk of Pancreatic Cancer. JAMA Netw Open. 2020;3(6):e204945. |
| With no controls of normoglycemia (n = 3) | 1. Park JH, Han K, Hong JY, Park YS, Park JO. Association between alcohol consumption and pancreatic cancer risk differs by glycaemic status: A nationwide cohort study. European Journal of Cancer. 2022;163((Park J.-H.) Department of Family Medicine, Korea University Ansan Hospital, Korea University College of Medicine, Ansan, South Korea):119-27.  2. Jensen MH, Cichosz SL, Hejlesen O, Henriksen SD, Drewes AM, Olesen SS. Risk of pancreatic cancer in people with new-onset diabetes: A Danish nationwide population-based cohort study. Pancreatology. 2023;23(6):642-9.  3. Park JH, Hong JY, Shen JJ, Han K, Park YS, Park JO. Smoking Cessation and Pancreatic Cancer Risk in Individuals With Prediabetes and Diabetes: A Nationwide Cohort Study. JNCCN Journal of the National Comprehensive Cancer Network. 2023;21(11):1149-55. |
| Reports of PC related mortality (n = 4) | 1. Ansary-Moghaddam A, Huxley R, Barzi F, Lawes C, Ohkubo T, Fang X, et al. The effect of modifiable risk factors on pancreatic cancer mortality in populations of the Asia-Pacific region. Cancer Epidemiol Biomarkers Prev. 2006;15(12):2435-40.  2. Zhou XH, Qiao Q, Zethelius B, Pyörälä K, Söderberg S, Pajak A, et al. Diabetes, prediabetes and cancer mortality. Diabetologia. 2010;53(9):1867-76.  3. Nagai M, Murakami Y, Tamakoshi A, Kiyohara Y, Yamada M, Ukawa S, et al. Fasting but not casual blood glucose is associated with pancreatic cancer mortality in Japanese: EPOCH-JAPAN. Cancer Causes and Control. 2017;28(6):625-33.  4. Kim NH, Chang Y, Lee SR, Ryu S, Kim HJ. Glycemic Status, Insulin Resistance, and Risk of Pancreatic Cancer Mortality in Individuals With and Without Diabetes. The American journal of gastroenterology. 2020;115(11):1840-8. |
| Studies with overlapped population (n = 3) | 1. Peila R, Rohan TE. Diabetes, Glycated Hemoglobin, and Risk of Cancer in the UK Biobank Study. Cancer Epidemiol Biomarkers Prev. 2020;29(6):1107-19.  2. Kim K, Kim B, Kim H, Park HS, Ahn YB, Ko SH, et al. The impact of diabetes status on total and site-specific cancer risk in the elderly population: A nationwide cohort study. Diabetes Research and Clinical Practice. 2023;203((Kim K.; Ahn Y.-B.; Ko S.-H.; Yun J.-S., dryun@catholic.ac.kr) Division of Endocrinology and Metabolism, Department of Internal Medicine, St. Vincent's Hospital, College of Medicine, The Catholic University of Korea, Seoul, South Korea).  3. Tran TXM, Kim S, Song H, Park B. Increased risk of cancer and cancer-related mortality in middle-aged Korean women with prediabetes and diabetes: a population-based study. Epidemiology and Health. 2023;45((Tran T.X.M.; Kim S.; Park B., hayejine@hanyang.ac.kr) Department of Preventive Medicine, Hanyang University College of Medicine, Seoul, South Korea):1-10. |
| **Studies included (n = 9)** | |
| Included | Citations |
|  | 1. Jee SH, Ohrr H, Sull JW, Yun JE, Ji M, Samet JM. Fasting serum glucose level and cancer risk in Korean men and women. JAMA. 2005;293(2):194-202.  2. Rapp K, Schroeder J, Klenk J, Ulmer H, Concin H, Diem G, et al. Fasting blood glucose and cancer risk in a cohort of more than 140,000 adults in Austria. Diabetologia. 2006;49(5):945-52.  3. Grote VA, Rohrmann S, Nieters A, Dossus L, Tjonneland A, Halkjaer J, et al. Diabetes mellitus, glycated haemoglobin and C-peptide levels in relation to pancreatic cancer risk: a study within the European Prospective Investigation into Cancer and Nutrition (EPIC) cohort. Diabetologia. 2011;54(12):3037-46.  4. Koo DH, Han KD, Park CY. The Incremental Risk of Pancreatic Cancer According to Fasting Glucose Levels: Nationwide Population-Based Cohort Study. J Clin Endocrinol Metab. 2019;104(10):4594-9.  5. Jacobson S, Dahlqvist P, Johansson M, Svensson J, Billing O, Sund M, et al. Hyperglycemia as a risk factor in pancreatic cancer: A nested case-control study using prediagnostic blood glucose levels. Pancreatology. 2021;21(6):1112-8.  6. Ke J, Lin T, Liu X, Wu K, Ruan X, Ding Y, et al. Glucose Intolerance and Cancer Risk: A Community-Based Prospective Cohort Study in Shanghai, China. Front Oncol. 2021;11:726672.  7. McDonnell D, Cheang AWE, Wilding S, Wild SH, Frampton AE, Byrne CD, et al. Elevated Glycated Haemoglobin (HbA1c) Is Associated with an Increased Risk of Pancreatic Ductal Adenocarcinoma: A UK Biobank Cohort Study. Cancers (Basel). 2023;15(16).  8. Tan PS, Garriga C, Clift A, Liao W, Patone M, Coupland C, et al. Temporality of body mass index, blood tests, comorbidities and medication use as early markers for pancreatic ductal adenocarcinoma (PDAC): a nested case-control study. Gut. 2023;72(3):512-21.  9. Ahn BY, Kim B, Park S, Kim SG, Han K, Cho SJ. Cumulative exposure to impaired fasting glucose and gastrointestinal cancer risk: A nationwide cohort study. Cancer. 2024. |
